# Enantioselective Diels–Alder reaction of anthracene by chiral tritylium catalysis

**DOI:** 10.3762/bjoc.15.129

**Published:** 2019-06-14

**Authors:** Qichao Zhang, Jian Lv, Sanzhong Luo

**Affiliations:** 1 Key Laboratory of Molecular Recognition and Function, Institute of Chemistry, Chinese Academy of Sciences, 100190, Beijing, China; 2 State Key Laboratory Base of Eco-Chemical Engineering, College of Chemistry and Molecular Engineering, Qingdao University of Science & Technology, 266042, Qingdao, China; 3 Center of Basic Molecular Science (CBMS), Department of Chemistry, Tsinghua University, 100084, Beijing, China

**Keywords:** anthracene, carbocation catalysis, Diels–Alder reaction, Fe(III)-based phosphate anion, tritylium salt

## Abstract

The combination of the trityl cation and a chiral weakly coordinating Fe(III)-based bisphosphate anion was used to develop a new type of a highly active carbocation Lewis acid catalyst. The stereocontrol potential of the chiral tritylium ion pair was demonstrated by its application in an enantioselective Diels–Alder reaction of anthracene.

## Introduction

**Scheme 1:**
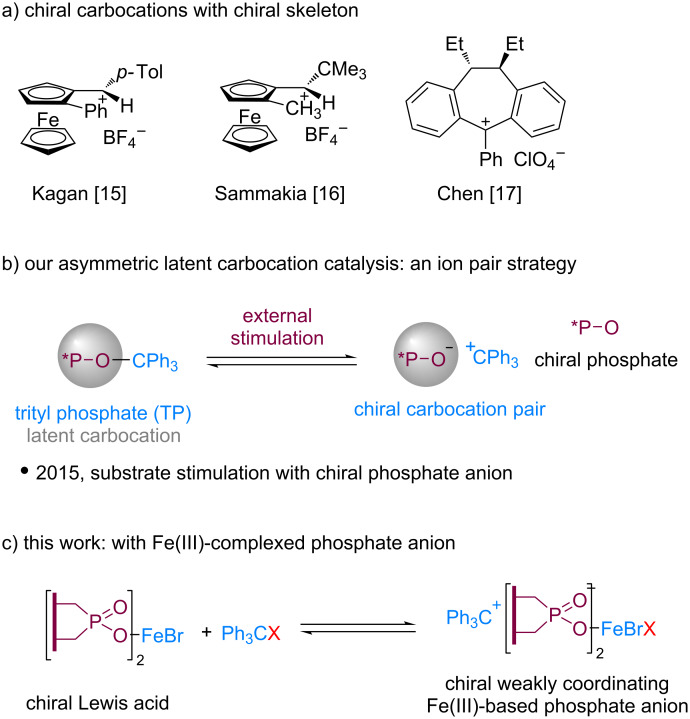
Asymmetric carbocation catalysis.

Carbocation Lewis acid catalysis has grown significantly over the last two decades [1–13]. The development of asymmetric carbocation catalysts has been long pursued but remains a challenging task. One strategy is to design and synthesize stabilized chiral carbocations with chirality installed onto their backbones. Pioneering efforts along this line by Kagan, Sammakia, and Chen have shown that chiral catalysis with such chiral carbocations was indeed plausible to achieve stereocontrol (Scheme 1a). [14–19]. However, the enantioselectivity was low in most cases. In addition, the synthetic efforts to access these chiral cations were generally non-trivial which limited their further development. Recently, we developed a chiral ion-pair strategy for asymmetric carbocation catalysis, with chiral trityl phosphate as the carbocation precursor [20–21]. In this latent strategy, the carbocation precursor can undergo facile ionic dissociation upon mild external stimulation such as polar substrates (such as α-ketoesters) to form a catalytically active chiral ion pair for substrate activation and chiral induction (Scheme 1b). In our further explorations, we noticed that the dissociation of trityl phosphate was generally sluggish, thus limiting its applicability. To expand its utility, we report herein a metal-complexed phosphate anion for chiral carbocation catalysis.

Weakly coordinating anions [22–23] have been widely used in inorganic and organic chemistry [24–27] as well as in polymer chemistry [28–33]. Although tritylium salts with various types of these counter anions based on B(III), Al(III), Ga(III), Fe(III), Nb(III), Ta(III), Y(III) and La(III) centers and ligands have been investigated in Lewis acid catalysis over the past decades, a chiral counter anion [34–35] with metal elements as the central atom, however, was seldom reported. Typically, the tritylium salts with weakly coordinating anions can be synthesized through a simple halide abstraction from the trityl halide in the presence of strong Lewis acids [36]. We herein report the design and exploration of a new trityl carbocation that has a chiral weakly coordinating Fe(III)-based phosphate anion for the effective asymmetric catalysis in the Diels–Alder reaction of anthracenes.

## Results and Discussion

**Scheme 2:**
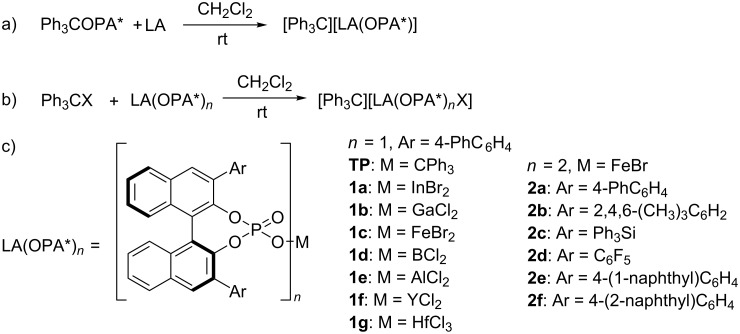
Synthesis of new carbocation catalysts with weakly coordinating metal-based phosphate anion.

**Figure 1:**
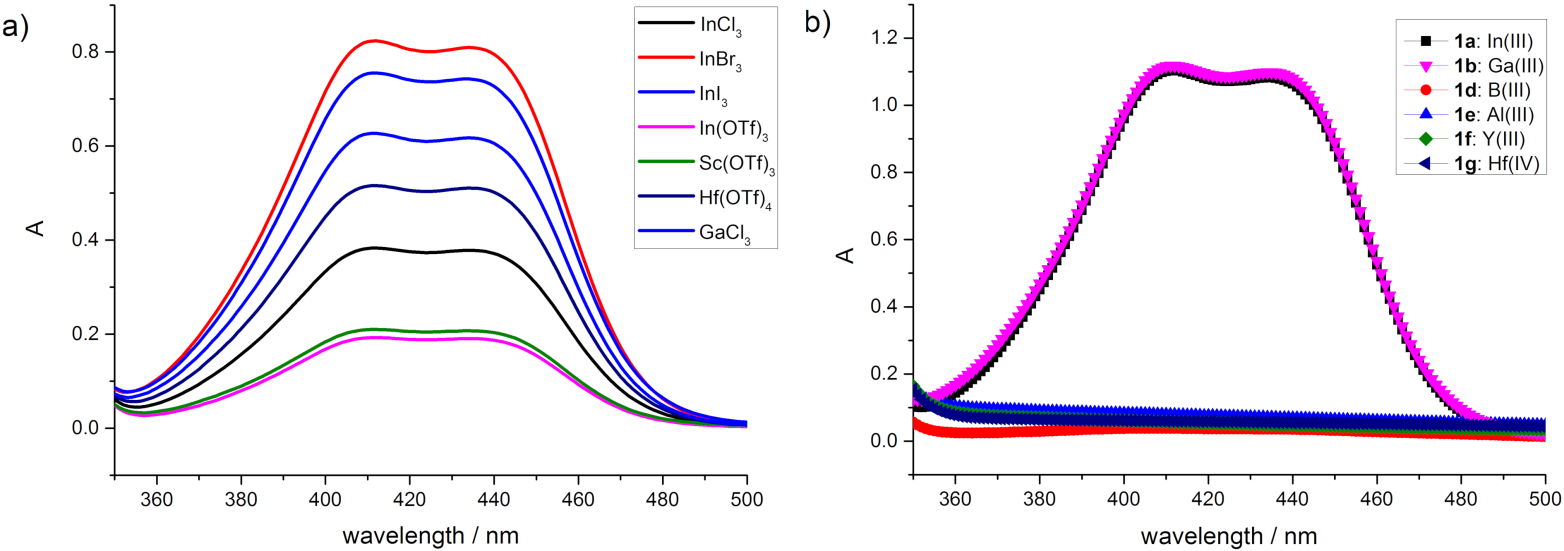
Dissociation of latent carbocation by the use of Lewis acids. a) UV–vis absorption spectra of **TP** (0.05 mM) upon the addition of Lewis acids (0.05 mM), such as InCl_3_, InBr_3_, InI_3_, In(OTf)_3_, Sc(OTf)_3_, Hf(OTf)_4_, and GaCl_3_. b) UV–vis absorption spectra of trityl bromide (Ph_3_CBr, 0.05 mM) upon the addition of the chiral Lewis acids (0.05 mM), such as **1a**, **b**, and **d**–**g**.

In our previous work, we found that less than 6% of trityl phosphate (TP) dissociated to trityl cations in the presence of a polar substrate such trifluoropyruvate [20]. In order to improve the efficiency of the dissociation, we started by first studying the properties of tritylium salts with a weakly coordinating metal-based phosphate anion (Scheme 2). Upon in situ mixing the chiral trityl phosphate (**TP**, 0.05 mM) and different Lewis acids (0.05 mM), such as InCl_3_, InBr_3_, InI_3_, In(OTf)_3_, Sc(OTf)_3_, Hf(OTf)_3_, GaCl_3_, and FeBr_3_, the originally colorless solution of the chiral trityl phosphate **TP** turned orange, suggesting the formation of tritylium ions (Scheme 2a). The stimulated trityl cation generation was probed by UV–vis spectroscopy. As shown in Figure 1a, when treated with different Lewis acids, trityl phosphate **TP** showed a variable tendency to dissociate into the free tritylium ion pair with InBr_3_ as the most active Lewis acid. An estimation based on UV absorption showed that approximately 76% of **TP** dissociated into trityl cations in the presence of InBr_3_. On the other hand, tritylium salts with a weakly coordinating metal-based monophosphate or bisphosphate anion could also be obtained when trityl bromide was treated with the corresponding metal phosphate, which can be prepared in situ following our previously described procedure (Scheme 2b,c) [37–38]. UV analysis indicated that the indium salt **1a** or gallium salt **1b** (0.05 mM) could induce ca. 92% dissociation of trityl bromide (0.05 mM) to generate the trityl cation. Also, FeBr_3_, a chiral Fe(III) monophosphate (M = FeBr_2_) **1c** or even the bulky Fe(III) bisphosphate **2a** promoted the dissociation of trityl bromide. In the latter case, the dissociation was estimated to be 54% by in-situ IR spectroscopy (UV–vis spectra were not applicable due to absorption overlap; see Supporting Information File 1 for details).

We next tested the metal phosphate strategy in the Diels–Alder reaction of anthracene, for which a catalytic asymmetric version has not been achieved yet. Recently, we reported that the tritylium salt [Ph_3_C][BArF], in situ generated by Ph_3_CBr and NaBArF, could promote the Diels–Alder reaction with anthracenes and various unsaturated carbonyl compounds under mild conditions [13]. The use of latent carbocation catalysis with **TP** was examined in order to achieve enantioselective control. To our delight, **TP** catalyzed the asymmetric reaction affording cycloadduct **5a** in excellent enantioselectivity (97% ee), however, with only 9% yield (Table 1, entry 1). Subsequent efforts to improve the activity by enhancing the dissociation efficiency of latent carbocation through heating or photolysis did not lead to any improvement. We next investigated whether the tritylium salts with a chiral weakly coordinating metal-based phosphate anion could facilitate the asymmetric catalytic Diels–Alder reaction. To implement this strategy, different trityl phosphates or halides, Lewis acids, chiral metal phosphate and their combinations were examined in the model reaction of anthracene (**3a**) and β,γ-unsaturated α-ketoester **4a**. When **TP** was first treated with metal Lewis acid (Scheme 2a, and Table S1 in Supporting Information File 1), the reaction showed good reactivity but no enantioselectivity at all, indicating a strong background reaction (Table 1, entry 2). We next examined the second strategy in which trityl bromide was treated with preformed chiral metal phosphate to their equilibration before they were subjected to the catalytic test. When metal monophosphates **1a–c** (Table 1, entries 3–5) were applied, the reaction started showing some enantioselectivity with decent activity maintained. The combined use of trityl bromide and **1a** (10 mol %) led to the desired adduct **5a** with 55% yield and in 14% ee at 50 °C (Table 1, entry 3). This is in contrast to the **TP**/InBr_3_ combination where the reaction was much faster but racemic (Table 1, entry 3 vs 2), suggesting that the preformed metal phosphate is critical to effect catalysis and chiral induction. Among the metals screened, Fe(III) phosphate gave the optimal results in terms of both activity and enantioselectivity (79% yield, 35% ee, Table 1, entry 5). Fe(III)-based bisphosphate anions were also tested. To our delight, when trityl bromide and **2a** (10 mol %) were used, the reaction gave a slightly increased enantioselectivity (Table 1, entry 6). Further improvement on activity and enantioselectivity could be achieved by conducting the reaction in DCM as the solvent (Table 1, entries 7 vs 6, 8–10). Next, we screened different chiral Fe(III) bisphosphates **2a**–**f** and the best results were obtained in the presence of **2f**, whereas others resulted in either low activity or poor enantioselectivity (Table 1, entries 15 vs 7, 11–14). Eventually, trityl chloride and chiral Fe(III) bisphosphate **2f** were identified to be the optimal combination, affording adduct **5a** in 91% ee and 70% yield at room temperature (Table 1, entries 17 and 18).

**Table 1:** Screening and optimization for the asymmetric catalyzed Diels–Alder reaction of anthracene by carbocations.

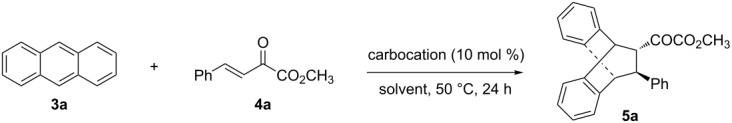					

entry^a^	carbocation		solvent	yield (%)^b^	ee^c^

	TrX	Lewis acid			

1	**TP**	none	DCE	9	97
metal-based monophosphate anion					
2	**TP**	InBr_3_	DCE	94	rac
3	Ph_3_CBr	**1a**	DCE	55	14
4	Ph_3_CBr	**1b**	DCE	49	−16
5	Ph_3_CBr	**1c**	DCE	79	36
Fe(III)-based bisphosphate anion					
6	Ph_3_CBr	**2a**	DCE	46	40
7	Ph_3_CBr	**2a**	DCM	58	56
8	Ph_3_CBr	**2a**	CHCl_3_	36	42
9	Ph_3_CBr	**2a**	toluene	20	46
10	Ph_3_CBr	**2a**	CH_3_CN	nr	–
11	Ph_3_CBr	**2b**	DCM	17	14
12	Ph_3_CBr	**2c**	DCM	55	28
13	Ph_3_CBr	**2d**	DCM	67	26
14	Ph_3_CBr	**2e**	DCM	22	68
15	Ph_3_CBr	**2f**	DCM	70	74
16^d^	Ph_3_CBr	**2f**	DCM	55	90
17^d^	Ph_3_CCl	**2f**	DCM	57	91
18^d,e^	Ph_3_CCl	**2f**	DCM	70	91
19^d^	none	**2f**	DCM	nr	–
20^d^	Ph_3_CCl	none	DCM	nr	–

^a^General conditions: **3a** (0.4 mmol), **4a** (0.2 mmol), TrX (10 mol %), and Lewis acid (10 mol %) in 2 mL solvent at 50 °C. ^b^Yield of isolated product. ^c^Determined by HPLC analysis on a chiral stationary phase. ^d^Room temperature. ^e^48 h.

In a control experiment, we found that chiral iron salt **2f** itself turned out to be ineffective to catalyze the reaction in the absence of trityl chloride (Table 1, entry 19), indicating that the reaction is catalyzed by tritylium salts with Fe(III)-complexed bisphosphate as the chirality-inducing anion.

With the optimal reactions conditions established, the scope was next explored with Ph_3_CCl/**2f** in CH_2_Cl_2_ (DCM) at room temperature and the results are presented in Table 2. A variety of β,γ-unsaturated α-ketoesters **4** was subjected to the reaction with anthracene (**3a**) to give the desired cycloadducts **5a**–**n** in moderate to good yields and with up to 93% ee. The bulkier isopropyl ketoester resulted in a lower yield and enantioselectivity (Table 2, entry 3 vs 1 and 2). Variations on the aromatic group of the ketoesters were well tolerated, giving the products in decent yields and high enantioselectivities. Unfortunately, no reaction was observed when an aliphatic substituted β,γ-unsaturated α-ketoester was used (data not shown).

**Table 2:** Scope for the asymmetric catalyzed Diels–Alder reaction of anthracene (**3a**) with ketoesters **4** by carbocations.

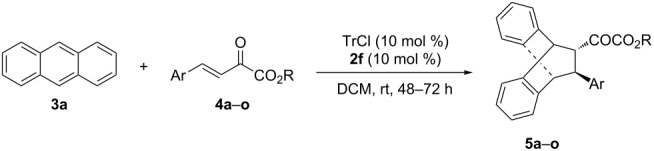				

entry^a^	α-ketoesters	product	yield (%)^b^	ee (%)^c^

1	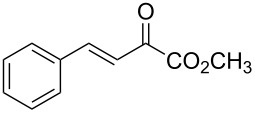 **4a**	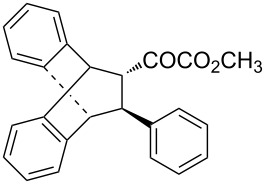 **5a**	70	91
2	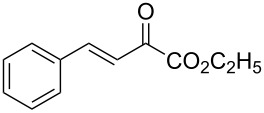 **4b**	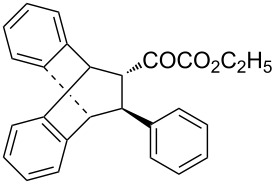 **5b**	82	74
3	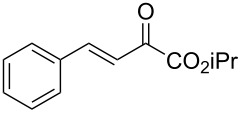 **4c**	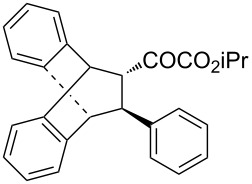 **5c**	46	55
4	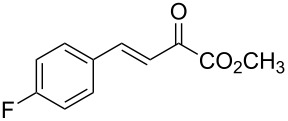 **4d**	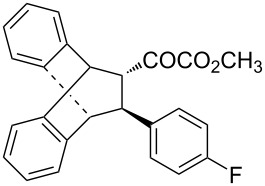 **5d**	74	80
5	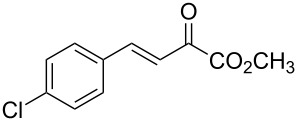 **4e**	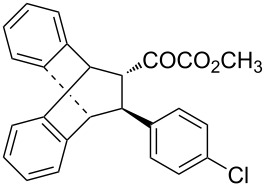 **5e**	68	75
6	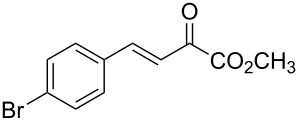 **4f**	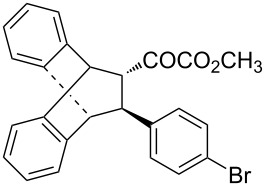 **5f**	66	81
7	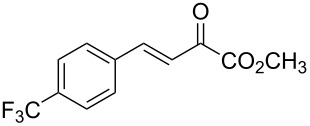 **4g**	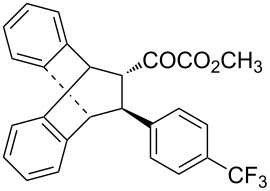 **5g**	77	76
8	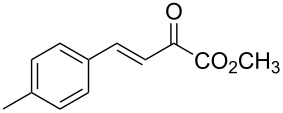 **4h**	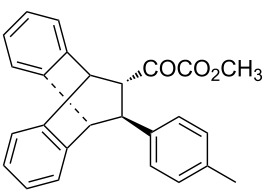 **5h**	48	80
9	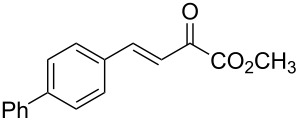 **4i**	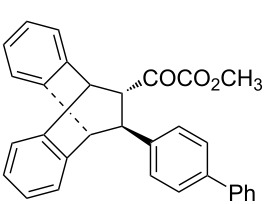 **5i**	68	93
10	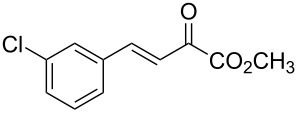 **4j**	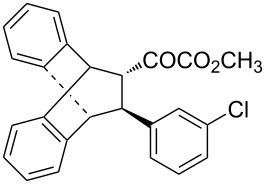 **5j**	92	91
11	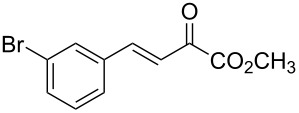 **4k**	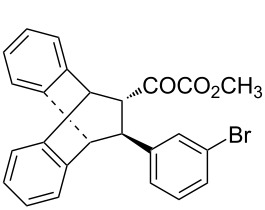 **5k**	86	87
12	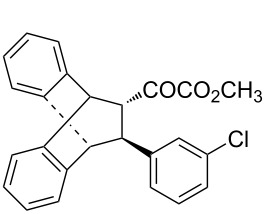 **4l**	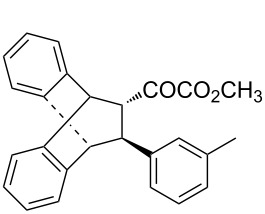 **5l**	76	89
13	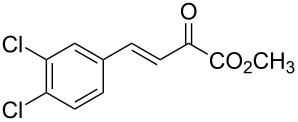 **4m**	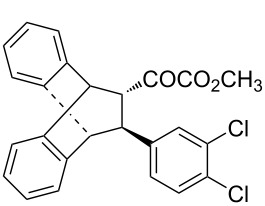 **5m**	85	73
14	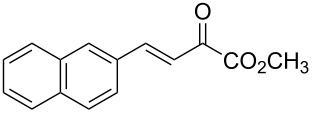 **4n**	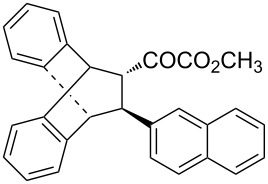 **5n**	42	83

^a^General conditions: **3a** (0.4 mmol), **4** (0.2 mmol), TrCl (10 mol %), and **2a** (10 mol %) in DCM (2 mL) at room temperature. ^b^Yield of isolated product. ^c^Determined by HPLC analysis on a chiral stationary phase.

**Scheme 3:**
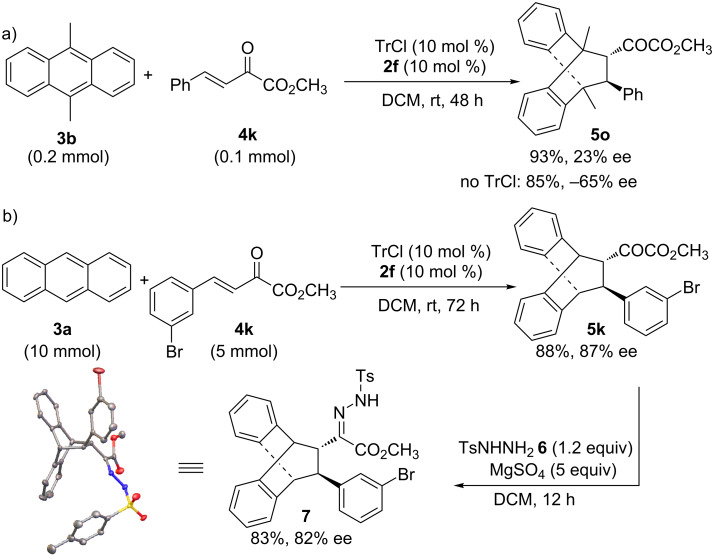
a) The reaction with 9,10-dimethylanthracene (**3b**). b) Gram-scale reaction of **3a** and **4k**, and transformation of cycloadduct **5k**.

The Diels–Alder reaction of substituted anthracenes has been well-developed and we next examined the scope with substituted anthracenes. Unfortunately, these well-explored substrates did not work in our chiral catalysis system giving either no activity or poor enantioselectivity, particularly in cases of 9-monosubstituted anthracenes. When 9,10-dimethylanthracene (**3b**) was used, the reaction showed high yield (93% for **5o**) but low enantioselectivity (23% ee, Scheme 3a). Surprisingly, the chiral iron salt **2f** itself in the absence of trityl chloride also promoted the reaction, showing a relatively lower activity with 85% yield of **5o** but opposite chiral induction (−65% ee, Scheme 3a). The electron-rich nature of dimethylanthracene may account for catalysis with the iron salts. On the other hand, an opposite chiral induction in this case is a clear indication of distinctive carbocation catalysis instead of metal Lewis acid catalysis in the presence of trityl chloride.

In addition, we tested the current carbocation catalytic system to prepare cycloadduct **5k** in a large scale (Scheme 3b). When using 10 mol % Ph_3_CCl/**2f**, the reaction afforded cycloadduct **5k** in 88% yield of isolated product and with 87% ee. In the presence of MgSO_4_ (5 equiv), treatment of **5k** (1 equiv) with sulfonylhydrazine **6** (1.2 equiv) in CH_2_Cl_2_ led to the desired *N*-tosylhydrazone **7** in 83% yield and with 82% ee (Scheme 3b). The absolute configuration was assigned on the basis of the structure of **7**, which was confirmed unambiguously by an X-ray crystallographic study [39]. Tentative transition states to account for the observed stereoselectivity are provided in Supporting Information File 1, Figure S3.

## Conclusion

In summary, we have introduced a new motif of chiral weakly coordinating Fe(III)-based bisphosphate anion for high performance asymmetric carbocation Lewis acid catalysis. The introduction of a metal-coordinated phosphonate anion with balanced association ability with tritylium ions provided a new opportunity in pursuing chiral ion pair-type carbocation catalysis. The resulted asymmetric tritylium catalysis has enabled the so-far challenging Diels–Alder reactions of unsubstituted anthracene with good activity and up to 93% ee. Further studies are currently underway to elucidate the mechanistic details and to extend the chiral tritylium salt catalysis to other reactions.

## Supporting Information

File 1 Experimental procedures and characterization data of all products, copies of ^1^H and ^13^C NMR, IR, HRMS, and HPLC spectra of all compounds.
