# Supplementary material for: Enantioselective Diels–Alder reaction of anthracene by chiral tritylium catalysis
Source: Beilstein J Org Chem. 2019 Jun 14;15:1304–12. doi: 10.3762/bjoc.15.129 (PMC6604712; doi:10.3762/bjoc.15.129)
Supplement: File 1 — Experimental procedures and characterization data of all products, copies of 1H and 13C NMR, IR, HRMS, and HPLC spectra of all compounds. [file Beilstein_J_Org_Chem-15-1304-s001.pdf]

BEILSTEIN JOURNAL OF ORGANIC CHEMISTRY

Supporting Information

for

Enantioselective Diels–Alder reaction of anthracene by chiral tritylium catalysis

Qichao Zhang, Jian Lv and Sanzhong Luo

Beilstein J. Org. Chem. **2019,** *15,* 1304–1312. doi:10.3762/bjoc.15.129

Experimental procedures and characterization data of all products, copies of ^{1}H and ^{13}C NMR, IR, HRMS, and HPLC spectra of all compounds

License and Terms: This is a supporting information file under the terms of the Creative Commons Attribution License (http://creativecommons.org/licenses/by/4.0). Please note that the reuse, redistribution and reproduction in particular requires that the authors and source are credited.
The license is subject to the *Beilstein Journal of Organic Chemistry* terms and conditions: (https://www.beilstein-journals.org/bjoc)

Table of contents

1. General information and materials..........S2

2. Experimental section..........S3

3. Characterization..........S7

3. ^{1}H NMR and ^{13}C NMR spectra..........S19

4. HPLC spectra..........S35

General information and materials

Commercial reagents were used as received, unless other indicated. ^{1}H and ^{13}C NMR spectra were measured on a NMR instrument (400 and 500 MHz for ^{1}H NMR, 100 and 125 MHz for ^{13}C NMR). Tetramethylsilane (TMS) served as the internal standard for ^{1}H NMR, and $CDCl_3$ served as the internal standard for ^{13}C NMR. The following abbreviations were used to express the multiplicities: s = singlet; d = doublet; t = triplet; q = quartet; m = multiplet; br = broad. Silica gel (200–300 mesh) was used for column chromatography. The enantioselective excesses (ee) were determined by HPLC analysis on Chiral Daicel Chiralcel AD-H, OD-H, AS-H and OJ-H columns. Optical rotation were measured on a commercial polarimeter and are reported as follows: $[\alpha]_D^{25}$ (c = g/100 mL, solvent). HRMS were recorded on a commercial apparatus (ESI Source).

Experimental section

Dissociation of latent carbocation by using Lewis acids

General procedure I: TP (0.005 mmol) was dissolved in dry CH_2Cl_2 (1 mL). The corresponding Lewis acid (0.005 mmol), such as $InCl_3$, $InBr_3$, InI_3, $In(OTf)_3$, $Sc(OTf)_3$, $Hf(OTf)_4$, and $GaCl_3$, was added, respectively. After each addition, the solution was stirred for 10 min before UV–vis testing was performed (see Figure 1a).

General procedure II: Ph_3CBr (0.005 mmol) was dissolved in dry CH_2Cl_2 (1 mL) and the corresponding chiral Lewis acid (0.005 mmol) was added. After each addition, the solution was stirred for 30 min before UV–vis testing was performed (see Figure 1b).

General procedure III: To an oven-dried reaction tube was added the corresponding Lewis acid (0.05 mmol), and distilled anhydrous CH_2Cl_2 (0.8 mL) was added. Then, a solution of trityl bromide in CH_2Cl_2 (0.2 mL) was added and the reaction mixture stirred at room temperature. In situ IR spectroscopy was used to monitor the reaction progress (at 1584 cm^{-1}, Figure S1) as well as for monitoring of the conversion of the free carbocation (Figure S2).

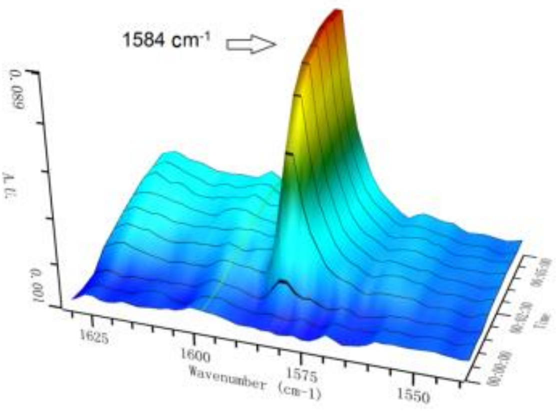

Figure S1: Representative 3D stacking plots of in situ IR spectra of trityl cation generation.

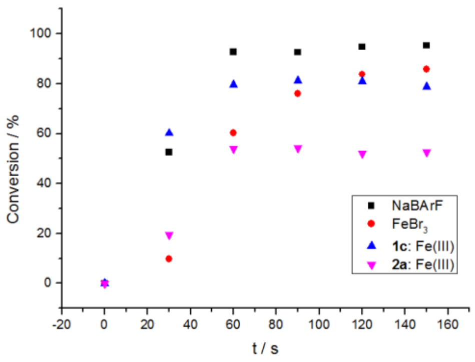

Figure S2: Dissociation progress of Ph3CBr (0.05 mmol) in the presence of NaBArF, FeBr3, **1c**, or **2a** (0.05 mmol), monitored by in situ IR at 1584 cm^{-1}.

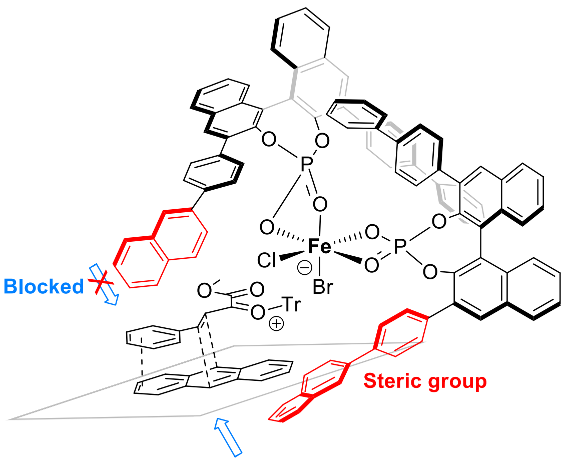

Figure S3: Proposed transition-state mode.

General procedure for chiral carbocation catalyzed asymmetric Diels–Alder reactions between anthracene and β,γ-unsaturated-α-keto esters: To dichloromethane was added $FeBr_3$ (0.02 mmol, 10 mol %) and silver salt of chiral phosphoric acid (0.04 mmol, 20 mol %). After stirring for 3 h, Ph_3CCl (0.02 mmol, 10 mol %) was added and the reaction mixture was stirred for additional 3 h at rt. Then, β,γ-unsaturated-*α*-keto esters **4** (0.2 mmol) and anthracene (**3**, 0.4 mmol) were added and the reaction mixture was stirred for up to 3 d at rt. Purification by column chromatography on silica gel with petroleum ether/dichloromethane 2:1 afforded the desired products **5**.

Table S1: Optimization studies for asymmetric catalyzed Diels–Alder reaction of anthracene by **TP** and different Lewis acids.

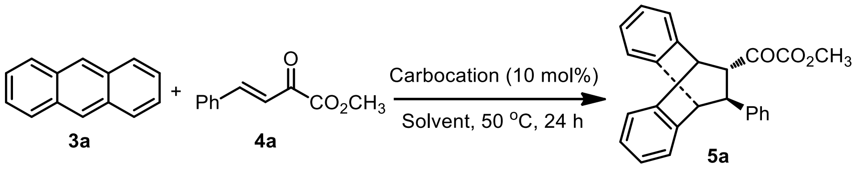

entry[a]	carbocation TrX	Lewis acid	solvent	yield (%)[b]	ee[c]
1	**TP**	None	DCE	9	97
2	**TP**	$InCl_3$	DCE	62	9
3	**TP**	$InBr_3$	DCE	94	Rac.
4	**TP**	InI_3	DCE	42	36
5	**TP**	$In(OTf)_3$	DCE	89	Rac.
6	**TP**	$Hf(OTf)_4$	DCE	90	Rac.
7	**TP**	$Sc(OTf)_3$	DCE	86	Rac.
8	**TP**	$GaCl_3$	DCE	81	-21

[a] General condition: **3a** (0.4 mmol), **4a** (0.2 mmol), **TP** (10 mol%), and Lewis acid (10 mol%) in the reaction solvent (2 mL) at 50 °C.

Characterization

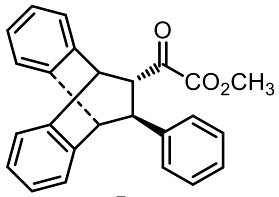

5a

5a: Colorless solid; 70% yield, 91% *ee*; $[\alpha]_D^{22}$ = +25.5 (c = 1.04); ^{1}H NMR (500 MHz, $CDCl_3$) δ 7.47 (d, *J* = 7.5 Hz, 1H), 7.33 (d, *J* = 7.5 Hz, 1H), 7.25-7.09 (m, 8H), 7.03 (d, *J* = 7.0 Hz, 1H), 6.57 (d, *J* = 5.5 Hz, 2H), 4.80 (s, 1H), 4.25 (s, 1H), 3.80 (s, 3H), 3.73 (dd, *J* = 6.0 Hz, 2.0 Hz, 1H), 3.63 (dd, *J* = 6.0 Hz, 2.0 Hz, 1H) ppm; ^{13}C NMR (100 MHz, $CDCl_3$) δ 192.6, 162.1, 144.1, 142.5, 142.5, 140.9, 138.5, 128.3, 128.1, 126.9, 126.9, 126.6, 126.5, 126.4, 126.3, 125.1, 123.5, 123.4, 56.8, 53.0, 51.9, 46.9, 46.8 ppm; HPLC analysis: Daicel Chiralpak AS-H, hexane/iso-propanol = 97:3, flow rate = 1.0 mL/min, λ = 206 nm, retention time: 9.5 min (major) and 13.1 min (minor); IR (KBr, cm^{-1}): 3025, 2952, 1732, 1601, 1493, 1467, 1458, 1259, 1205, 1152, 1073, 760, 702, 640, 587, 567; HRMS (ESI) calcd for $C_{27}H_{24}O_3Na^+$: 391.1305, found: 391.1304.

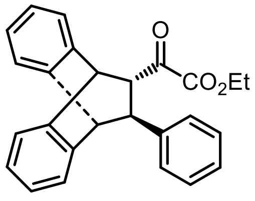

5b

5b: Colorless solid; 82% yield, 74% *ee*; $[\alpha]_D^{22}$ = +22.0 (c = 1.06); ^{1}H NMR (400 MHz, $CDCl_3$) δ 7.44 (d, *J* = 7.2 Hz, 1H), 7.30 (d, *J* = 6.8 Hz, 1H), 7.23–7.06 (m, 8H), 7.00 (d, *J* = 7.2 Hz, 1H), 6.57 (dd, *J* = 5.2 Hz, 1.6 Hz, 2H), 4.81 (d, *J* = 2.0 Hz, 1H), 4.24-4.15 (m, 3H), 3.71 (dd, *J* = 6.0 Hz, 2.0 Hz, 1H), 3.62 (dd, *J* = 6.0 Hz, 2.0 Hz, 1H), 1.21 (t, *J*

= 7.2 Hz, 3H) ppm; ^{13}C NMR (100 MHz, $CDCl_3$) δ 193.0, 161.7, 144.0, 142.5, 140.9, 138.6, 128.3, 128.0, 126.8, 126.7, 126.5, 126.5, 126.3, 126.2 125.1, 123.4, 123.3, 62.5, 56.7, 51.9, 46.7, 46.7, 13.9 ppm; HPLC analysis: Daicel Chiralpak AS-H, hexane/iso-propanol = 97:3, flow rate = 1.0 mL/min, λ = 206 nm, retention time: 8.1 min (major) and 13.2 min (minor); IR (KBr, cm^{-1}): 3067, 3025, 2982, 2949, 1726, 1467, 1458, 1298, 1256, 1072, 1033, 1019, 760, 702; HRMS (ESI) calcd for $C_{26}H_{22}O_3Na^+$: 405.1461, found: 405.1459.

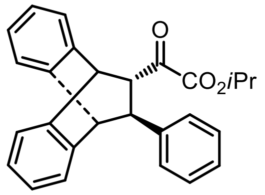

5c

5c: Colorless solid; 46% yield, 55% *ee*; $[\alpha]_D^{22}$ = +11.3 (c = 0.98); ^{1}H NMR (400 MHz, $CDCl_3$) δ 7.44 (d, *J* = 7.2 Hz, 1H), 7.30 (d, *J* = 6.8 Hz, 1H), 7.23-7.06 (m, 8H), 7.00 (d, *J* = 7.2 Hz, 1H), 6.59-6.57 (m, 2H), 5.10-5.04 (m, 1H), 4.79 (s, 1H), 4.23 (d, *J* = 1.6 Hz, 1H), 3.68 (dd, *J* = 6.0, 2.0 Hz, 1H), 3.62 (dd, *J* = 6.0, 2.0 Hz, 1H), 1.24 (d, *J* = 6.4 Hz, 3H), 1.21 (d, *J* = 6.4 Hz, 3H) ppm; ^{13}C NMR (100 MHz, $CDCl_3$) δ 193.4, 161.5, 144.0, 142.6, 142.5, 140.9, 138.7, 128.3, 128.0, 126.8, 126.7, 126.5, 126.5, 126.3, 126.2, 125.2, 123.4, 123.3 70.8, 56.6, 52.0, 46.7, 46.7, 21.6, 21.6 ppm; HPLC analysis: Daicel Chiralpak AS-H, hexane/iso-propanol = 97:3, flow rate = 1.0 mL/min, λ = 206 nm, retention time: 7.2 min (major) and 11.8 min (minor); IR (KBr, cm^{-1}): 3067, 3025, 2982, 2939, 1722, 1467, 1458, 1259, 1082, 1071, 761, 702; HRMS (ESI) calcd for $C_{27}H_{25}O_3^+$: 397.1798, found: 397.1794.

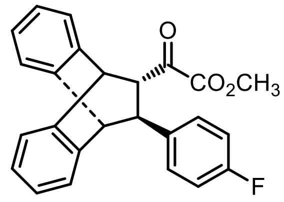

5d

5d: Colorless solid; 74% yield, 80% *ee*; $[\alpha]_D^{22}$ = +24.7 (c = 1.16); ^{1}H NMR (500 MHz, $CDCl_3$) δ 7.47 (d, *J* = 7.0 Hz, 1H), 7.20 (d, *J* = 7.0 Hz, 1H), 7.24–7.08 (m, 5H), 7.02 (d, *J* = 7.0 Hz, 1H), 6.80 (t, *J* = 8.5 Hz, 2H), 6.51 (dd, *J* = 8.5, 5.5 Hz, 2H), 4.80 (s, 1H), 4.21 (s, 1H), 3.80 (s, 3H), 3.67-3.62 (m, 2H) ppm; ^{13}C NMR (125 MHz, $CDCl_3$) δ 192.3, 161.9, 161.7 (d, *J* = 243.8 Hz), 143.7, 142.2, 140.6, 138.3, 138.1, 129.3 (d, *J* = 6.6 Hz), 126.8, 126.5, 126.4, 126.4, 126.2, 125.0, 123.5, 123.3, 115.0 (d, *J* = 21.0 Hz), 56.8, 53.0, 51.8, 46.7, 45.8 ppm; HPLC analysis: Daicel Chiralpak AD-H (2x), hexane/iso-propanol = 97:3, flow rate = 0.6 mL/min, λ = 230 nm, retention time: 31.7 min (minor) and 36.0 min (major); IR (KBr, cm^{-1}): 3069, 3044, 3024, 2953, 1733, 1603, 1511, 1458, 1259, 1226, 1170, 1078, 837, 801. 759, 703; HRMS (ESI) calcd for $C_{25}H_{19}O_3FNa^+$: 409.1210, found: 409.1210.

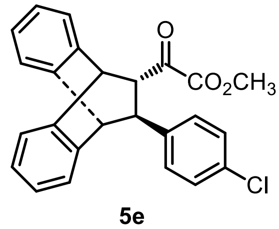

5e

5e: Colorless solid; 68% yield, 75% *ee*; $[\alpha]_D^{22}$ = +36.2 (c = 1.01); ^{1}H NMR (400 MHz, $CDCl_3$) δ 7.44 (d, *J* = 7.2 Hz, 1H), 7.30 (d, *J* = 7.2 Hz, 1H), 7.20-7.04 (m, 7H), 6.99 (d, *J* = 7.2 Hz, 1H), 6.46 (d, *J* = 8.4 Hz, 2H), 4.81 (d, *J* = 1.6 Hz, 1H), 4.20 (d, *J* = 1.6 Hz,

1H), 3.73 (s, 3H), 3.66 (dd, *J* = 5.6, 2.0 Hz, 1H), 6.62 (dd, *J* = 5.6, 2.0 Hz, 1H) ppm; ^{13}C NMR (100 MHz, $CDCl_3$) δ 192.3, 161.9, 143.6, 142.2, 140.9, 140.5, 138.3, 132.6, 129.3, 128.3, 126.8, 126.6, 126.4, 126.4, 126.3, 125.0, 123.5, 123.4, 56.7, 52.9, 51.6, 46.6, 46.0; HPLC analysis: Daicel Chiralpak AD-H (2x), hexane/iso-propanol = 97:3, flow rate = 0.6 mL/min, λ = 210 nm, retention time: 36.0 min (minor) and 40.5 min (major); IR (KBr, cm^{-1}): 3069, 3043, 3024, 2952, 1732, 1492, 1458, 1265, 1078, 1013, 830, 759, 721; HRMS (ESI) calcd for $C_{25}H_{19}O_3ClNa^+$: 425.0915, found: 425.0915.

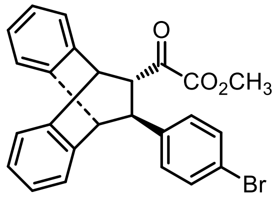

5f

5f: Colorless solid; 66% yield, 81% *ee*; $[\alpha]_D^{22}$ = +46.8 (c = 1.02); ^{1}H NMR (400 MHz, $CDCl_3$) δ 7.47 (d, *J* = 7.5 Hz, 1H), 7.32 (d, *J* = 7.0 Hz, 1H), 7.24 (d, *J* = 9.0 Hz, 3H), 7.22-7.09 (m, 4H), 7.02 (d, *J* = 7.0 Hz, 1H), 6.42 (d, *J* = 8.5 Hz, 2H), 4.80 (s, 1H), 4.20 (s, 1H), 3.83 (s, 3H), 3.65 (dd, *J* = 6.0, 2.0 Hz, 1H), 3.60 (dd, *J* = 5.5, 1.5 Hz, 1H); ^{13}C NMR (100 MHz, $CDCl_3$) δ 192.3, 161.9, 143.6, 142.3, 141.5, 140.5, 138.4, 131.4, 129.7, 128.0, 126.9, 126.7, 126.5, 126.5, 126.4 125.1, 123.6, 123.4, 120.8, 56.8, 53.1, 51.6, 46.7, 46.2; HPLC analysis: Daicel Chiralpak AD-H (2x), hexane/iso-propanol = 97:3, flow rate = 0.6 mL/min, λ = 254 nm, retention time: 39.0 min (minor) and 42.1 min (major); IR (KBr, cm^{-1}): 3032, 3023, 2951, 1729, 1489, 1458, 1264, 1076, 1009, 816, 759; HRMS (ESI) calcd for $C_{25}H_{19}O_3BrNa^+$: 469.0410, found: 469.0409.

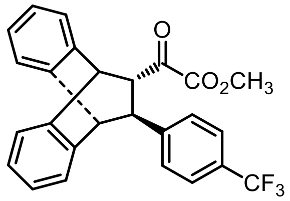

5g

5g: Colorless solid; 77% yield, 76% *ee*; $[\alpha]_D^{22}$ = +25.6 (c = 1.14); ^{1}H NMR (500 MHz, $CDCl_3$) δ 7.49 (d, *J* = 7.5 Hz, 1H), 7.38 (d, *J* = 8.0 Hz, 2H), 7.34 (d, *J* = 7.0 Hz, 1H), 7.27–7.11 (m, 5H), 7.02 (d, *J* = 7.5 Hz, 1H), 6.67 (d, *J* = 8.0 Hz, 2H), 4.83 (s, 1H), 4.24 (d, 1H), 3.84 (s, 3H), 3.71 (s, 2H) ppm; ^{13}C NMR (125 MHz, $CDCl_3$) δ 192.1, 161.9, 146.6, 143.5, 142.3, 140.4, 138.4, 129.2 (q, *J* = 32.3 Hz), 128.4, 127.0, 126.8, 126.6, 126.6, 126.5, 125.3 (q, *J* = 3.9 Hz), 125.2, 124.2 (q, *J* = 270.5 Hz), 123.7, 123.5, 56.7, 53.1, 51.5, 46.8, 46.5 ppm; HPLC analysis: Daicel Chiralpak AD-H (2x), hexane/iso-propanol = 97:3, flow rate = 0.6 mL/min, λ = 206 nm, retention time: 31.2 min (minor) and 35.1 min (major); IR (KBr, cm^{-1}): 3071, 3044, 3025, 2954, 1732, 1618, 1458, 1326, 1259, 1166, 1114, 1069, 1017, 842, 759, 660; HRMS (ESI) calcd for $C_{26}H_{18}O_3F_3^-$: 435.1214, found: 435.1213.

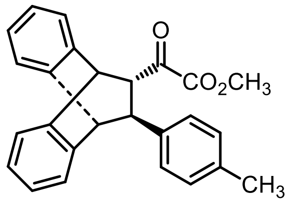

5h

5h: Colorless solid; 48% yield, 80% *ee*; $[\alpha]_D^{22}$ = +46.8 (c = 0.92); ^{1}H NMR (400 MHz, $CDCl_3$) δ 7.46 (d, *J* = 7.0 Hz, 1H), 7.32 (d, *J* = 7.5 Hz, 1H), 7.25–7.08 (m, 5H), 7.04 (d, *J* = 7.0 Hz, 1H), 6.93 (d, *J* = 8.0 Hz, 2H), 6.46 (d, *J* = 8.0 Hz, 2H), 4.79 (d, *J* = 1.0 Hz, 1H), 4.22 (d, *J* = 1.5 Hz, 1H), 3.80 (s, 3H), 3.70 (dd, *J* = 6.0, 1.5 Hz, 1H), 3.59 (dd, *J* =

5.5, 1.5 Hz, 1H), 2.25 (s, 3H) ppm; ^{13}C NMR (100 MHz, $CDCl_3$) δ 192.6, 162.0, 144.1, 142.4, 140.9, 139.4, 138.5, 136.3, 128.9, 127.8, 126.7, 126.5, 126.4, 126.3, 126.1, 125.0, 123.4, 123.3, 56.8, 52.8, 51.9, 46.8, 46.3, 21.0 ppm; HPLC analysis: Daicel Chiralpak AS-H, hexane/iso-propanol = 97:3, flow rate = 1.0 mL/min, λ = 206 nm, retention time: 8.6 min (minor) and 12.3 min (major); IR (KBr, cm^{-1}): 3023, 2951, 1733, 1514, 1467, 1078, 813, 758, 722; HRMS (ESI) calcd for $C_{26}H_{22}O_3Na^+$: 405.1460, found: 405.1461.

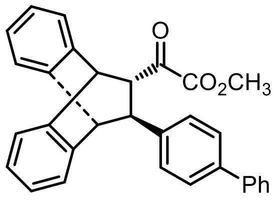

5i

5i: Colorless solid; 68% yield, 93% *ee*; $[\alpha]_D^{22}$ = +60.5 (c = 1.02); ^{1}H NMR (400 MHz, $CDCl_3$) δ 7.46-6.98 (m, 15H), 6.62 (d, *J* = 8.4 Hz, 2H), 4.83 (d, *J* = 1.6 Hz, 1H), 4.26 (d, *J* = 1.6 Hz, 1H), 3.77 (dd, *J* = 6.0 Hz, 2.0 Hz, 1H), 3.71 (dd, *J* = 5.6 Hz, 1.6 Hz, 1H), 3.61 (s, 3H) ppm; ^{13}C NMR (100 MHz, $CDCl_3$) δ 192.4, 161.9, 143.9, 142.3, 141.4, 140.7, 140.4, 139.5, 138.4, 128.7, 128.3, 127.2, 126.8, 126.7, 126.4, 126.4, 126.3, 126.1, 125.0, 123.4, 123.3, 56.7, 52.7, 51.7, 46.7, 46.3 ppm; HPLC analysis: Daicel Chiralpak AS-H, hexane/iso-propanol = 97:3, flow rate = 1.0 mL/min, λ = 254 nm, retention time: 13.4 min (major) and 28.8 min (minor); IR (KBr, cm^{-1}): 3026, 2951, 1733, 1486, 1458, 1265, 1216, 1078, 1007, 840, 764, 698, 634; HRMS (ESI) calcd for $C_{31}H_{24}O_3Na^+$: 467.1618, found: 467.1618.

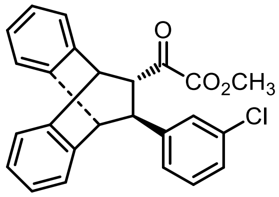

5j

5j: Colorless solid; 92% yield, 91% *ee*; $[\alpha]_D^{22}$ = +31.9 (c = 1.11); ^{1}H NMR (500 MHz, $CDCl_3$) δ 7.47 (d, *J* = 7.0 Hz, 1H), 7.32 (d, *J* = 7.00 Hz, 1H), 7.24–7.00 (m, 8H), 6.54 (s, 1H), 6.41 (d, *J* = 7.5 Hz, 1H), 4.81 (d, *J* = 1.5 Hz, 1H), 4.23 (d, *J* = 1.5 Hz, 1H), 3.80 (s, 3H), 3.66 (dd, *J* = 5.5, 1.5 Hz, 1H), 3.62 (dd, *J* = 5.5, 2.0 Hz, 1H) ppm; ^{13}C NMR (125 MHz, $CDCl_3$) δ 192.2, 161.9, 144.6, 143.6, 142.2, 140.5, 138.4, 134.1, 129.5, 128.3, 127.0, 126.9, 126.7, 126.5, 126.5, 126.4, 126.1, 125.1, 123.7, 123.5, 56.7, 53.1, 51.6, 46.7, 46.3 ppm; HPLC analysis: Daicel Chiralpak AD-H (2x), hexane/iso-propanol = 97:3, flow rate = 0.6 mL/min, λ = 230 nm, retention time: 30.4 min (minor) and 34.9 min (major); IR (KBr, cm^{-1}): 3069, 3043, 3024, 2952, 1732, 1595, 1467, 1264, 1077, 783, 760, 701; HRMS (ESI) calcd for $C_{25}H_{18}O_3Cl^-$: 401.0950, found: 401.0948.

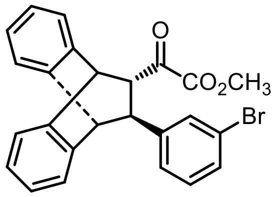

5k

5k: Colorless solid; 86% yield, 87% *ee*; $[\alpha]_D^{22}$ = +26.7 (c = 1.04); ^{1}H NMR (400 MHz, $CDCl_3$) δ 7.47 (d, *J* = 7.2 Hz, 1H), 7.31 (d, *J* = 7.2 Hz, 1H), 7.26-7.07 (m, 6H), 7.02 (d, *J* = 7.2 Hz, 1H), 6.95 (t, *J* = 7.6 Hz, 1H), 6.70 (s, 1H), 6.43 (d, *J* = 7.6 Hz, 1H), 4.81 (d, *J* = 1.6 Hz, 1H), 4.23 (d, *J* = 2.0 Hz, 1H), 3.79 (s, 3H), 3.66 (dd, *J* = 6.0, 2.0 Hz, 1H), 3.61 (dd, *J* = 6.0, 2.0 Hz, 1H) ppm; ^{13}C NMR (100 MHz, $CDCl_3$) δ 192.2, 161.9, 144.8,

143.5, 142.2, 140.4, 138.3, 131.3, 129.9, 129.8, 126.9, 126.7, 126.5, 126.5, 126.4, 125.1, 123.6, 123.5, 122.3, 56.7, 53.1, 51.5, 46.7, 46.3 ppm; HPLC analysis: Daicel Chiralpak AD-H (2x), hexane/iso-propanol = 97:3, flow rate = 0.6 mL/min, λ = 230 nm, retention time: 30.6 min (minor) and 38.4 min (major); IR (KBr, cm^{-1}):3068, 3043, 3023, 2952, 1732, 1592, 1566, 1467, 1458, 1432, 1264, 1076, 782, 759, 698; HRMS (ESI) calcd for $C_{25}H_{19}O_3BrNa^+$: 469.0410, found: 469.0411.

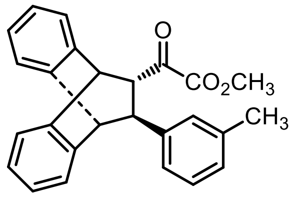

5l

5l: Colorless solid; 76% yield, 89% *ee*; $[\alpha]_D^{22}$ = +25.0 (c = 1.15); ^{1}H NMR (500 MHz, $CDCl_3$) δ 7.47 (d, *J* = 7.0 Hz, 1H), 7.32 (d, *J* = 7.5 Hz, 1H), 7.25-6.94 (m, 8H), 6.39 (s, 1H), 6.32 (d, *J* = 7.5 Hz, 1H), 4.80 (s, 1H), 4.24 (s, 1H), 3.81 (s, 3H), 3.72 (dd, *J* = 6.0, 2.0 Hz, 1H), 3.59 (dd, *J* = 6.0, 2.0 Hz, 1H), 2.18 (s, 3H) ppm; ^{13}C NMR (125 MHz, $CDCl_3$) δ 192.6, 162.1, 144.1, 142.4, 141.0, 138.5, 137.8, 129.1, 128.2, 128.0, 127.5, 126.8, 126.6, 126.5, 126.3, 126.2, 125.1, 124.9, 123.5, 123.4, 56.8, 53.0, 51.9, 46.9, 46.6, 21.5 ppm; HPLC analysis: Daicel Chiralpak AD-H (2x), hexane/iso-propanol = 97:3, flow rate = 0.6 mL/min, λ = 206 nm, retention time: 25.9 min (minor) and 30.5 min (major); IR (KBr, cm^{-1}): 3042, 3023, 2951, 1729, 1467, 1458, 1262, 1078,785, 758, 707; HRMS (ESI) calcd for $C_{26}H_{21}O_3^-$: 381.1496, found: 381.1495.

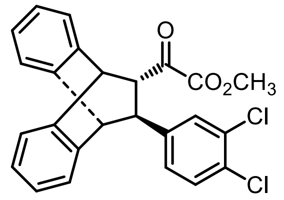

5m

5m: Colorless solid; 85% yield, 73% *ee*; $[\alpha]_D^{22}$ = +46.8 (c = 1.05); ^{1}H NMR (500 MHz, $CDCl_3$) δ 7.48 (d, *J* = 7.5 Hz, 1H), 7.32 (d, *J* = 7.0 Hz, 1H), 7.25–7.09 (m, 6H), 7.02 (d, *J* = 7.0 Hz, 1H), 6.63 (d, *J* = 2.0 Hz, 1H), 6.33 (dd, *J* = 8.0 Hz, 1.5 Hz, 1H), 4.82 (s, 1H), 4.21 (s, 1H), 3.83 (s, 3H), 3.62 (s, 2H); ^{13}C NMR (125 MHz, $CDCl_3$) δ 192.0, 161.9, 143.3, 142.8, 142.1, 140.2, 138.3, 132.2, 130.9, 130.2, 130.2, 127.3, 127.0, 126.9, 126.6, 126.6, 126.5, 125.1, 123.7, 123.5, 56.8, 53.2, 51.5, 46.7, 45.8; HPLC analysis: Daicel Chiralpak AD-H (2x), hexane/iso-propanol = 97:3, flow rate = 0.6 mL/min, λ = 230 nm, retention time: 33.0 min (minor) and 41.0 min (major); IR (KBr, cm^{-1}): 3069, 3043, 3024, 2952, 1729, 1468, 1264, 1080, 1029, 758, 709; HRMS (ESI) calcd for $C_{25}H_{17}O_3Cl_2^-$: 435.0560, found: 435.0564.

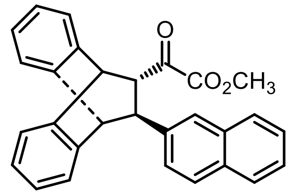

5n

5n: Colorless solid; 42% yield, 83% *ee*; $[\alpha]_D^{22}$ = +47.4 (c = 0.97); ^{1}H NMR (500 MHz, $CDCl_3$) δ 7.72-7.71 (m, 1H), 7.59-7.57 (m, 2H), 7.51 (d, *J* = 7.5 Hz, 1H), 7.39-7.34 (m, 3H), 7.27–7.07 (m, 7H), 6.98 (d, *J* = 7.5 Hz, 1H), 6.61 (dd, *J* = 8.5 Hz, 1.5 Hz, 1H), 4.86 (d, *J* = 1.5 Hz, 1H), 4.32 (d, *J* = 1.5 Hz, 1H), 3.85 (dd, *J* = 6.0 Hz, 2.0 Hz, 1H), 3.81 (dd, *J* = 6.0 Hz, 2.0 Hz, 1H), 3.75 (s, 3H); ^{13}C NMR (125 MHz, $CDCl_3$) δ 192.6,

162.1, 144.0, 142.4, 141.0, 139.9, 138.6, 133.3, 132.4, 128.0, 127.9, 127.8, 127.6, 126.9, 126.9, 126.6, 126.6, 126.5, 126.3, 126.2, 126.1, 125.8, 125.2, 123.6, 123.5, 56.8, 53.0, 52.0, 46.9, 46.9; HPLC analysis: Daicel Chiralpak AD-H, hexane/iso-propanol = 97:3, flow rate = 1.0 mL/min, λ = 267 nm, retention time: 16.1 min (minor) and 23.1 min (major); IR (KBr, cm^{-1}):3053, 3023, 2951, 1729, 1466, 1458, 1262, 1079, 818, 746; HRMS (ESI) calcd for $C_{29}H_{21}O_3^-$: 417.1496, found: 417.1494.

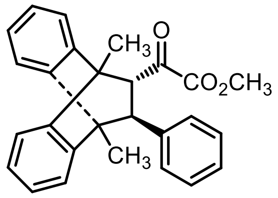

5o

5o: Colorless solid; 93% yield, 23% *ee*;; ^{1}H NMR (400 MHz, $CDCl_3$) δ 7.41 (d, *J* = 7.2 Hz, 1H), 7.35 (d, *J* = 6.4 Hz, 1H), 7.28–7.04 (m, 9H), 6.38 (br, 2H), 3.80 (d, *J* = 6.8 Hz, 1H), 3.56 (s, 3H), 3.11 (d, *J* = 6.4 Hz, 1H), 2.01 (s, 3H), 1.55 (s, 3H); ^{13}C NMR (100 MHz, $CDCl_3$) δ 195.3, 162.4, 146.9, 145.7, 143.2, 141.4, 141.1, 128.1, 127.5, 127.1, 126.3, 126.2, 126.1, 125.7, 123.6, 122.8, 120.4, 120.4, 120.4, 61.3, 56.4, 52.9, 46.1, 45.4, 16.5, 16.2; HPLC analysis: Daicel Chiralpak AD-H (2x), hexane/iso-propanol = 97:3, flow rate = 0.5 mL/min, λ = 206 nm, retention time: 32.6 min (major) and 34.1 min (minor); IR (KBr, cm^{-1}): 3063, 3028, 1969, 2881, 1733, 1493, 1453, 1381, 1271, 1096, 1068, 765, 750, 702; HRMS (ESI) calcd for $C_{27}H_{24}O_3Na^+$: 419.1618, found: 419.1618.

Procedure for gram-scale reaction of 3a and 4k, and transformation to cycloadduct 5k: To dichloromethane was added $FeBr_3$ (0.5 mmol, 10 mol %) and silver

salt of chiral phosphoric acid (1 mmol, 20 mol %). After stirring for 3 h, Ph_3CCl (0.5 mmol, 10 mol %) was added and the reaction mixture was stirred for 3 h at rt. After the addition of the β,γ-unsaturated-α-ketoester **4k** (5 mmol) and anthracene (**3a**, 10 mmol), the reaction mixture was stirred for 3 d at rt. The reaction mixture was purified by column chromatography on silica gel with petroleum ether/dichloromethane 2:1 to afford the desired product **5k** in 88% yield and 87% ee. To an oven-dried reaction tube was added cycloadduct **5k** (4.4 mmol), sulfonyl hydrazide **6** (5.3 mmol), and $MgSO_4$ (22.0 mmol) and distilled anhydrous CH_2Cl_2 was added. The mixture was stirred for 12 h at rt. Purification of the mixture by column chromatography on silica gel gave product **7**.

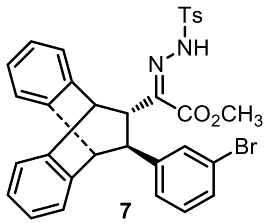

7

7: colorless solid; 83% yield, 82% *ee*; $[\alpha]_D^{25}$ = +226.7; ^{1}H NMR (500 MHz, $CDCl_3$) δ 11.52 (s, 1H), 7.51 (d, *J* = 7.5 Hz, 2H), 7.37 (d, *J* = 7.0 Hz, 1H), 7.27–7.17 (m, 5H), 7.11-7.04 (m, 4H), 6.97-6.90 (m, 2H), 6.65 (s, 1H), 6.35 (d, *J* = 7.45 Hz, 1H), 4.42 (s, 1H), 4.12 (s, 1H), 3.65 (s, 3H), 3.39 (d, *J* = 5.5 Hz, 1H), 3.20 (d, *J* = 5.5 Hz, 1H), 2.44 (s, 3H); ^{13}C NMR (125 MHz, $CDCl_3$) δ 162.2, 146.1, 144.1, 144.0, 143.8, 140.3, 138.8, 136.7, 135.4, 131.2, 129.7, 129.7, 129.5, 127.8, 126.4, 126.4, 126.2, 126.1, 126.0, 125.9, 123.3, 122.6, 122.1, 52.6, 51.5, 50.6, 48.6, 48.1, 21.8; HPLC analysis: Daicel Chiralpak AD-H, hexane/iso-propanol = 90:10, flow rate = 1.0 mL/min, λ = 206 nm, retention time: 8.7 min (minor) and 14.9 min (major); IR (KBr, cm^{-1}): 3212, 3068, 3023, 2954,

1699, 1592, 1565, 1458, 1377, 1169, 1085, 761, 698, 665; HRMS (ESI) calcd for $C_{32}H_{27}O_4N_2BrNa^+$: 637.0767, found: 637.0764.

NMR Spectra

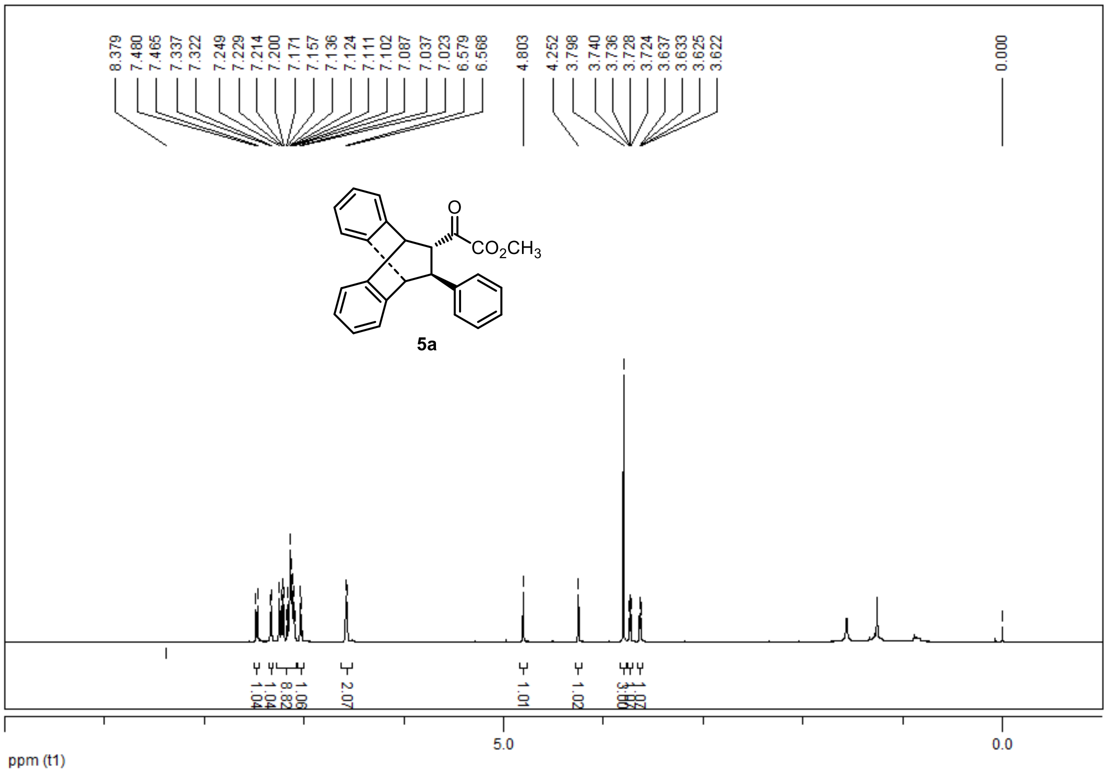
8.379
7.480
7.465
7.337
7.322
7.249
7.229
7.214
7.200
7.171
7.157
7.136
7.124
7.111
7.102
7.087
7.037
7.023
6.579
6.568
4.803
4.252
3.798
3.740
3.736
3.728
3.724
3.637
3.633
3.625
3.622
0.000
CO_2CH_3
5a
1.04
1.04
8.82
1.06
2.07
1.01
1.02
5.0
0.0
ppm (t1)

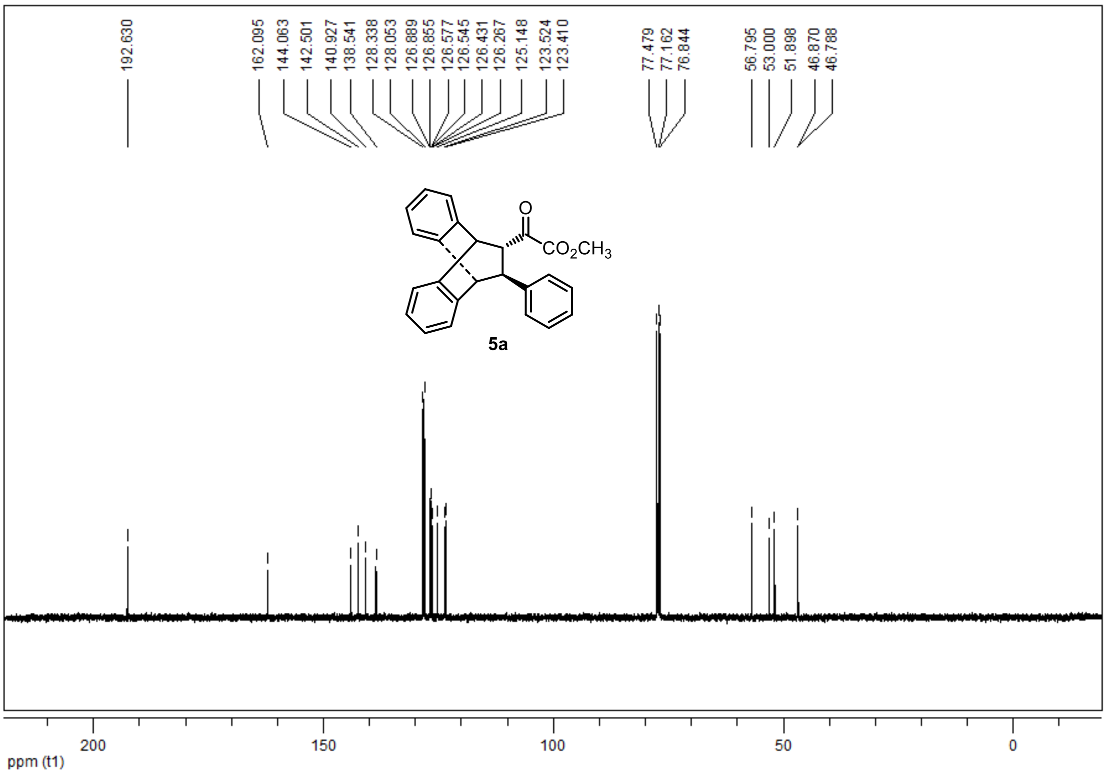
192.630
162.095
144.063
142.501
140.927
138.541
128.338
128.053
126.889
126.855
126.577
126.545
126.431
126.267
125.148
123.524
123.410
77.479
77.162
76.844
56.795
53.000
51.898
46.870
46.788
CO_2CH_3
5a
200
150
100
50
0
ppm (t1)

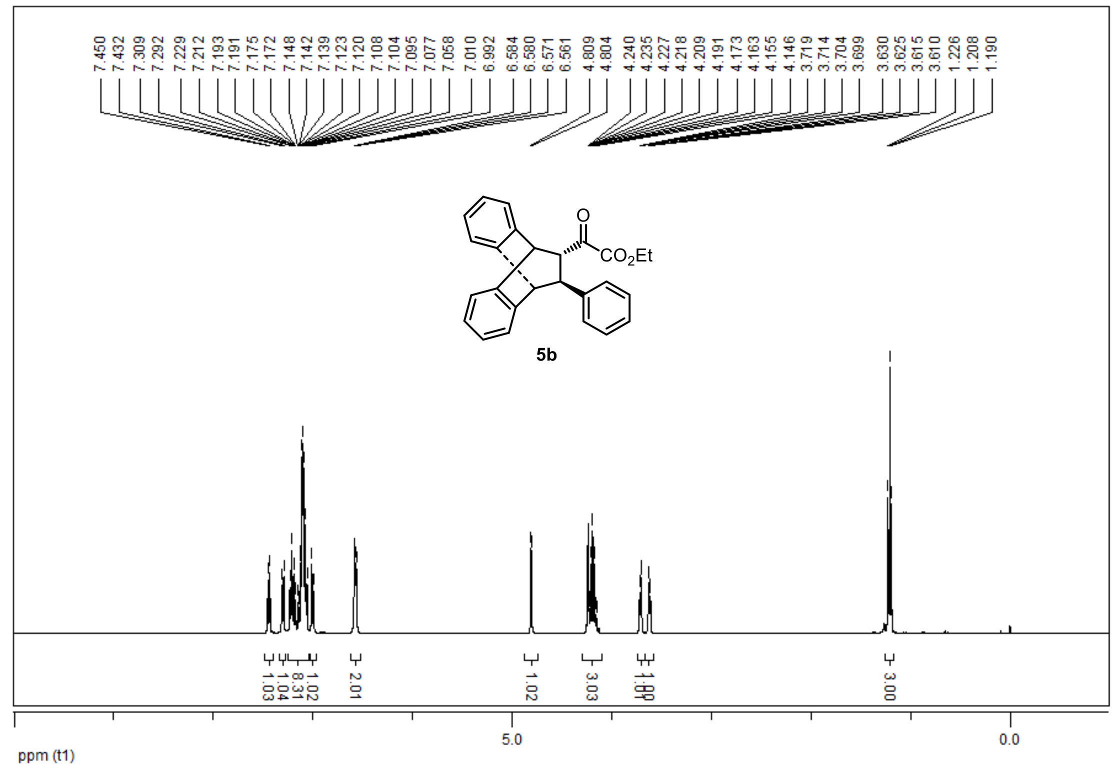

7.450
7.432
7.309
7.292
7.229
7.212
7.193
7.191
7.175
7.172
7.148
7.142
7.139
7.123
7.120
7.108
7.104
7.095
7.077
7.058
7.010
6.992
6.584
6.580
6.571
6.561
4.809
4.804
4.240
4.235
4.227
4.218
4.209
4.191
4.173
4.163
4.155
4.146
3.719
3.714
3.704
3.699
3.630
3.625
3.615
3.610
1.226
1.208
1.190
O
CO_2Et
5b
1.03
1.04
8.31
1.02
2.01
1.02
3.03
1.00
3.00
5.0
0.0
ppm (t1)

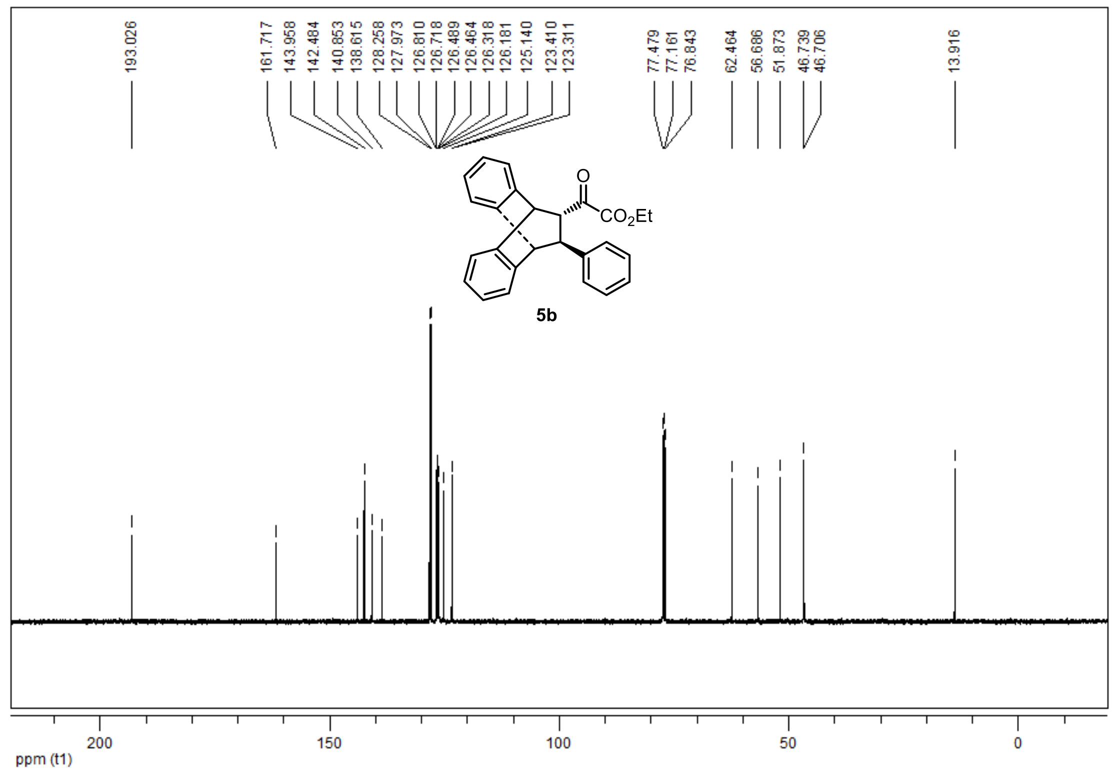

193.026
161.717
143.958
142.484
140.853
138.615
128.258
127.973
126.810
126.718
126.489
126.464
126.318
126.181
125.140
123.410
123.311
77.479
77.161
76.843
62.464
56.686
51.873
46.739
46.706
13.916
O
CO_2Et
5b
200
150
100
50
0
ppm (t1)

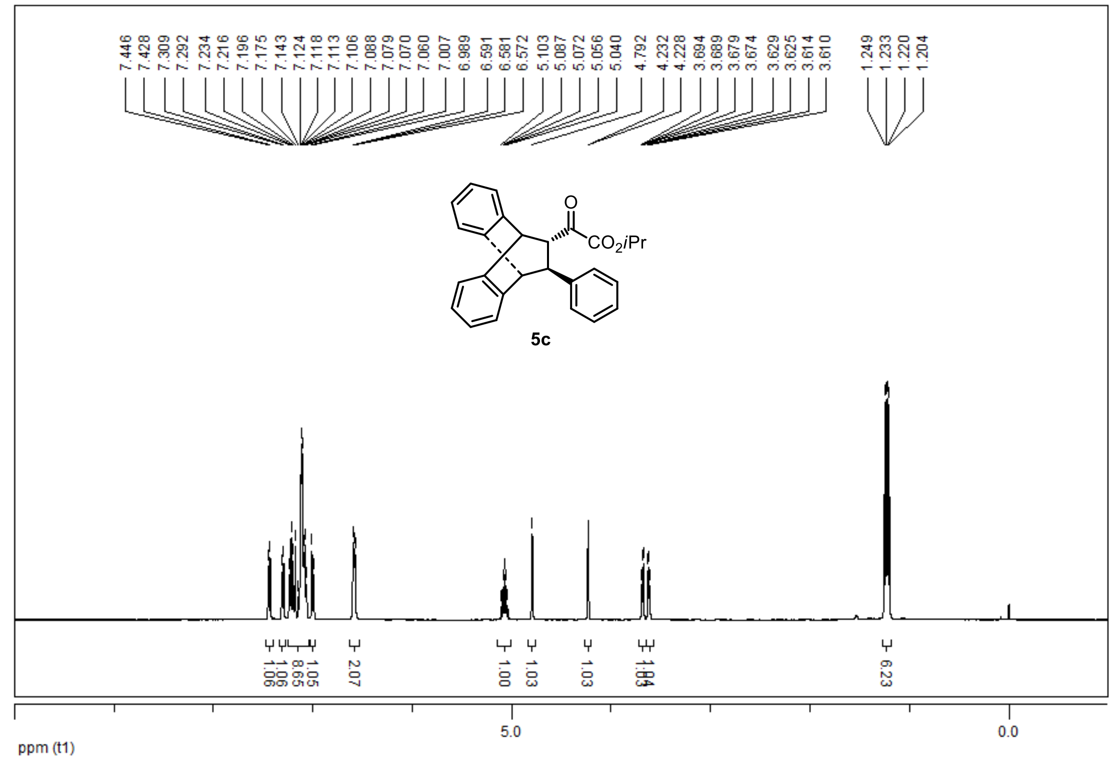

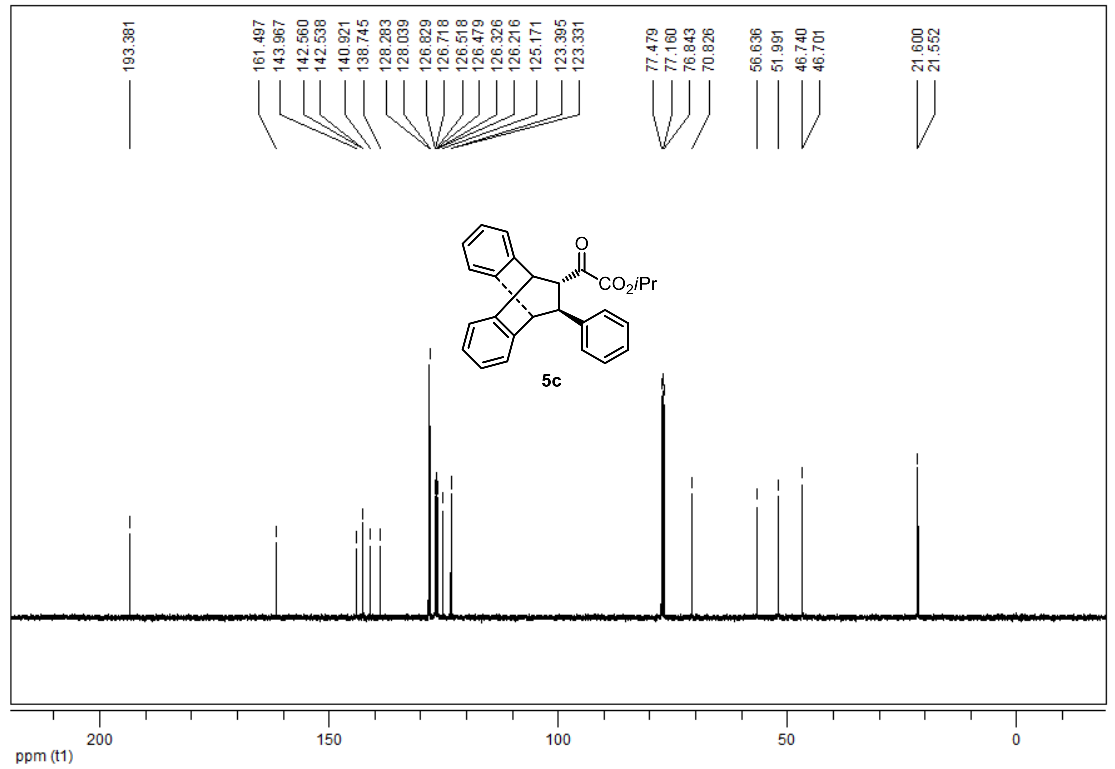

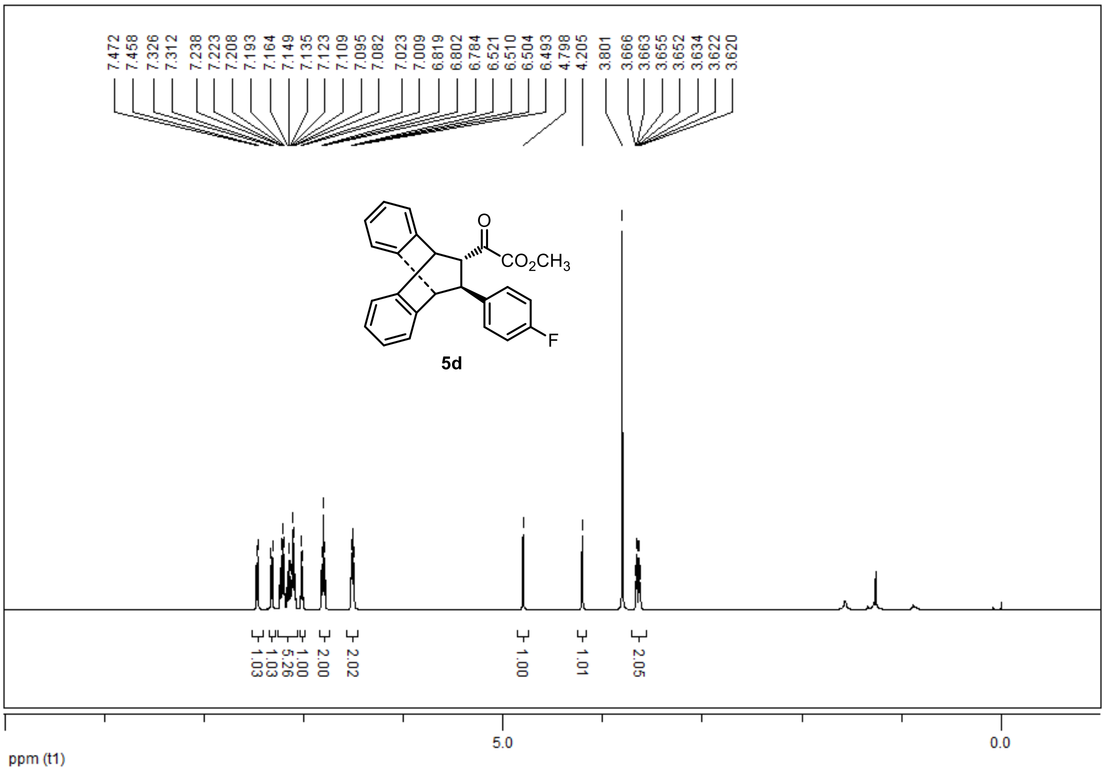

7.472
7.458
7.326
7.312
7.238
7.223
7.208
7.193
7.164
7.149
7.135
7.123
7.109
7.095
7.082
7.023
7.009
6.819
6.802
6.784
6.521
6.510
6.504
6.493
4.798
4.205
3.801
3.666
3.663
3.655
3.652
3.634
3.622
3.620
O
CO_2CH_3
F
5d
1.03
1.03
5.26
1.00
2.00
2.02
1.00
1.01
2.05
5.0
0.0
ppm (t1)

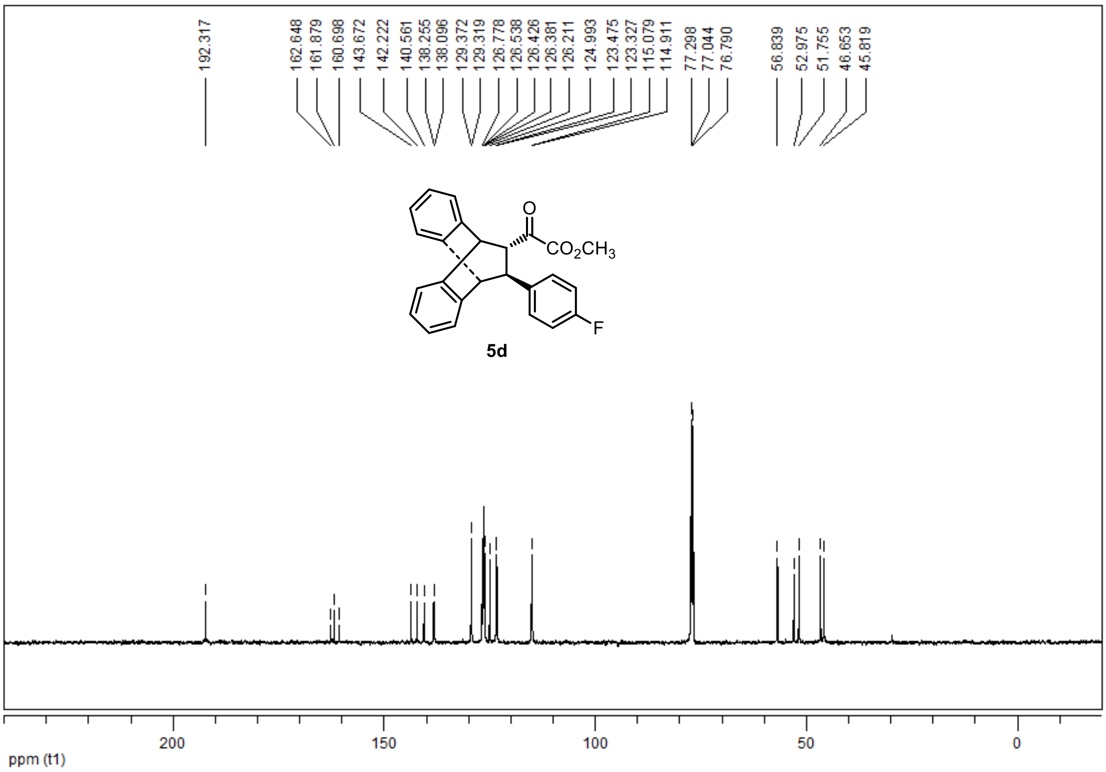

192.317
162.648
161.879
160.698
143.672
142.222
140.561
138.255
138.096
129.372
129.319
126.778
126.538
126.426
126.381
126.211
124.993
123.475
123.327
115.079
114.911
77.298
77.044
76.790
56.839
52.975
51.755
46.653
45.819
O
CO_2CH_3
F
5d
200
150
100
50
0
ppm (t1)

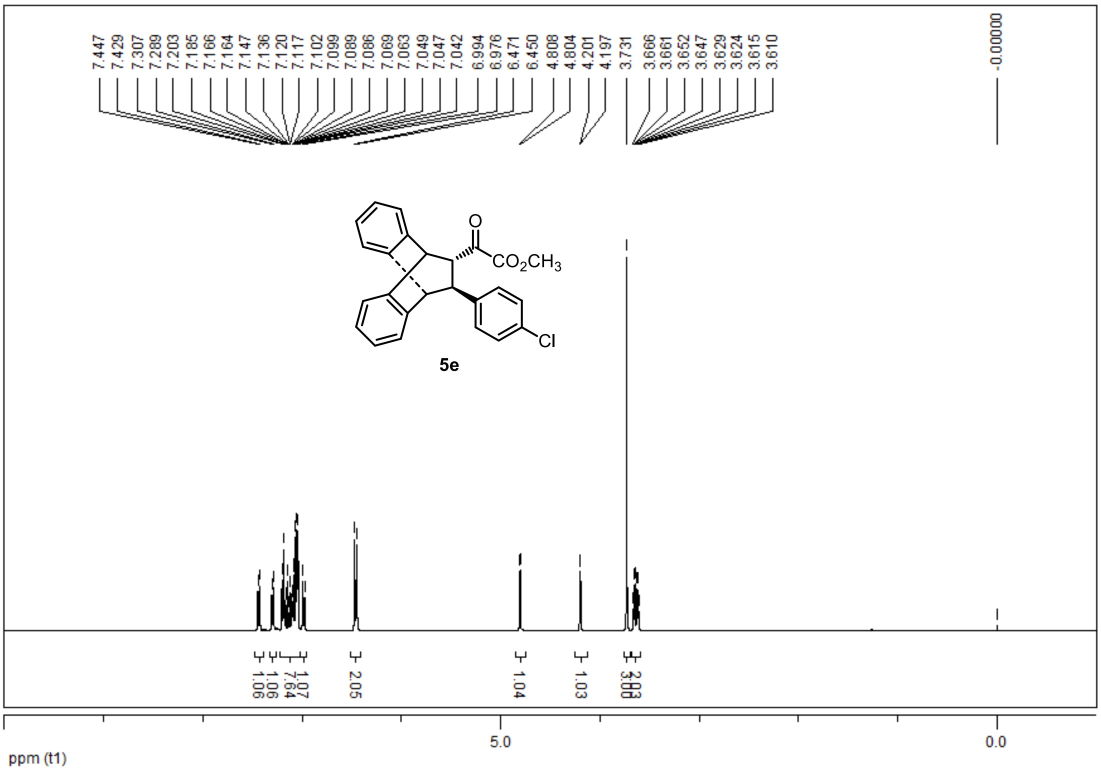
7.447
7.429
7.307
7.289
7.203
7.185
7.166
7.164
7.147
7.136
7.120
7.117
7.102
7.099
7.089
7.086
7.069
7.063
7.049
7.047
7.042
6.994
6.976
6.471
6.450
4.808
4.804
4.201
4.197
3.731
3.666
3.661
3.652
3.647
3.629
3.624
3.615
3.610
-0.00000
O
CO_2CH_3
Cl
5e
1.06
1.06
7.64
1.07
2.05
1.04
1.03
3.00
2.03
5.0
0.0
ppm (t1)

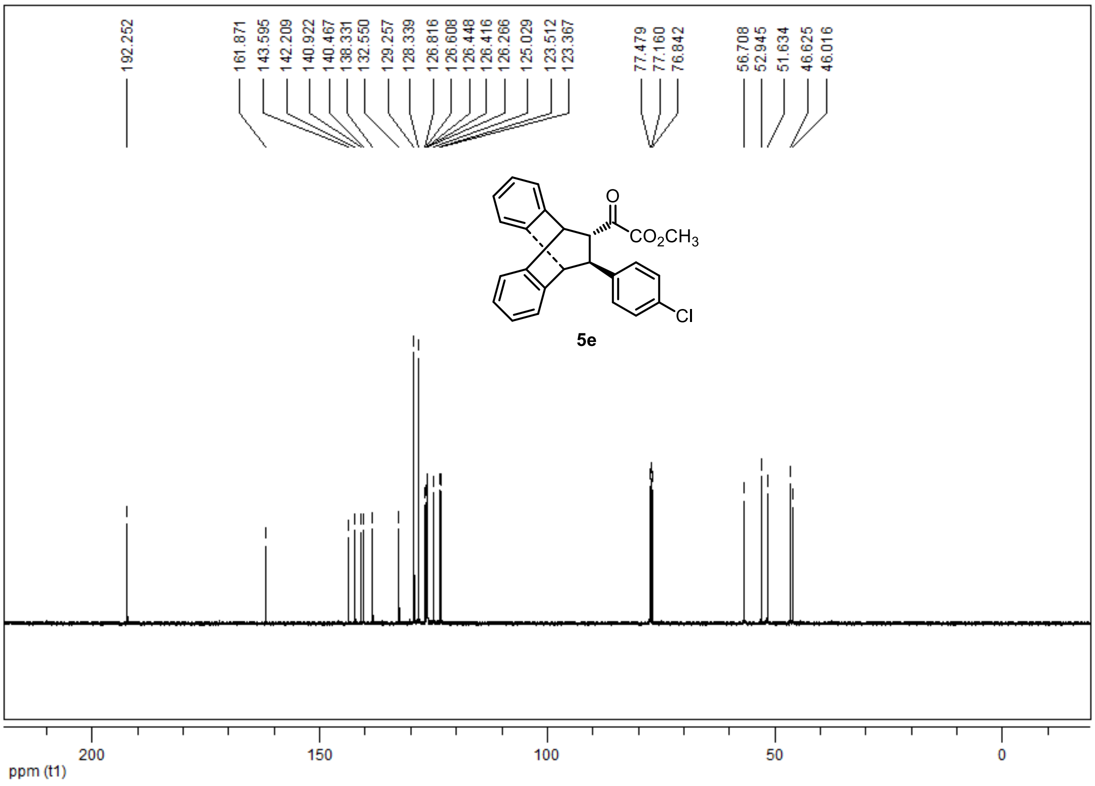
192.252
161.871
143.595
142.209
140.922
140.467
138.331
132.550
129.257
128.339
126.816
126.608
126.448
126.416
126.266
125.029
123.512
123.367
77.479
77.160
76.842
56.708
52.945
51.634
46.625
46.016
O
CO_2CH_3
Cl
5e
200
150
100
50
0
ppm (t1)

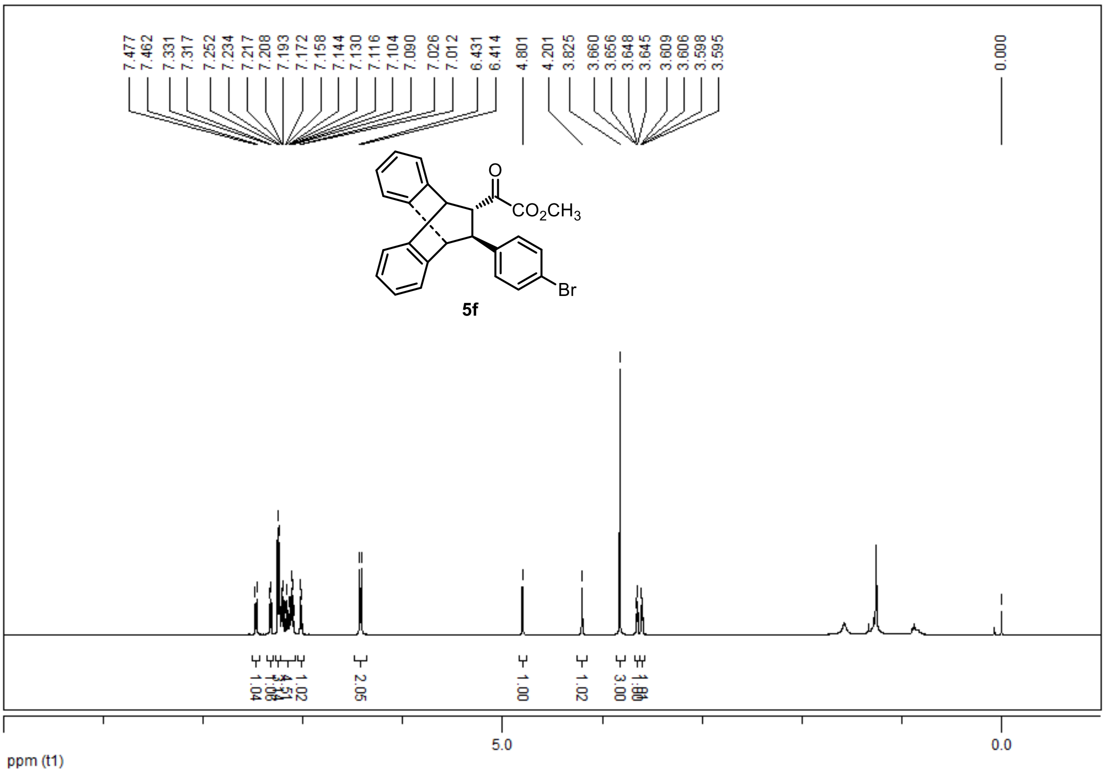
7.477
7.462
7.331
7.317
7.252
7.234
7.217
7.208
7.193
7.172
7.158
7.144
7.130
7.116
7.104
7.090
7.026
7.012
6.431
6.414
4.801
4.201
3.825
3.660
3.656
3.648
3.645
3.609
3.606
3.598
3.595
0.000
O
CO_2CH_3
Br
5f
2.05
1.00
1.02
3.00
5.0
0.0
ppm (t1)

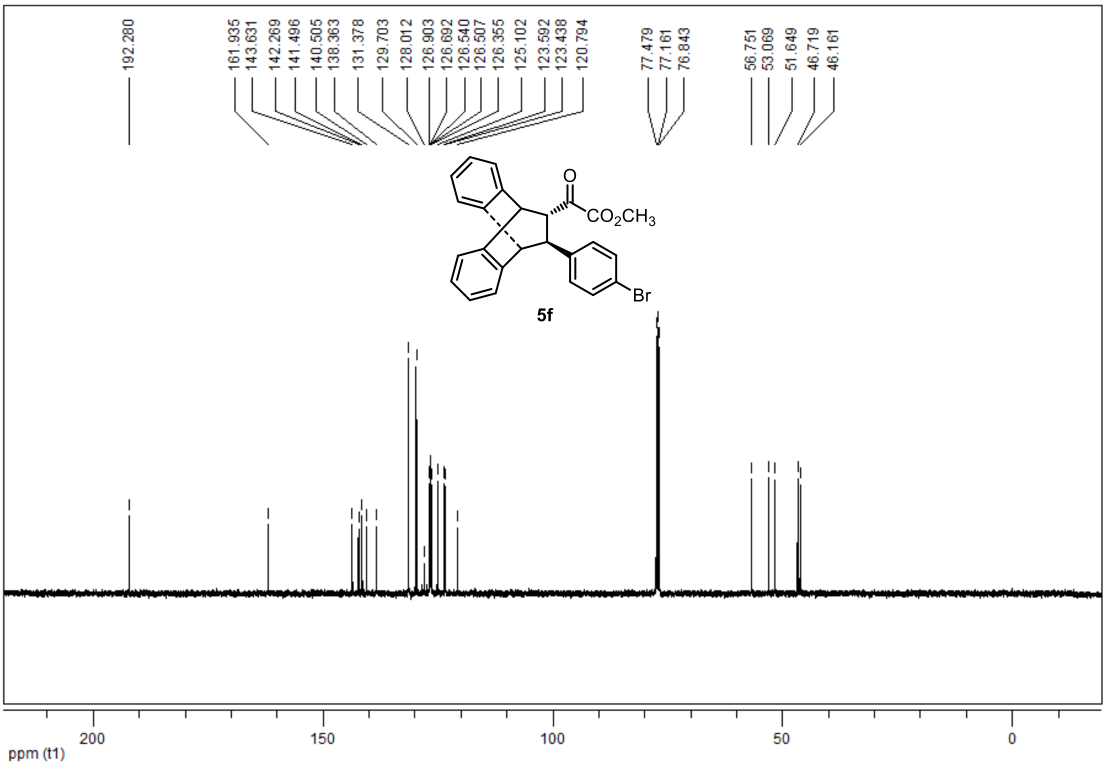
192.280
161.935
143.631
142.269
141.496
140.505
138.363
131.378
129.703
128.012
126.903
126.692
126.540
126.507
126.355
125.102
123.592
123.438
120.794
77.479
77.161
76.843
56.751
53.069
51.649
46.719
46.161
O
CO_2CH_3
Br
5f
200
150
100
50
0
ppm (t1)

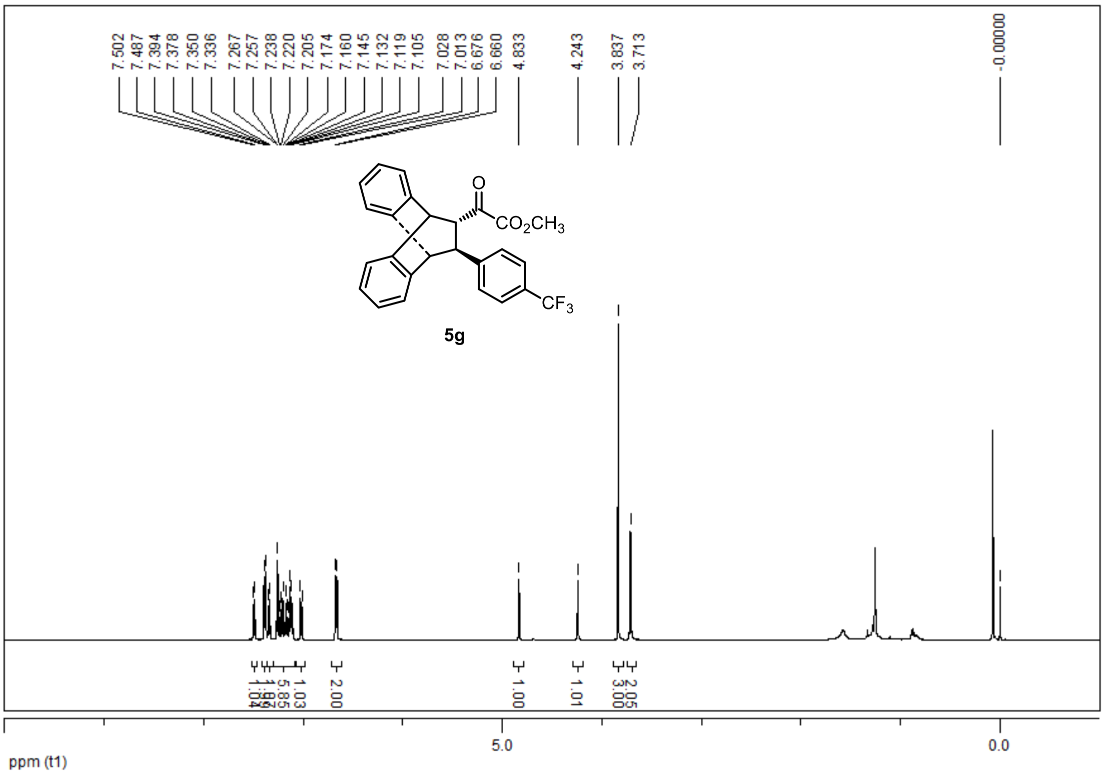
7.502
7.487
7.394
7.378
7.350
7.336
7.267
7.257
7.238
7.220
7.205
7.174
7.160
7.145
7.132
7.119
7.105
7.028
7.013
6.676
6.660
4.833
4.243
3.837
3.713
-0.00000
O
CO2CH3
CF3
5g
1.04
5.85
1.03
2.00
1.00
1.01
3.00
2.05
5.0
0.0
ppm (t1)

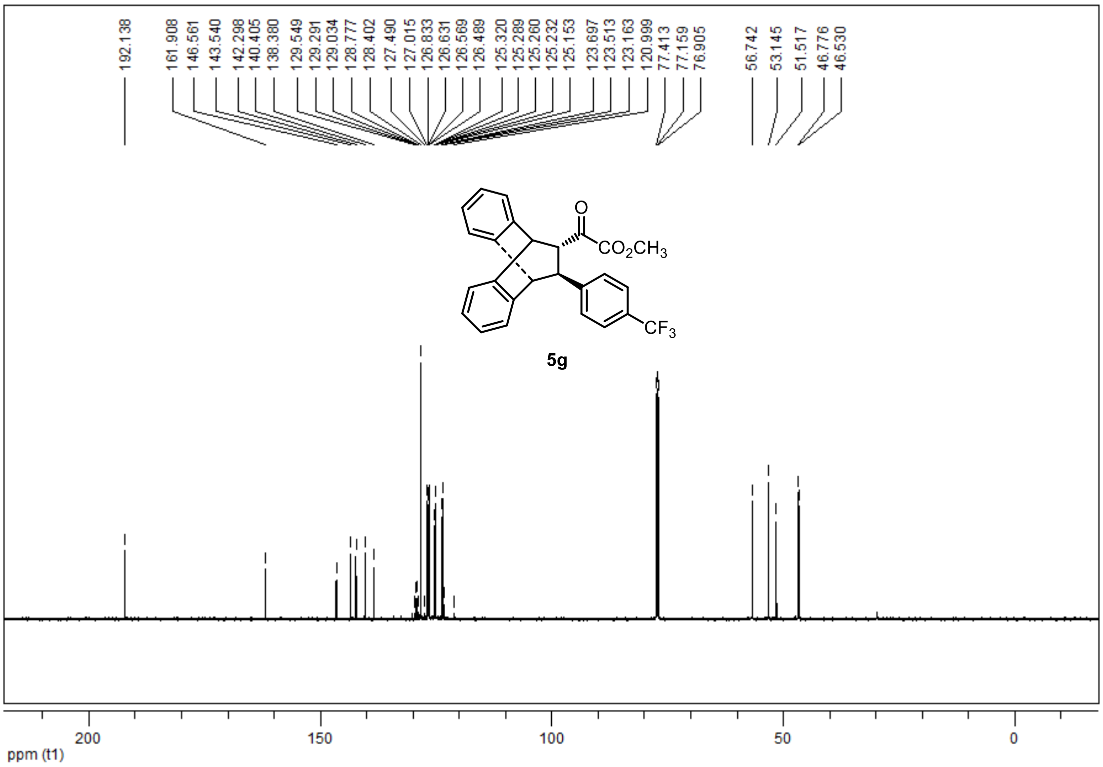
192.138
161.908
146.561
143.540
142.298
140.405
138.380
129.549
129.291
129.034
128.777
128.402
127.490
127.015
126.833
126.631
126.569
126.489
125.320
125.289
125.260
125.232
125.153
123.697
123.513
123.163
120.999
77.413
77.159
76.905
56.742
53.145
51.517
46.776
46.530
O
CO2CH3
CF3
5g
200
150
100
50
0
ppm (t1)

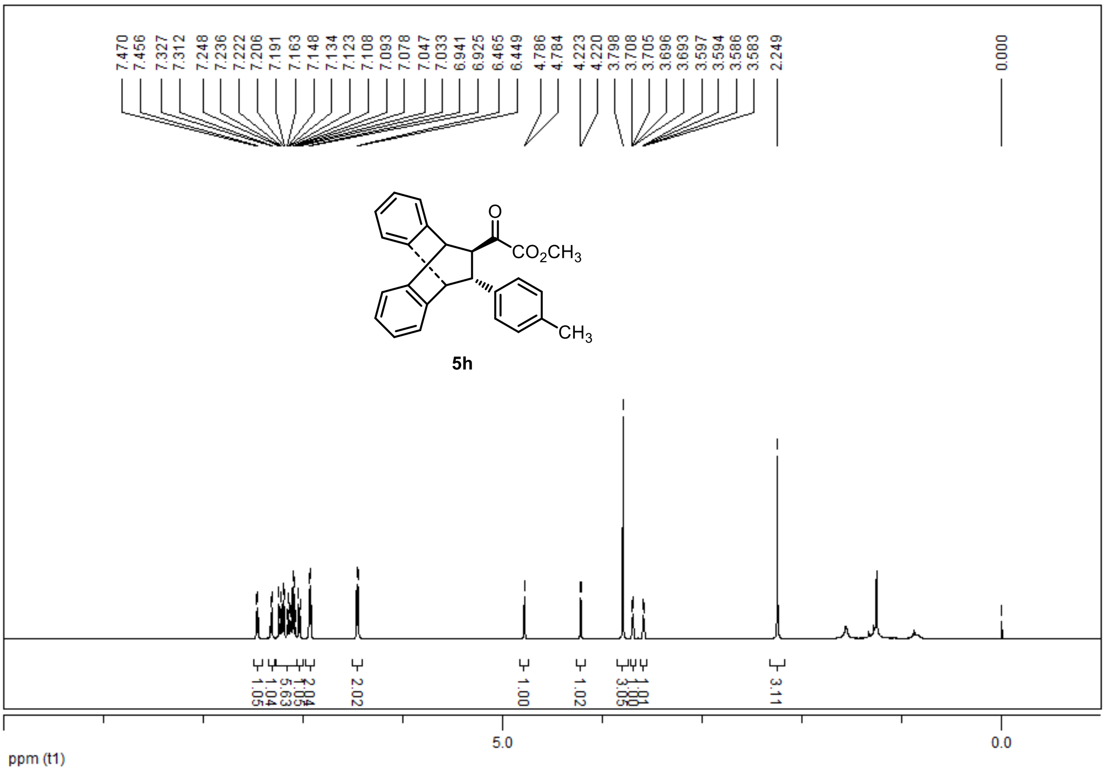
7.470
7.456
7.327
7.312
7.248
7.236
7.222
7.206
7.191
7.163
7.148
7.134
7.123
7.108
7.093
7.078
7.047
7.033
6.941
6.925
6.465
6.449
4.786
4.784
4.223
4.220
3.798
3.708
3.705
3.696
3.693
3.597
3.594
3.586
3.583
2.249
0.000
O
CO_2CH_3
CH_3
5h
1.05
1.04
5.63
1.05
2.04
2.02
1.00
1.02
3.05
1.01
3.11
5.0
0.0
ppm (t1)

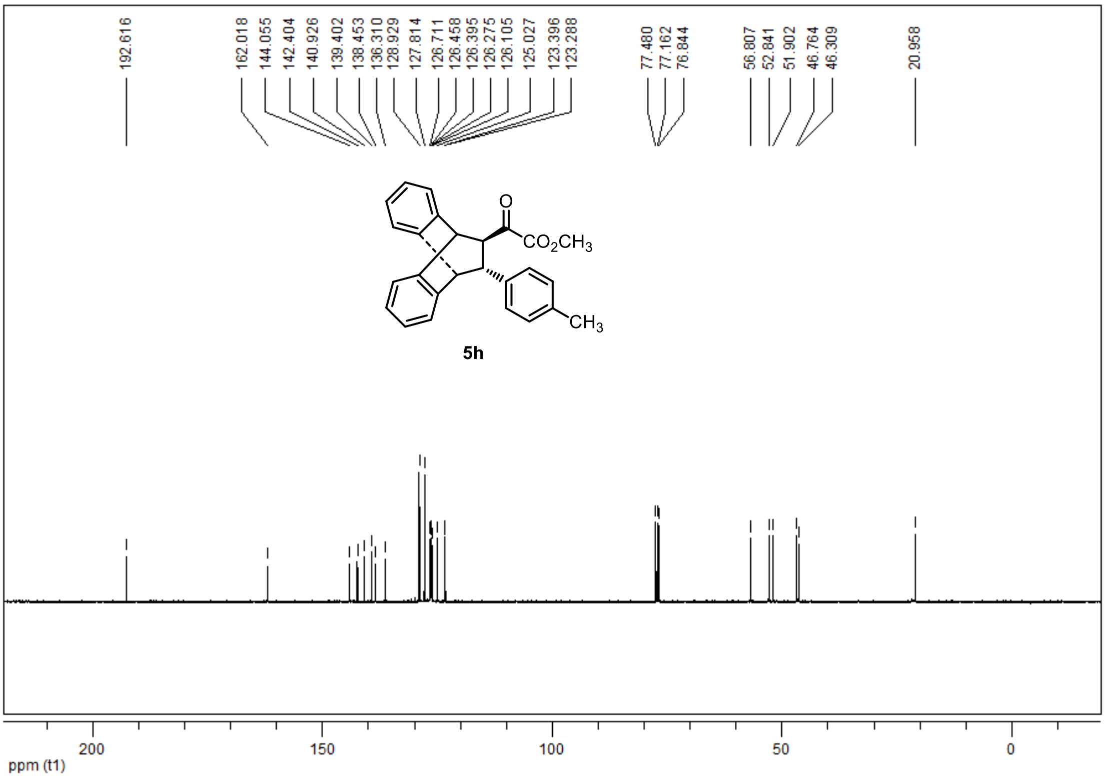
192.616
162.018
144.055
142.404
140.926
139.402
138.453
136.310
128.929
127.814
126.711
126.458
126.395
126.275
126.105
125.027
123.396
123.288
77.480
77.162
76.844
56.807
52.841
51.902
46.764
46.309
20.958
O
CO_2CH_3
CH_3
5h
200
150
100
50
0
ppm (t1)

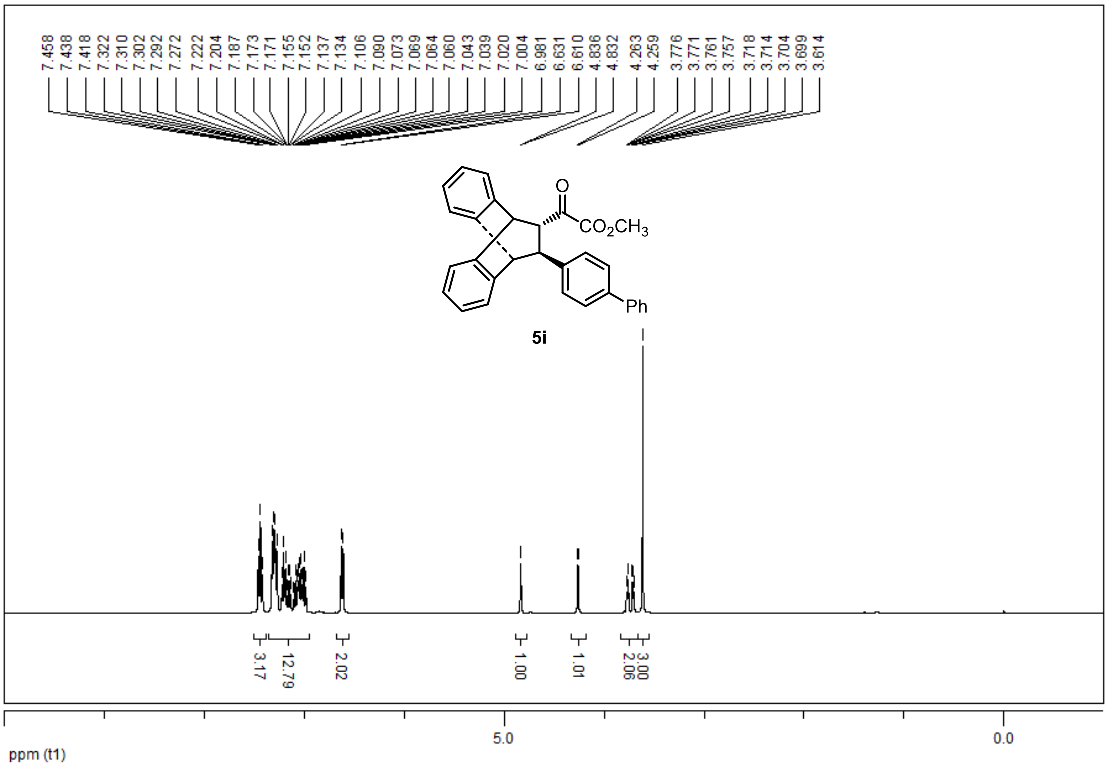
7.458
7.438
7.418
7.322
7.310
7.302
7.292
7.272
7.222
7.204
7.187
7.173
7.171
7.155
7.152
7.137
7.134
7.106
7.090
7.073
7.069
7.064
7.060
7.043
7.039
7.020
7.004
6.981
6.631
6.610
4.836
4.832
4.263
4.259
3.776
3.771
3.761
3.757
3.718
3.714
3.704
3.699
3.614
O
CO_2CH_3
Ph
5i
3.17
12.79
2.02
1.00
1.01
2.06
3.00
5.0
0.0
ppm (t1)

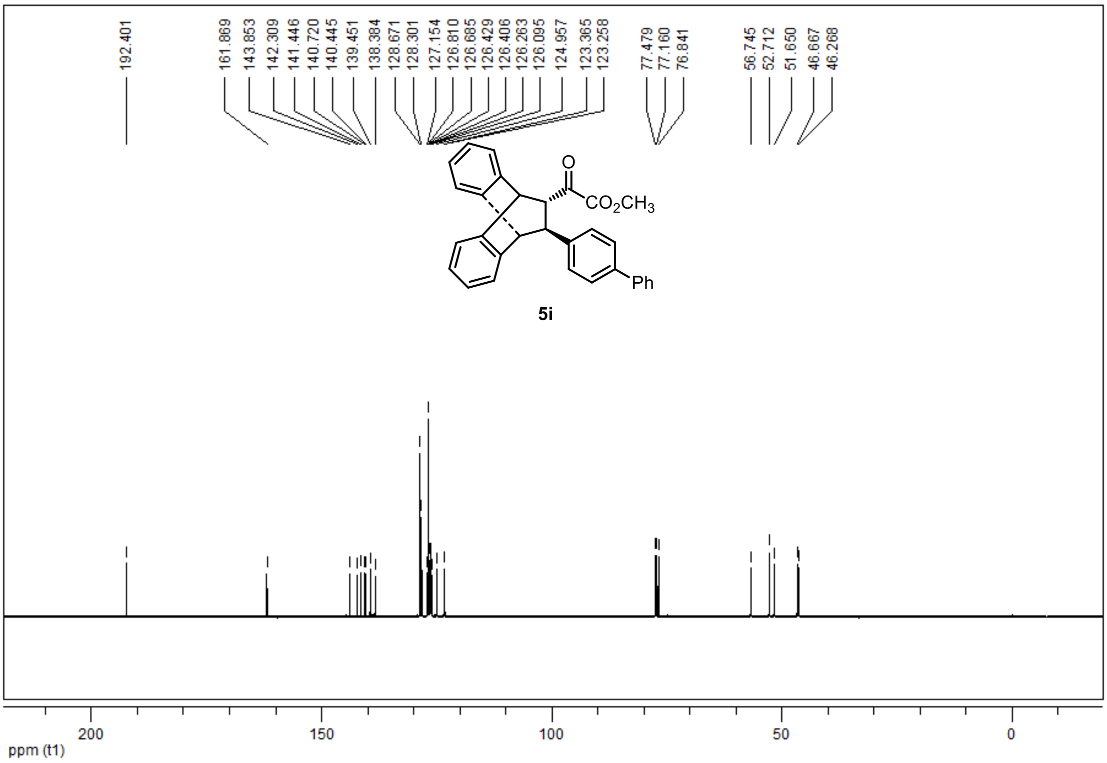
192.401
161.869
143.853
142.309
141.446
140.720
140.445
139.451
138.384
128.671
128.301
127.154
126.810
126.685
126.429
126.406
126.263
126.095
124.957
123.365
123.258
77.479
77.160
76.841
56.745
52.712
51.650
46.667
46.268
O
CO_2CH_3
Ph
5i
200
150
100
50
0
ppm (t1)

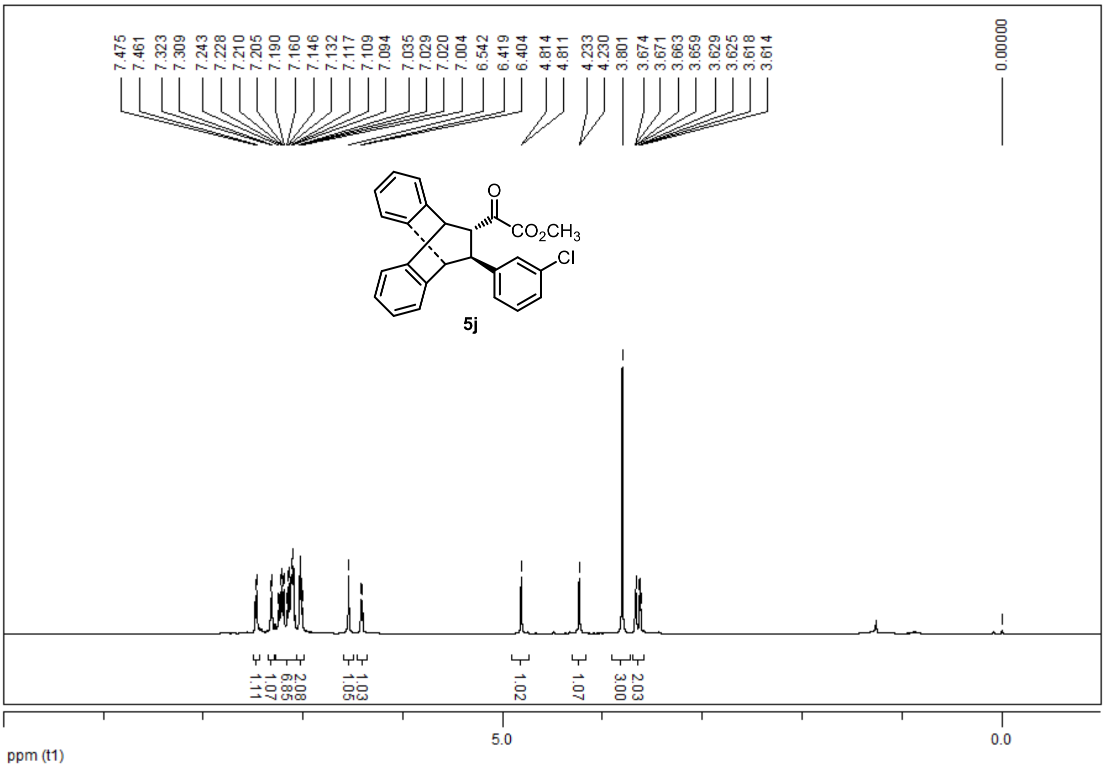
7.475
7.461
7.323
7.309
7.243
7.228
7.210
7.205
7.190
7.160
7.146
7.132
7.117
7.109
7.094
7.035
7.029
7.020
7.004
6.542
6.419
6.404
4.814
4.811
4.233
4.230
3.801
3.674
3.671
3.663
3.659
3.629
3.625
3.618
3.614
0.00000
O
CO2CH3
Cl
5j
1.11
1.07
6.85
2.08
1.05
1.03
1.02
1.07
3.00
2.03
5.0
0.0
ppm (t1)

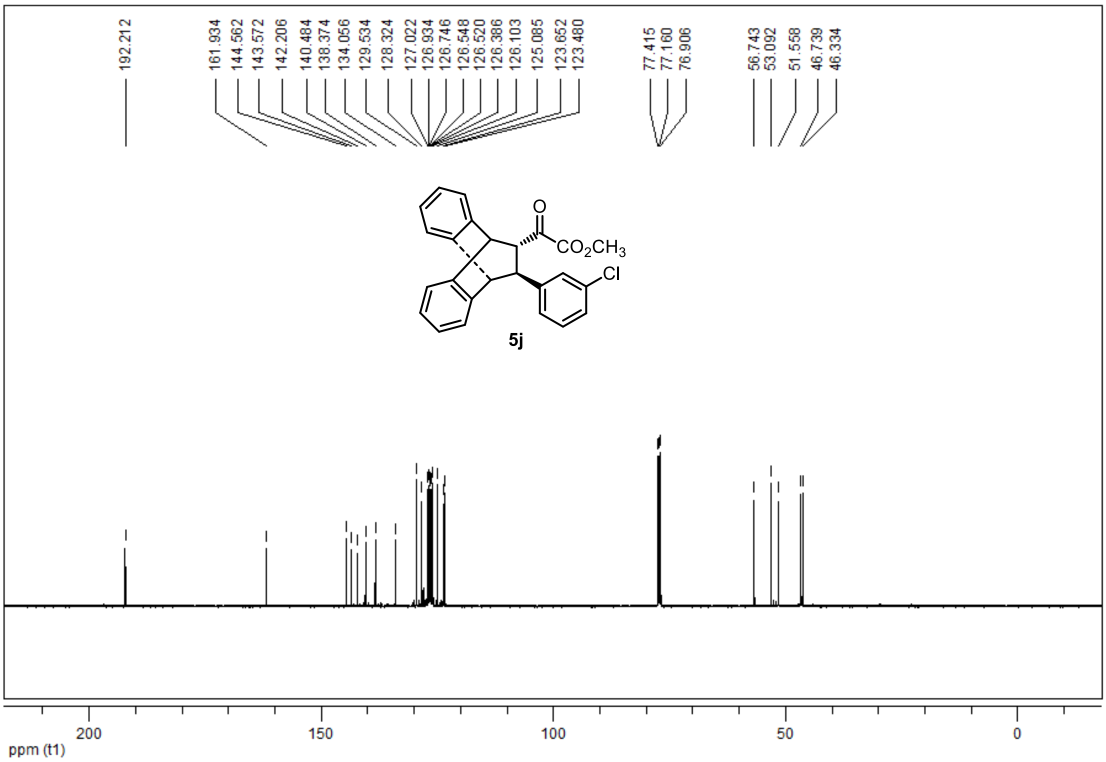
192.212
161.934
144.562
143.572
142.206
140.484
138.374
134.056
129.534
128.324
127.022
126.934
126.746
126.548
126.520
126.386
126.103
125.085
123.652
123.480
77.415
77.160
76.906
56.743
53.092
51.558
46.739
46.334
O
CO2CH3
Cl
5j
200
150
100
50
0
ppm (t1)

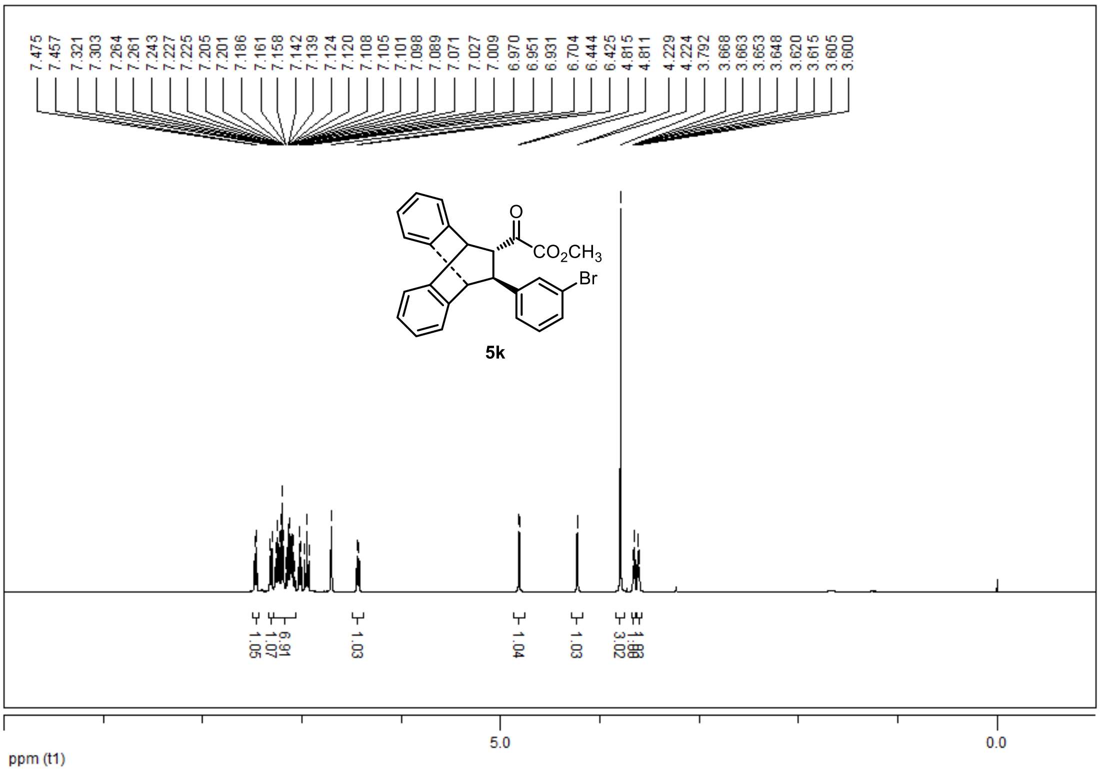
7.475 7.457 7.321 7.303 7.264 7.261 7.243 7.227 7.225 7.205 7.201 7.186 7.161 7.158 7.142 7.139 7.124 7.120 7.108 7.105 7.101 7.098 7.089 7.071 7.027 7.009 6.970 6.951 6.931 6.704 6.444 6.425 4.815 4.811 4.229 4.224 3.792 3.668 3.663 3.653 3.648 3.620 3.615 3.605 3.600
O
CO_2CH_3
Br
5k
1.05 1.07 6.91 1.03 1.04 1.03 3.02
5.0
0.0
ppm (t1)

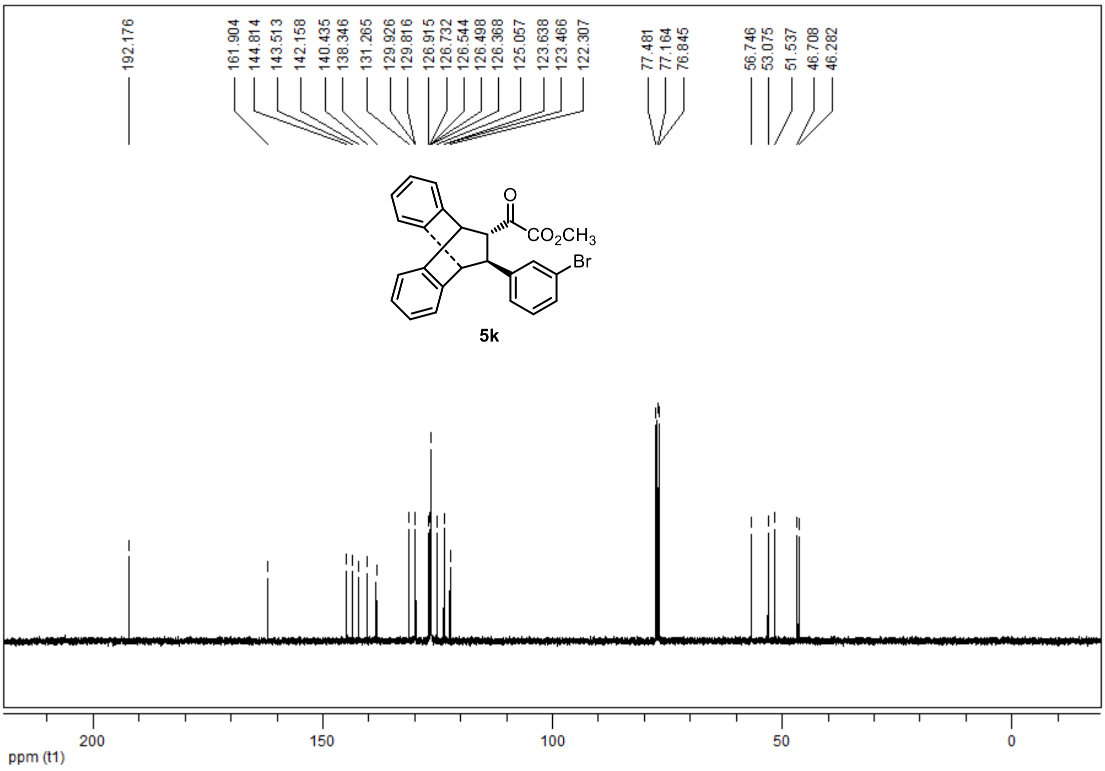
192.176
161.904 144.814 143.513 142.158 140.435 138.346 131.265 129.926 129.816 126.915 126.732 126.544 126.498 126.368 125.057 123.638 123.466 122.307
77.481 77.164 76.845
56.746 53.075 51.537 46.708 46.282
O
CO_2CH_3
Br
5k
200
150
100
50
0
ppm (t1)

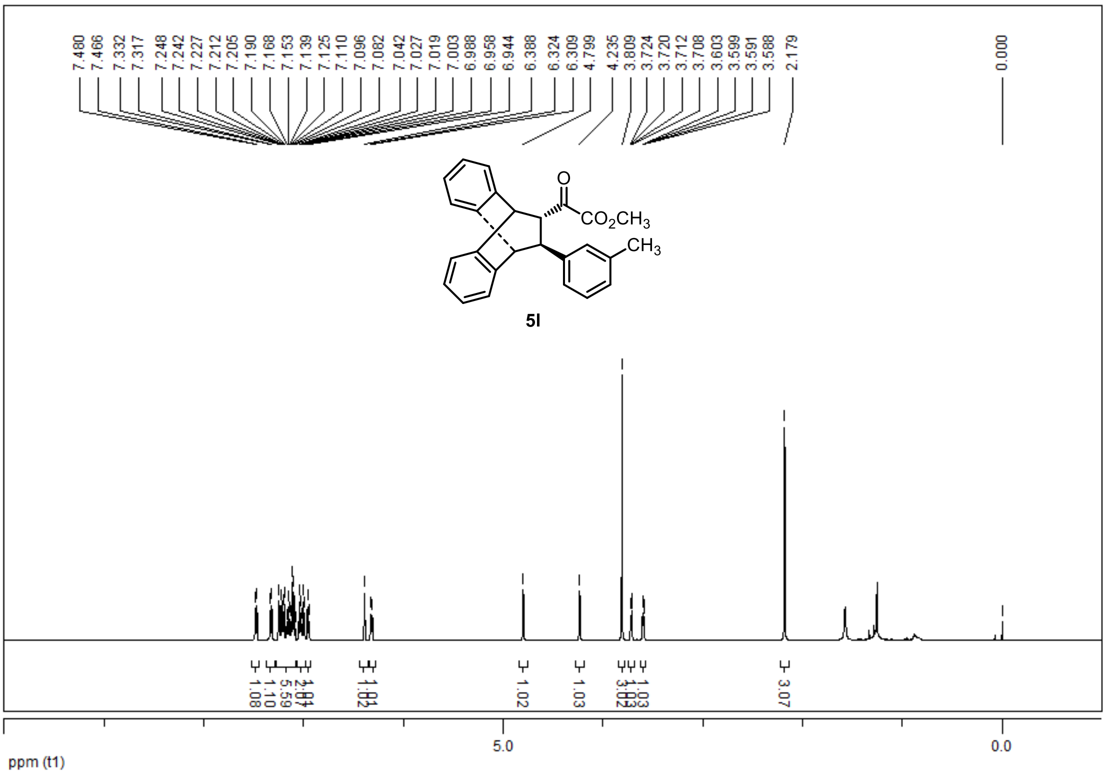
7.480
7.466
7.332
7.317
7.248
7.242
7.227
7.212
7.205
7.190
7.168
7.153
7.139
7.125
7.110
7.096
7.082
7.042
7.027
7.019
7.003
6.988
6.958
6.944
6.388
6.324
6.309
4.799
4.235
3.809
3.724
3.720
3.712
3.708
3.603
3.599
3.591
3.588
2.179
0.000
O
CO_2CH_3
CH_3
5l
1.08
1.10
5.59
1.02
1.03
1.03
3.07
5.0
0.0
ppm (t1)

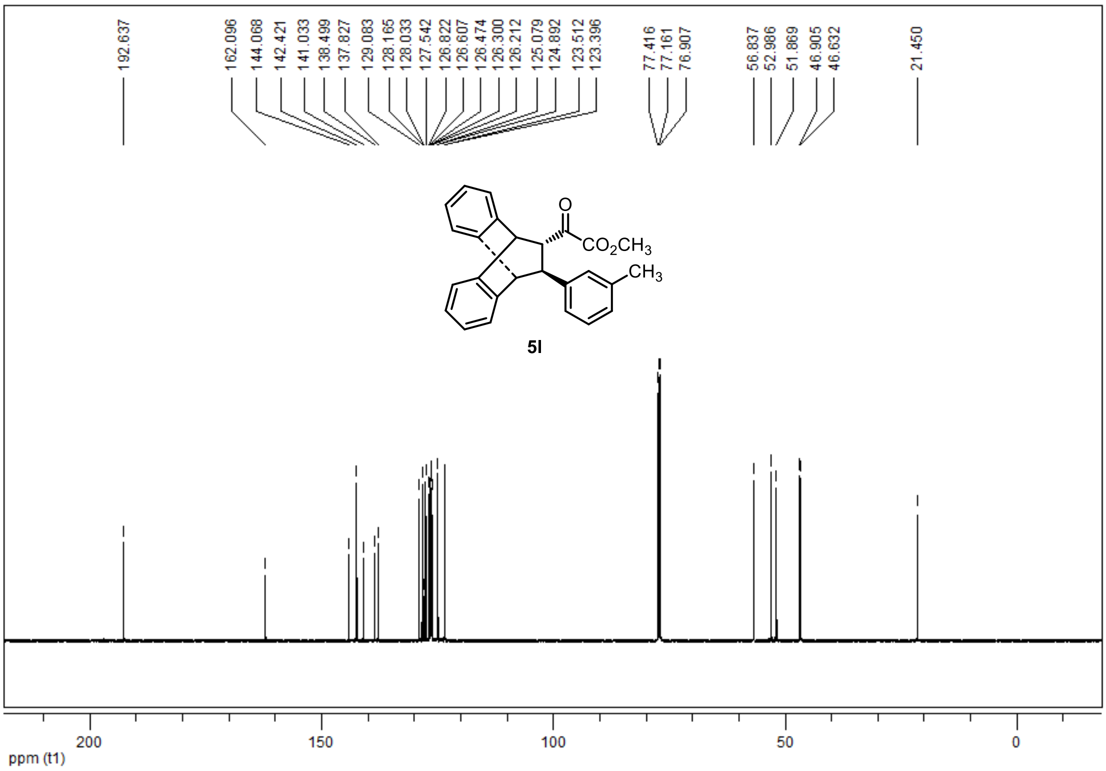
192.637
162.096
144.068
142.421
141.033
138.499
137.827
129.083
128.165
128.033
127.542
126.822
126.607
126.474
126.300
126.212
125.079
124.892
123.512
123.396
77.416
77.161
76.907
56.837
52.986
51.869
46.905
46.632
21.450
O
CO_2CH_3
CH_3
5l
200
150
100
50
0
ppm (t1)

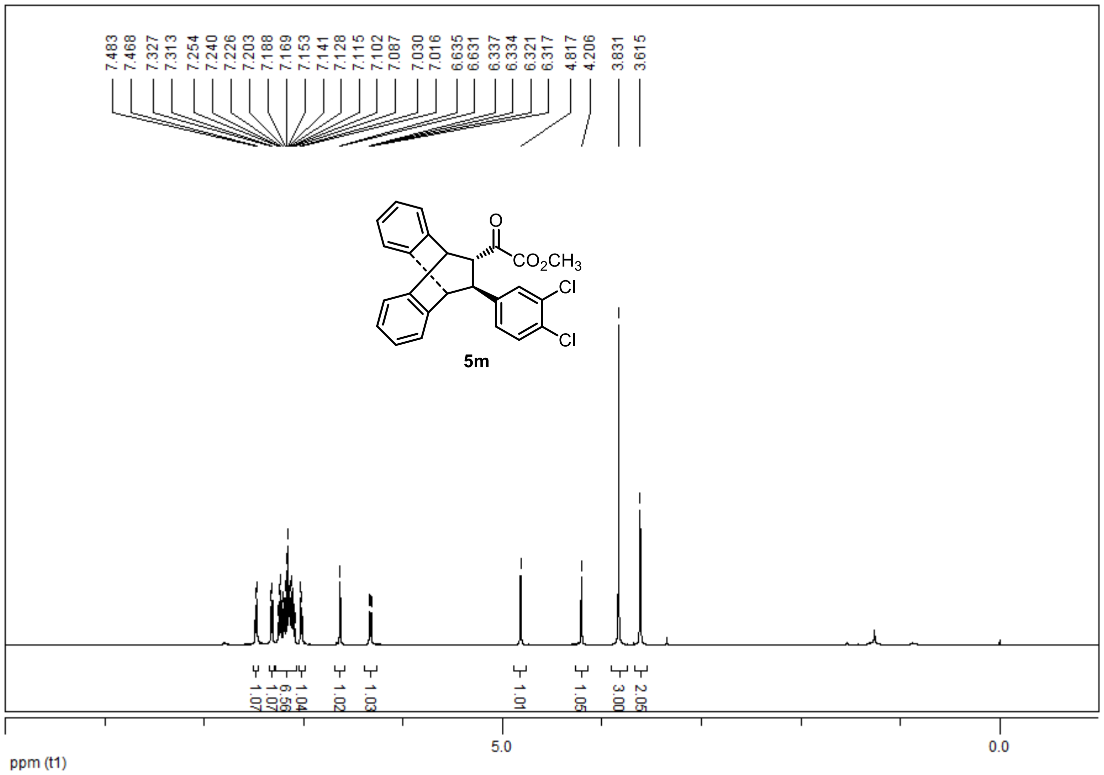
7.483
7.468
7.327
7.313
7.254
7.240
7.226
7.203
7.188
7.169
7.153
7.141
7.128
7.115
7.102
7.087
7.030
7.016
6.635
6.631
6.337
6.334
6.321
6.317
4.817
4.206
3.831
3.615
O
CO2CH3
Cl
Cl
5m
1.07
1.07
6.56
1.04
1.02
1.03
1.01
1.05
3.00
2.05
5.0
0.0
ppm (t1)

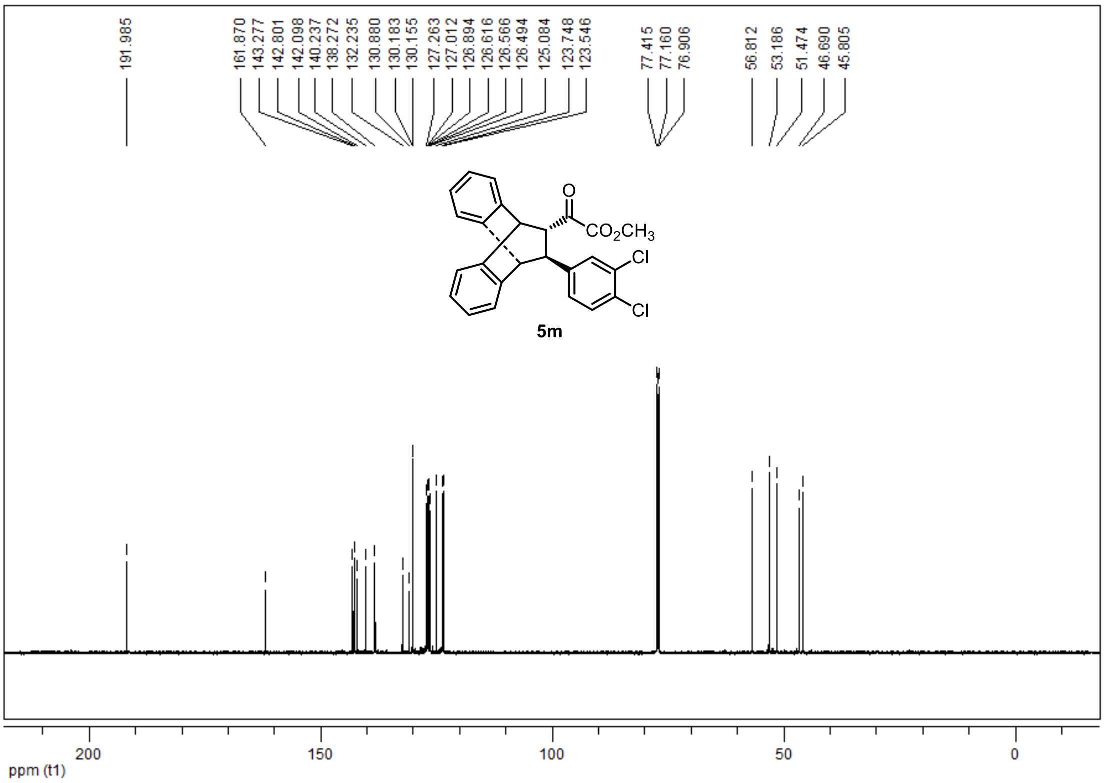
191.985
161.870
143.277
142.801
142.098
140.237
138.272
132.235
130.880
130.183
130.155
127.263
127.012
126.894
126.616
126.566
126.494
125.084
123.748
123.546
77.415
77.160
76.906
56.812
53.186
51.474
46.690
45.805
O
CO2CH3
Cl
Cl
5m
200
150
100
50
0
ppm (t1)

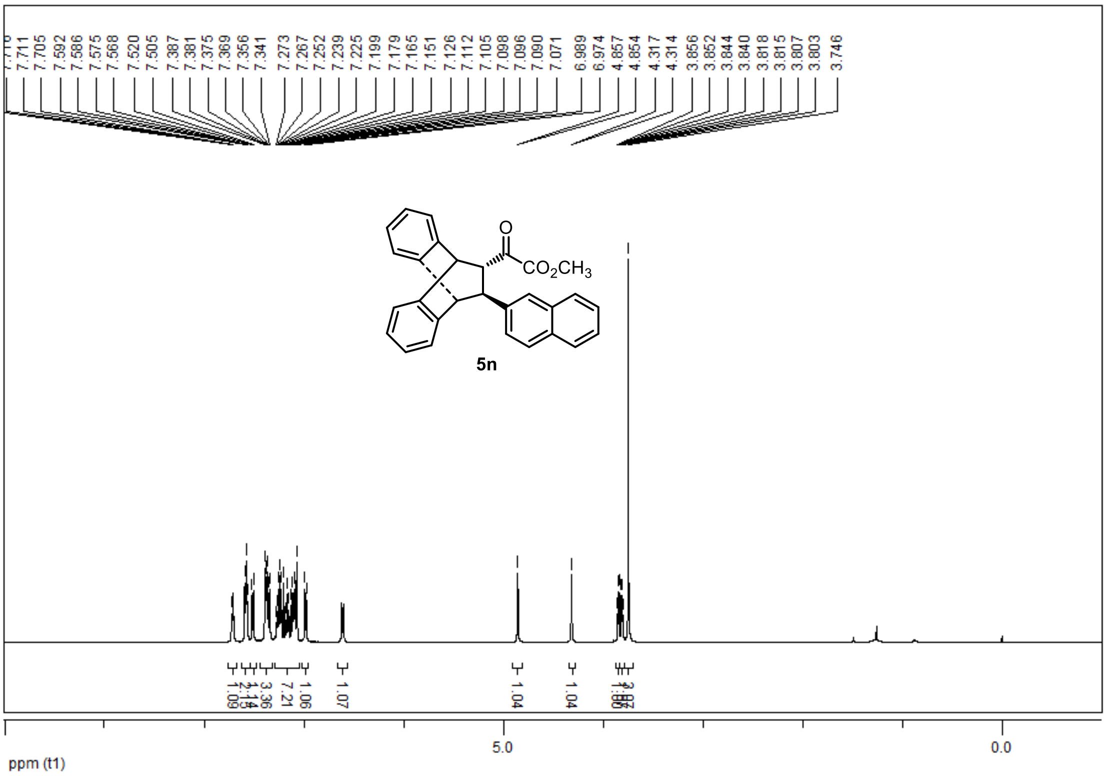

7.711
7.705
7.592
7.586
7.575
7.568
7.520
7.505
7.387
7.381
7.375
7.369
7.356
7.341
7.273
7.267
7.252
7.239
7.225
7.199
7.179
7.165
7.151
7.126
7.112
7.105
7.098
7.096
7.090
7.071
6.989
6.974
4.857
4.854
4.317
4.314
3.856
3.852
3.844
3.840
3.818
3.815
3.807
3.803
3.746
O
CO_2CH_3
5n
1.07
1.04
1.04
5.0
0.0
ppm (t1)

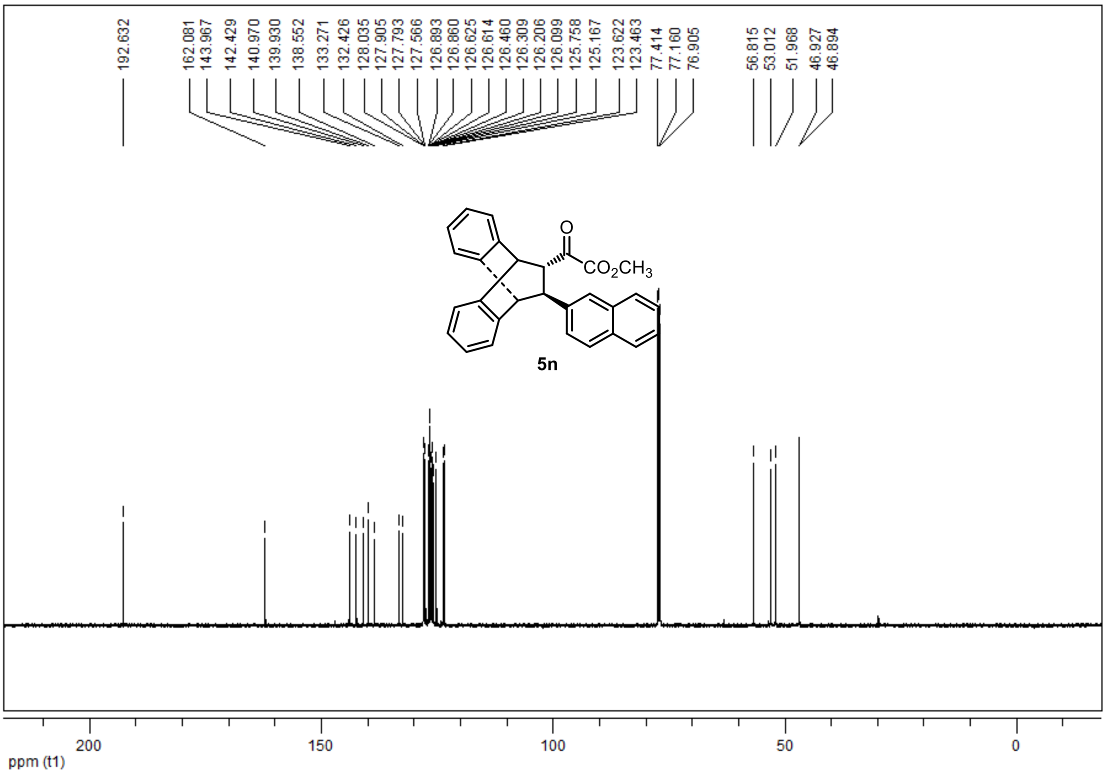

192.632
162.081
143.967
142.429
140.970
139.930
138.552
133.271
132.426
128.035
127.905
127.793
127.566
126.893
126.860
126.625
126.614
126.460
126.309
126.206
126.099
125.758
125.167
123.622
123.463
77.414
77.160
76.905
56.815
53.012
51.968
46.927
46.894
O
CO_2CH_3
5n
200
150
100
50
0
ppm (t1)

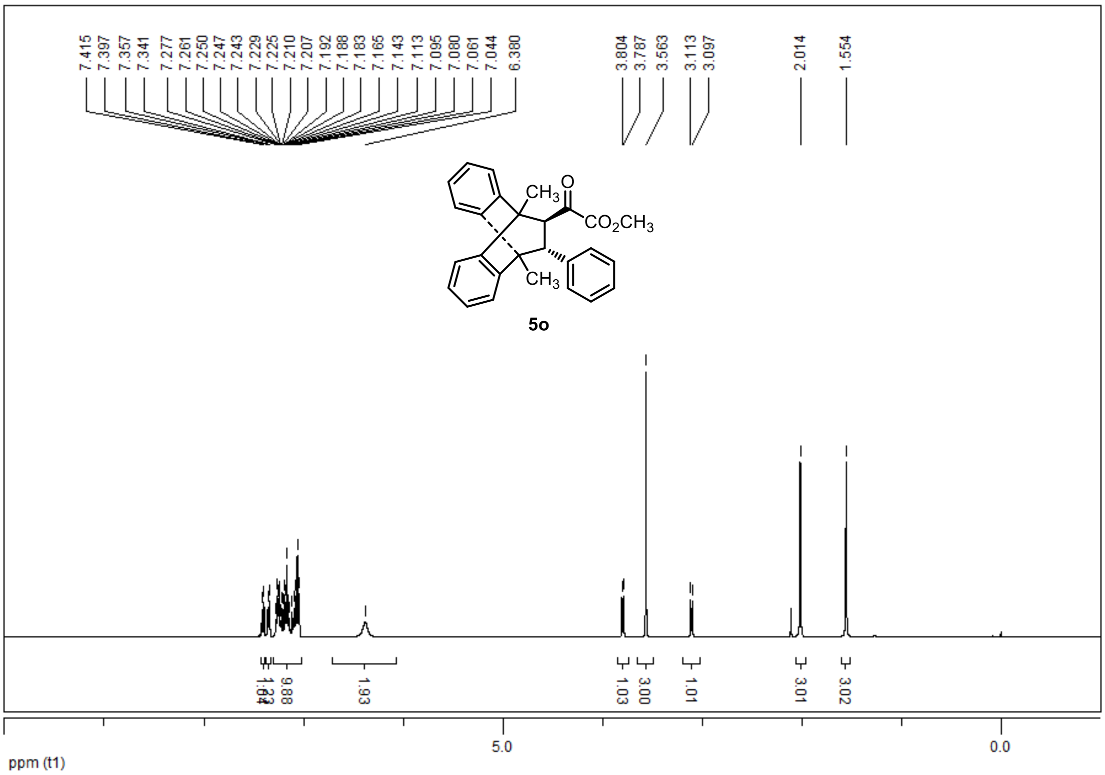
7.415
7.397
7.357
7.341
7.277
7.261
7.250
7.247
7.243
7.229
7.225
7.210
7.207
7.192
7.188
7.183
7.165
7.143
7.113
7.095
7.080
7.061
7.044
6.380
3.804
3.787
3.563
3.113
3.097
2.014
1.554
CH3
O
CO2CH3
CH3
5o
9.88
1.93
1.03
3.00
1.01
3.01
3.02
5.0
0.0
ppm (t1)

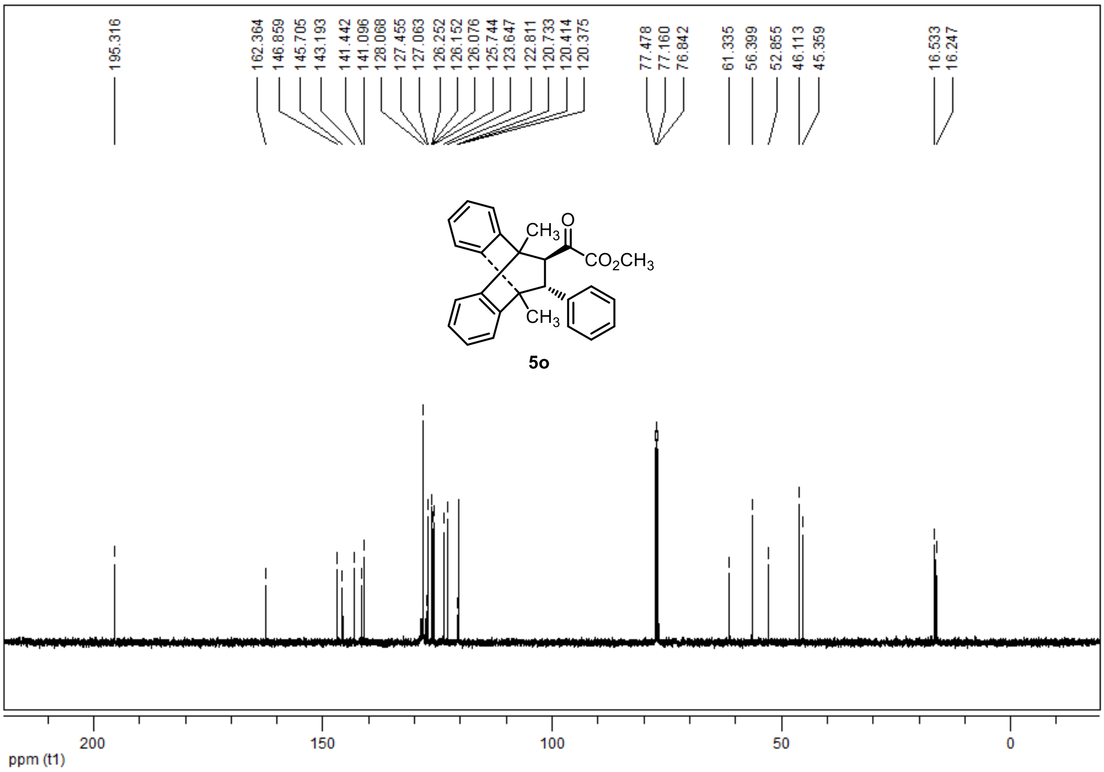
195.316
162.364
146.859
145.705
143.193
141.442
141.096
128.068
127.455
127.063
126.252
126.152
126.076
125.744
123.647
122.811
120.733
120.414
120.375
77.478
77.160
76.842
61.335
56.399
52.855
46.113
45.359
16.533
16.247
CH3
O
CO2CH3
CH3
5o
200
150
100
50
0
ppm (t1)

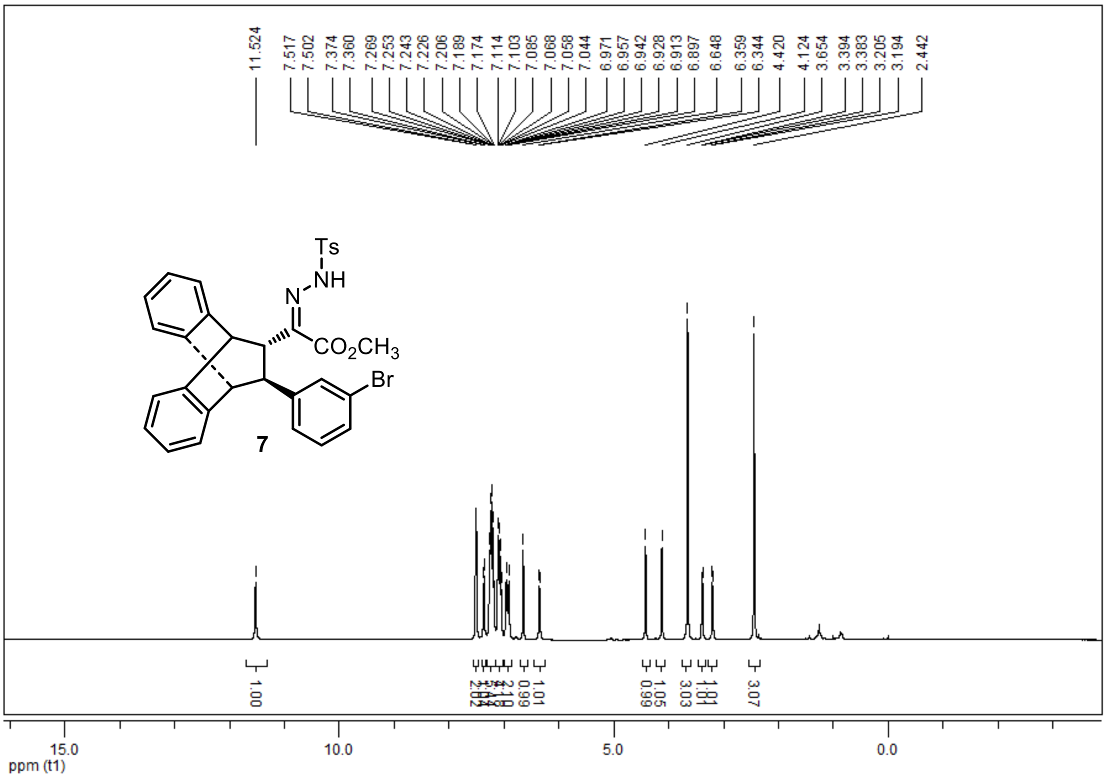
11.524
7.517
7.502
7.374
7.360
7.269
7.253
7.243
7.226
7.206
7.189
7.174
7.114
7.103
7.085
7.068
7.058
7.044
6.971
6.957
6.942
6.928
6.913
6.897
6.648
6.359
6.344
4.420
4.124
3.654
3.394
3.383
3.205
3.194
2.442
Ts
NH
N
CO2CH3
Br
7
1.00
0.99
1.01
0.99
1.05
3.03
3.07
15.0
10.0
5.0
0.0
ppm (t1)

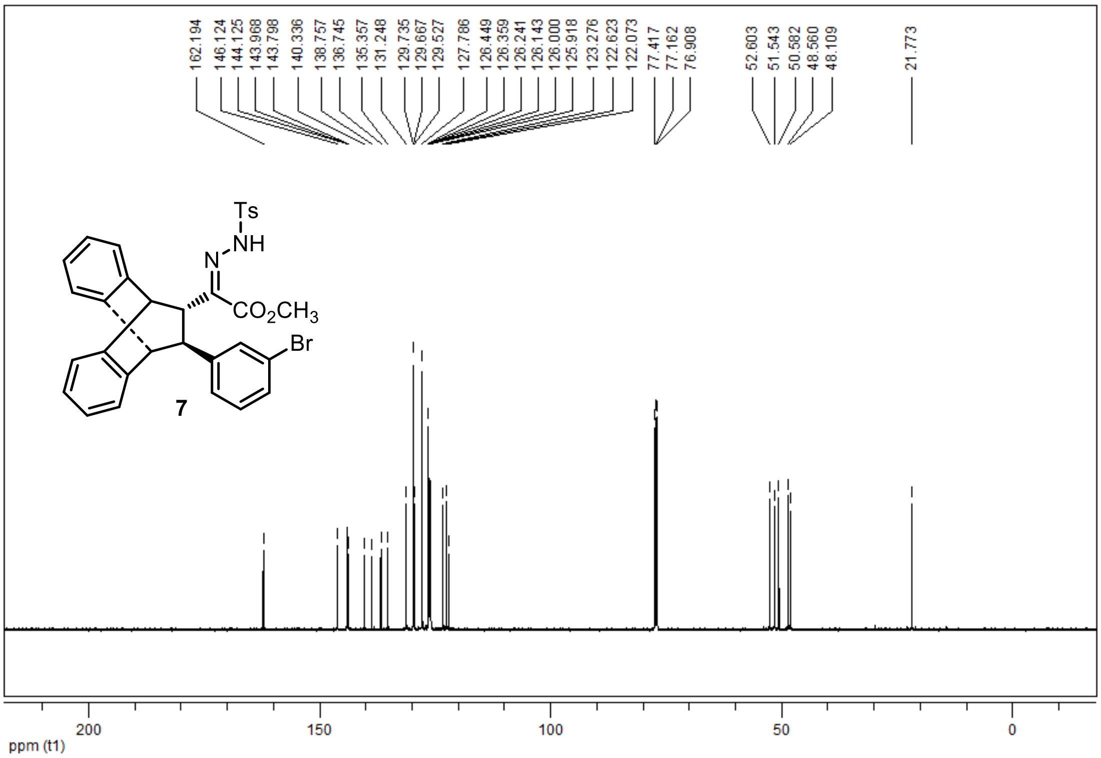
162.194
146.124
144.125
143.968
143.798
140.336
138.757
136.745
135.357
131.248
129.735
129.667
129.527
127.786
126.449
126.359
126.241
126.143
126.000
125.918
123.276
122.623
122.073
77.417
77.162
76.908
52.603
51.543
50.582
48.560
48.109
21.773
Ts
NH
N
CO2CH3
Br
7
200
150
100
50
0
ppm (t1)

HPLC Spectra

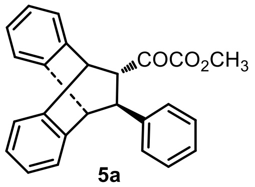

5a

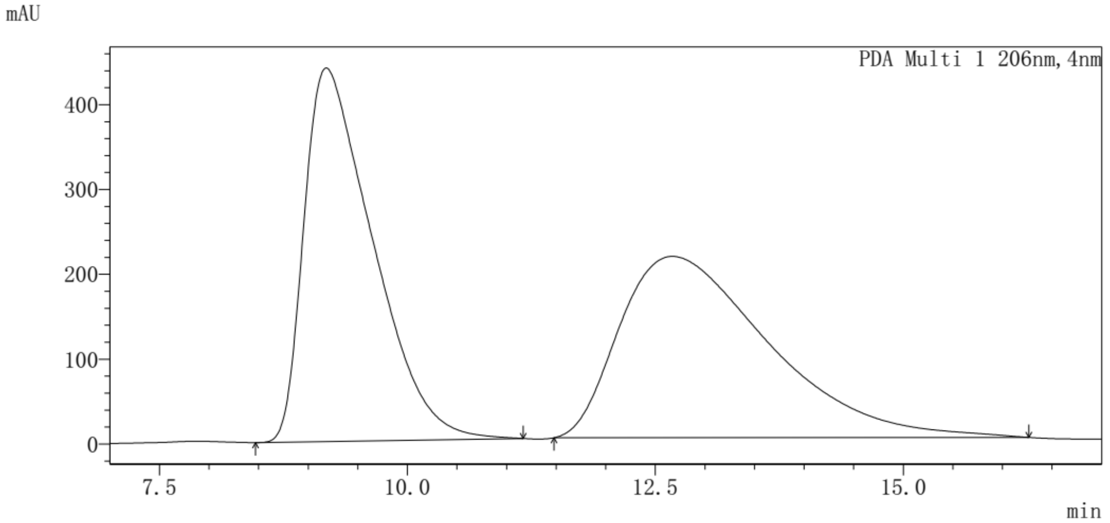

<Peak Results>

PDA Ch1 206nm

Index	Time/min	Height/mAU	Quantity/Area	Area %/%
1	9.181	440333	21247318	48.669
2	12.669	213771	22409639	51.331

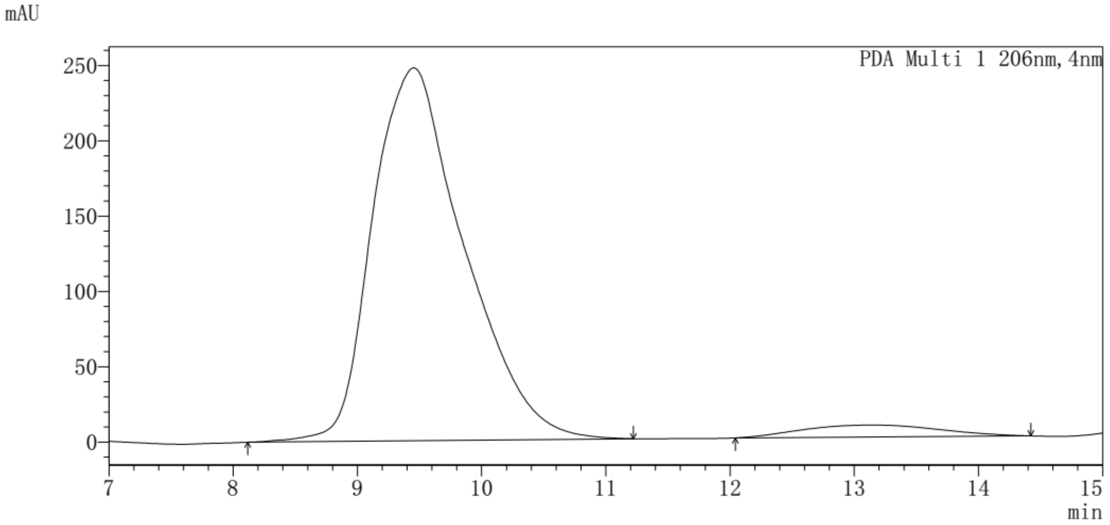

<Peak Results>

PDA Ch1 206nm

Index	Time/min	Height/mAU	Quantity/Area	Area %/%
1	9.454	247520	12712948	95.421
2	13.118	7960	610026	4.579

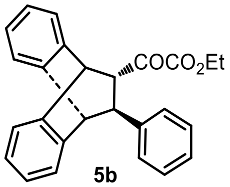

5b

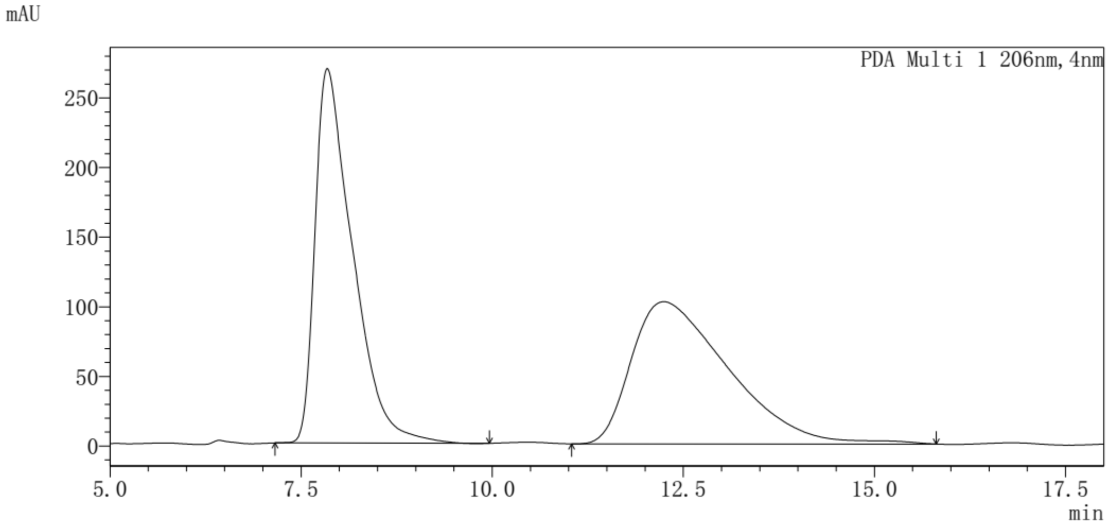

<Peak Results>

PDA Ch1 206nm

Index	Time/min	Height/mAU	Quantity/Area	Area %/%
1	7.843	268981	9186776	50.418
2	12.242	102174	9034453	49.582

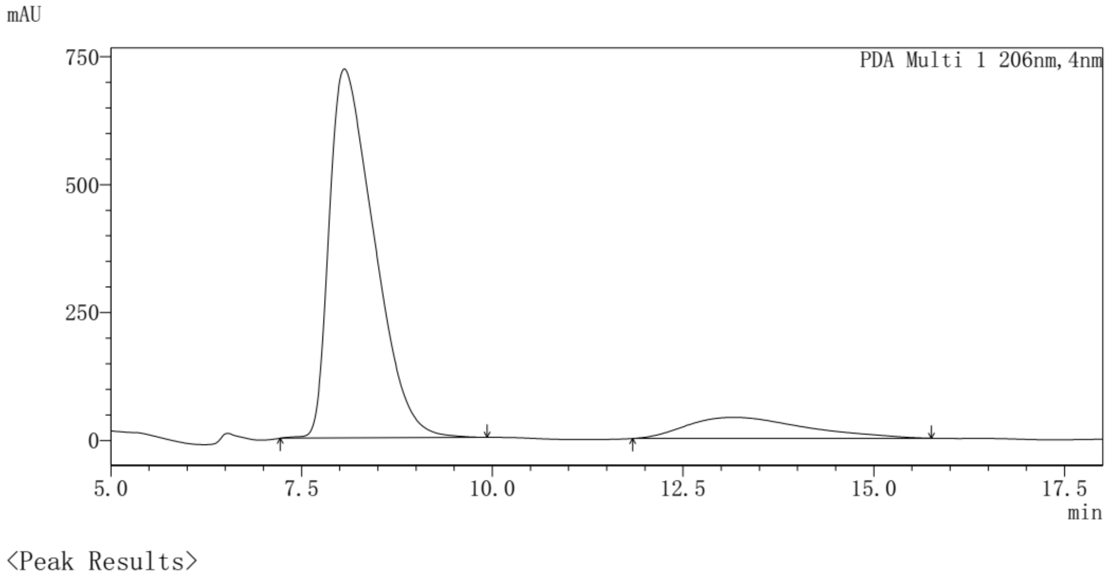

<Peak Results>

PDA Ch1 206nm

Index	Time/min	Height/mAU	Quantity/Area	Area %/%
1	8.061	720599	29467553	86.829
2	13.166	41188	4469754	13.171

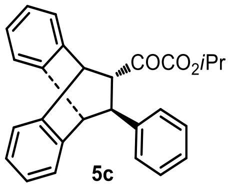

5c

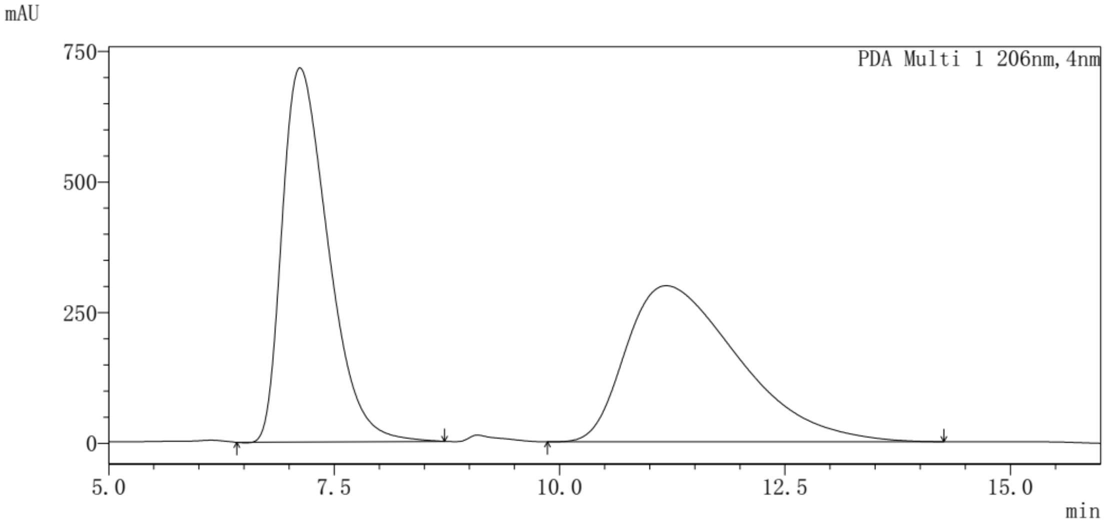

<Peak Results>

PDA Ch1 206nm

Index	Time/min	Height/mAU	Quantity/Area	Area %/%
1	7.119	716402	25480269	49.411
2	11.182	298863	26087405	50.589

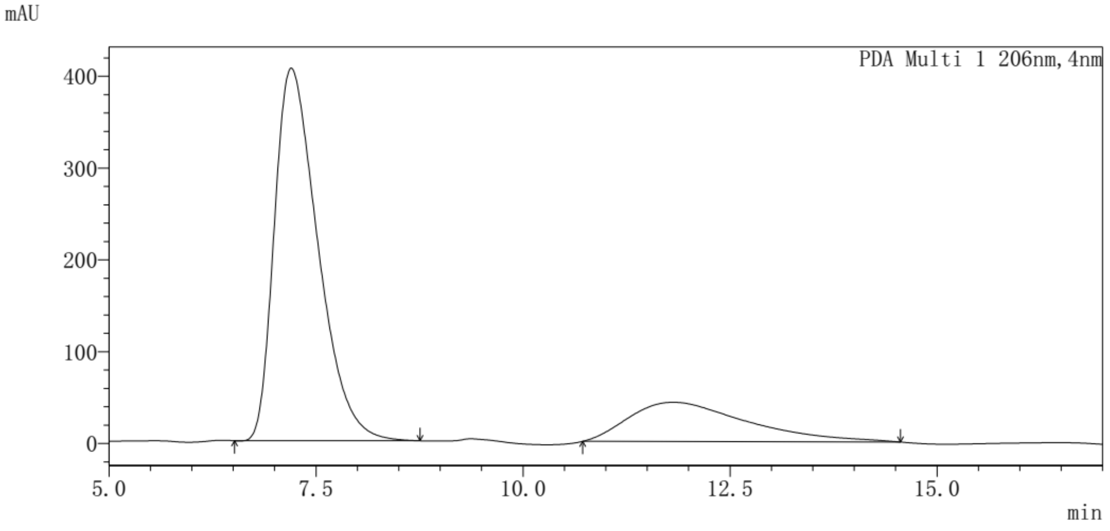

<Peak Results>

PDA Ch1 206nm

Index	Time/min	Height/mAU	Quantity/Area	Area %/%
1	7.201	406002	14844113	77.688
2	11.822	42816	4263235	22.312

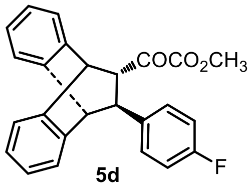

5d

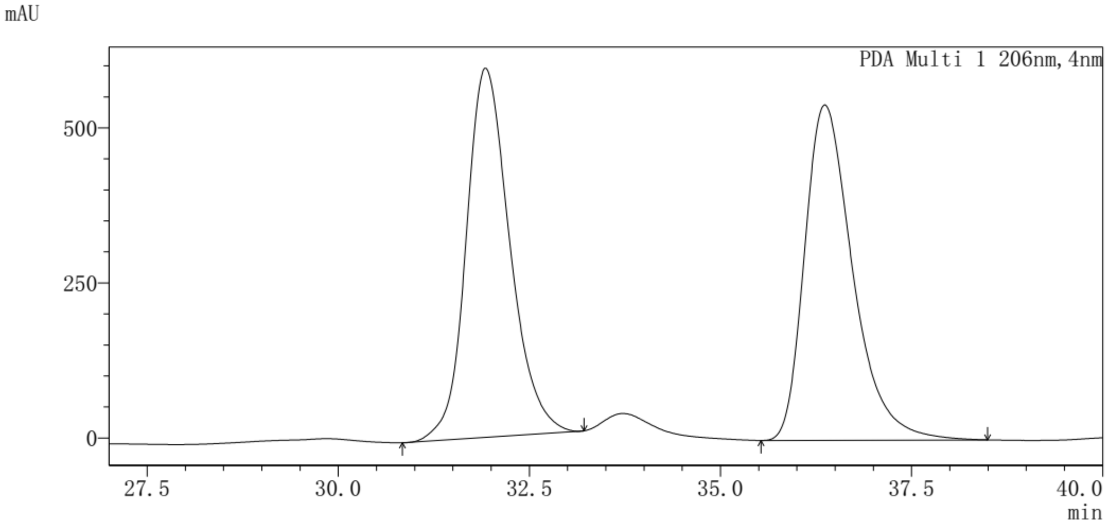

<Peak Results>

PDA Ch1 206nm

Index	Time/min	Height/mAU	Quantity/Area	Area %/%
1	31.925	595421	23645482	49.952
2	36.364	540898	23690963	50.048

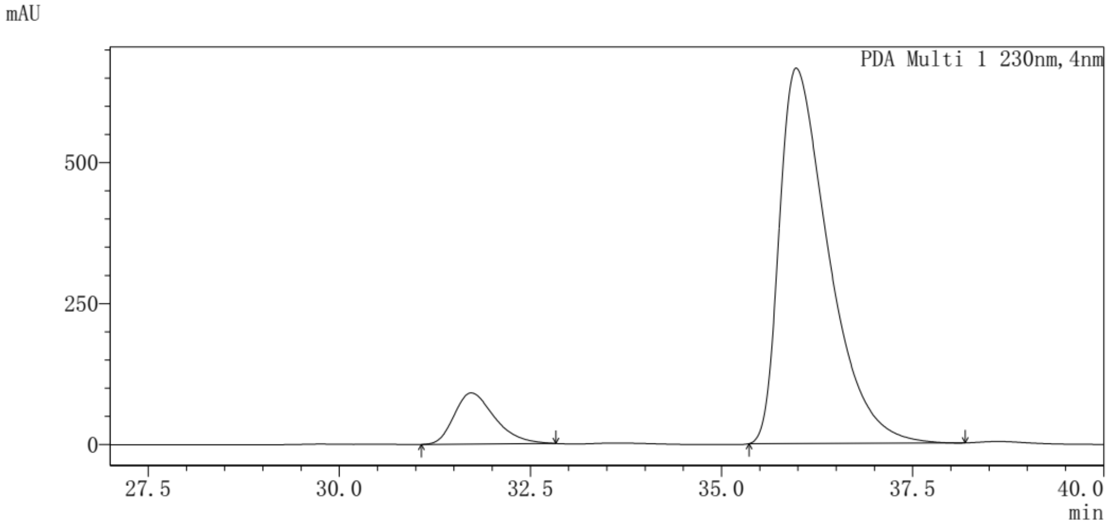

<Peak Results>

PDA Ch1 230nm

Index	Time/min	Height/mAU	Quantity/Area	Area %/%
1	31.727	90762	3346172	10.114
2	35.978	665705	29736945	89.886

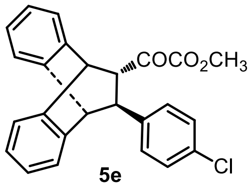

5e

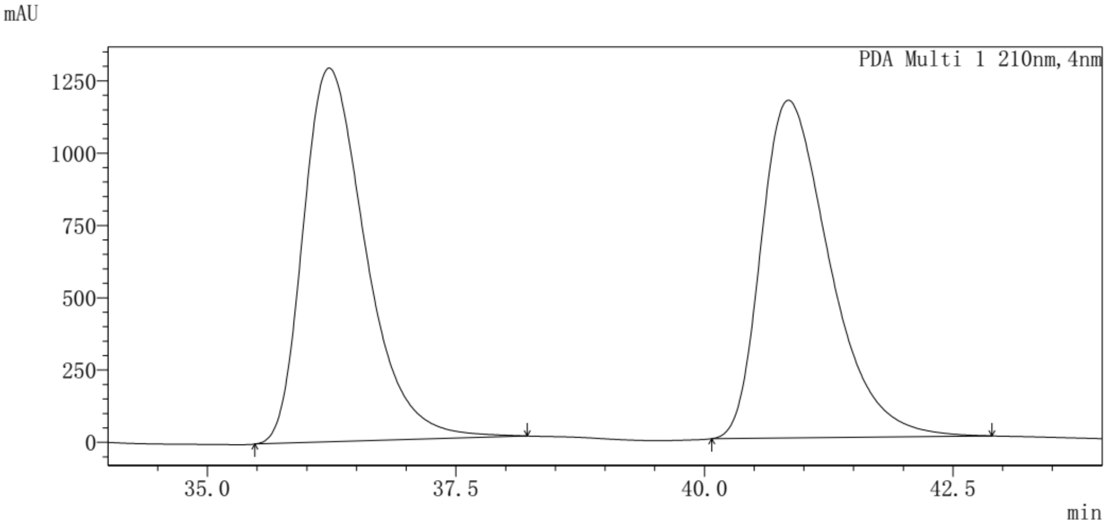

<Peak Results>

PDA Ch1 210nm

Index	Time/min	Height/mAU	Quantity/Area	Area %/%
1	36.224	1292603	56691467	50.213
2	40.844	1167899	56209909	49.787

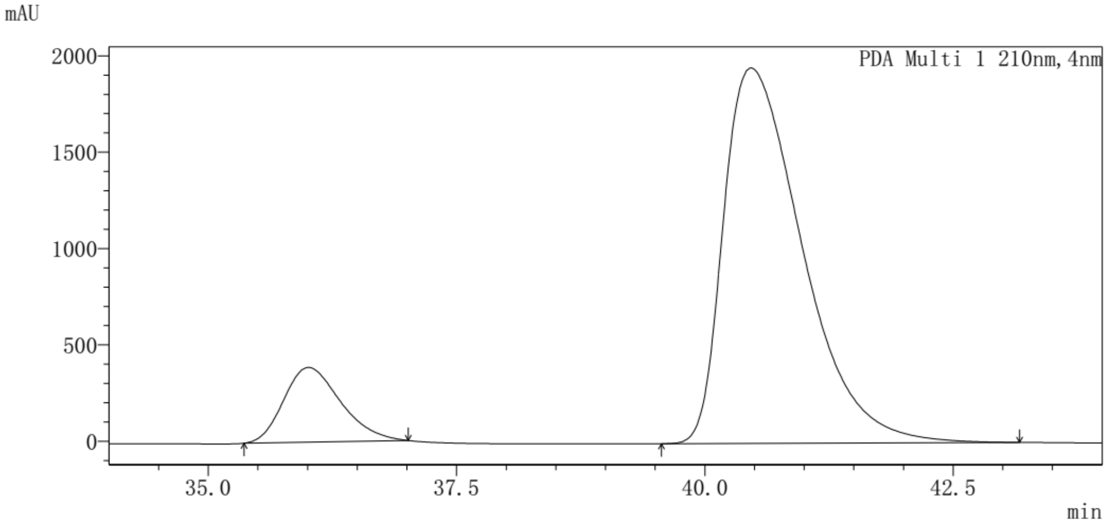

<Peak Results>

PDA Ch1 210nm

Index	Time/min	Height/mAU	Quantity/Area	Area %/%
1	36.010	387751	15448626	12.572
2	40.469	1948797	107428429	87.428

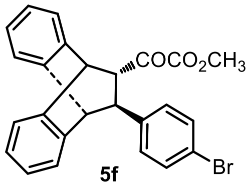

5f

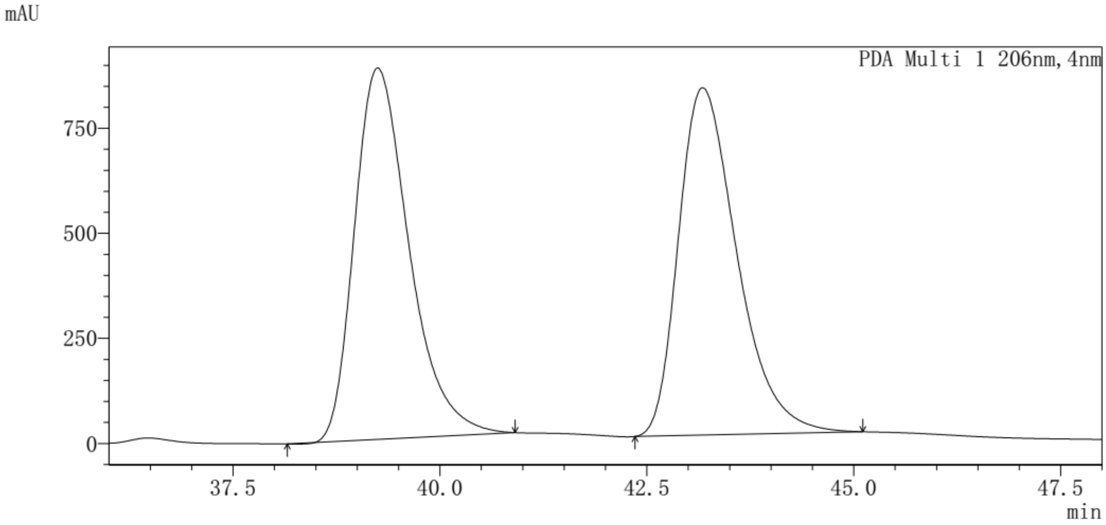

〈Peak Results〉

PDA Ch1 206nm

Index	Time/min	Height/mAU	Quantity/Area	Area %/%
1	39.247	884106	40573007	50.001
2	43.174	826677	40572136	49.999

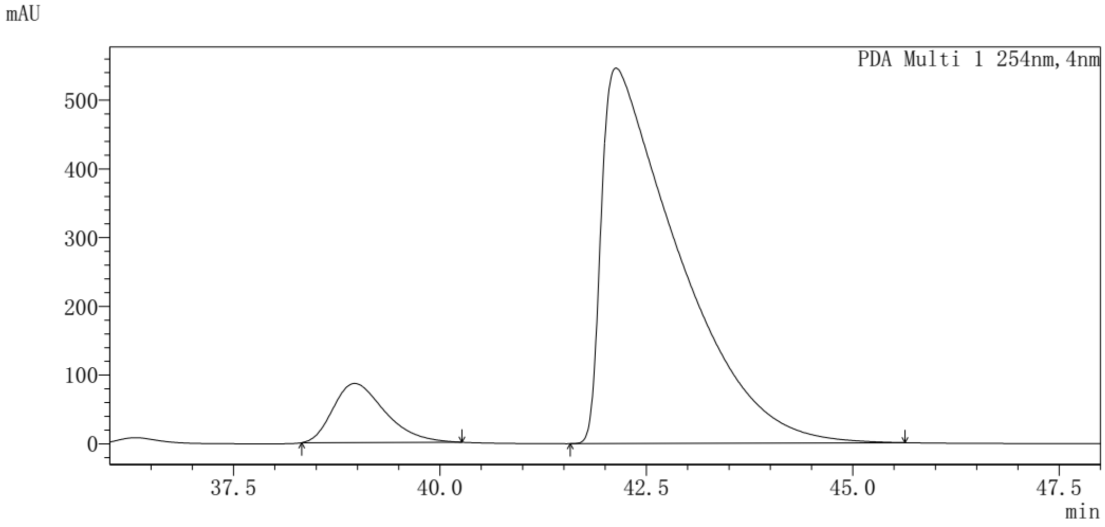

〈Peak Results〉

PDA Ch1 254nm

Index	Time/min	Height/mAU	Quantity/Area	Area %/%
1	38.964	86055	3762892	9.556
2	42.131	546441	35612485	90.444

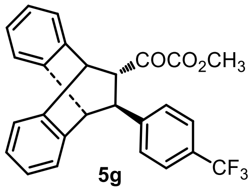

5g

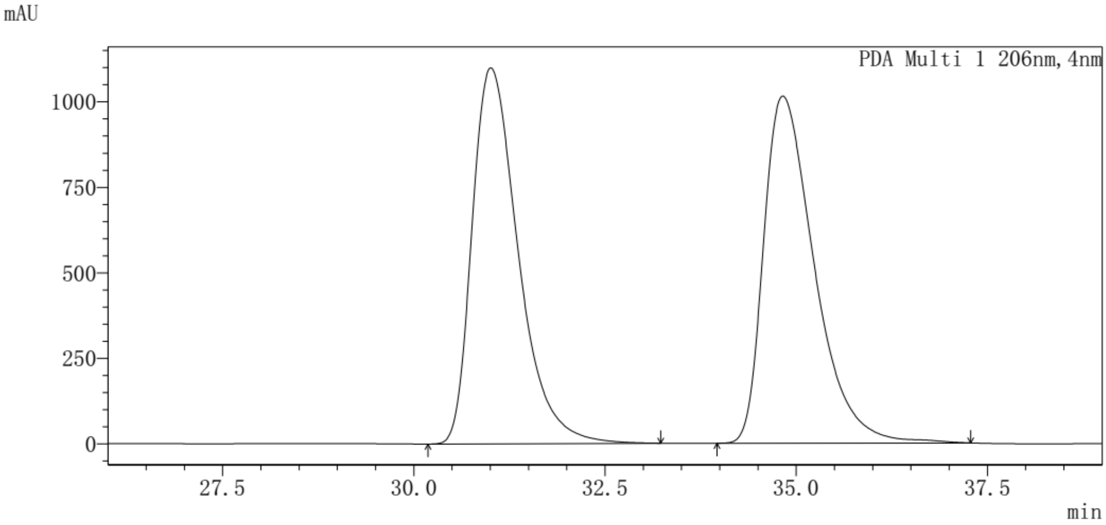

<Peak Results>

PDA Ch1 206nm

Index	Time/min	Height/mAU	Quantity/Area	Area %/%
1	31.007	1099049	46113008	49.621
2	34.824	1014885	46818102	50.379

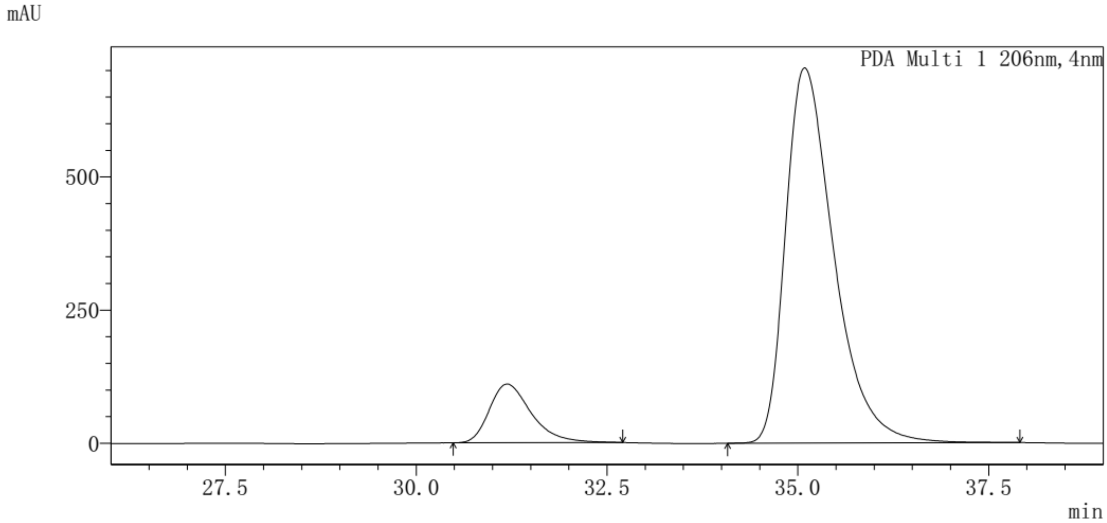

<Peak Results>

PDA Ch1 206nm

Index	Time/min	Height/mAU	Quantity/Area	Area %/%
1	31.191	110353	4221185	11.967
2	35.089	704606	31053812	88.033

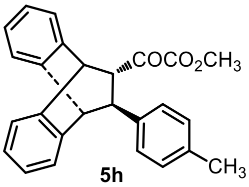

5h

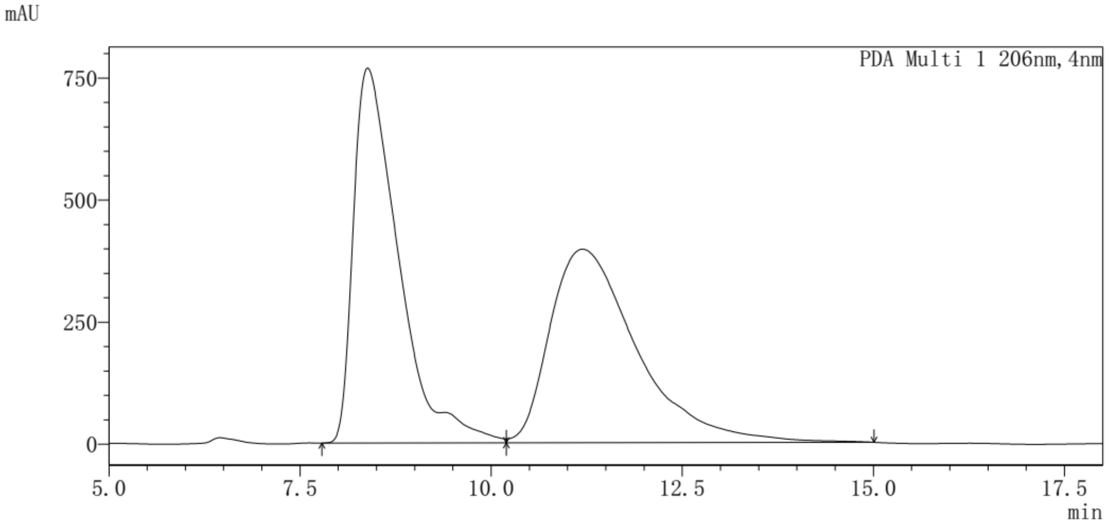

<Peak Results>
PDA Ch1 206nm

Index	Time/min	Height/mAU	Quantity/Area	Area %/%
1	8.383	767827	31416617	49.975
2	11.199	396346	31448599	50.025

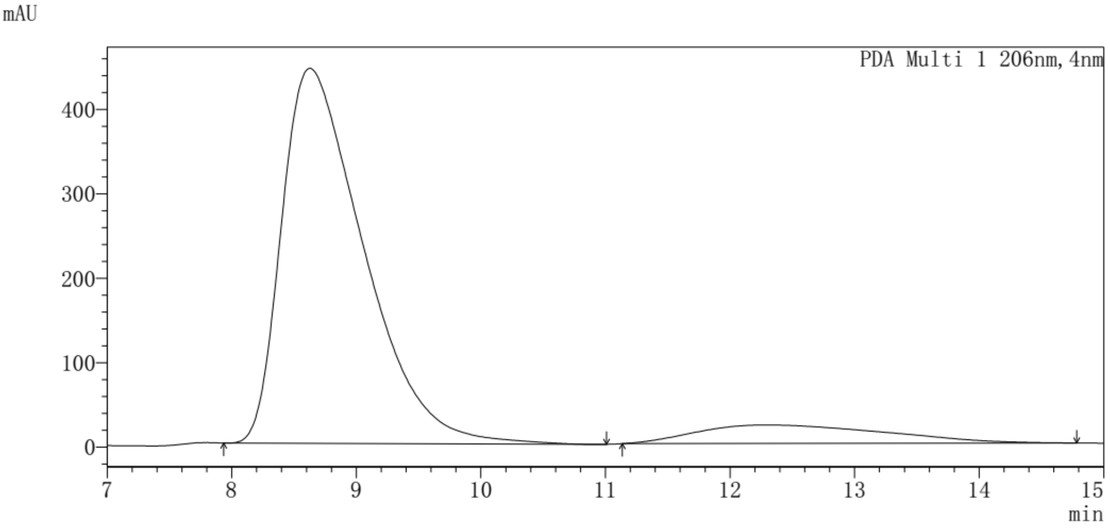

<Peak Results>
PDA Ch1 206nm

Index	Time/min	Height/mAU	Quantity/Area	Area %/%
1	8.627	444257	20331403	89.811
2	12.280	21930	2306487	10.189

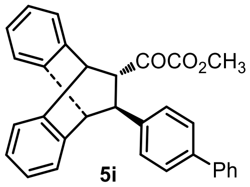

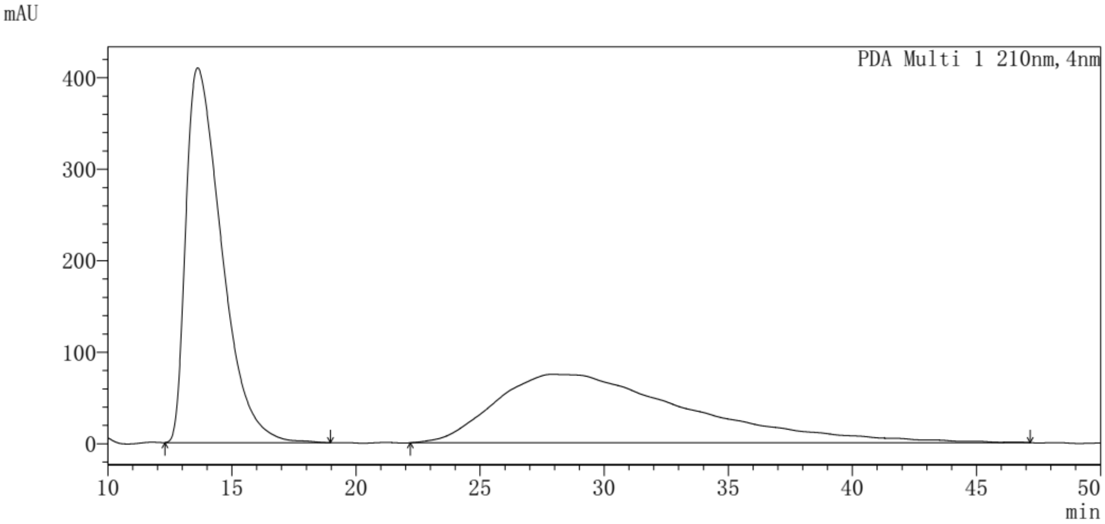

〈Peak Results〉

PDA Ch1 210nm

Index	Time/min	Height/mAU	Quantity/Area	Area %/%
1	13.617	409603	41067371	50.476
2	27.893	74839	40292421	49.524

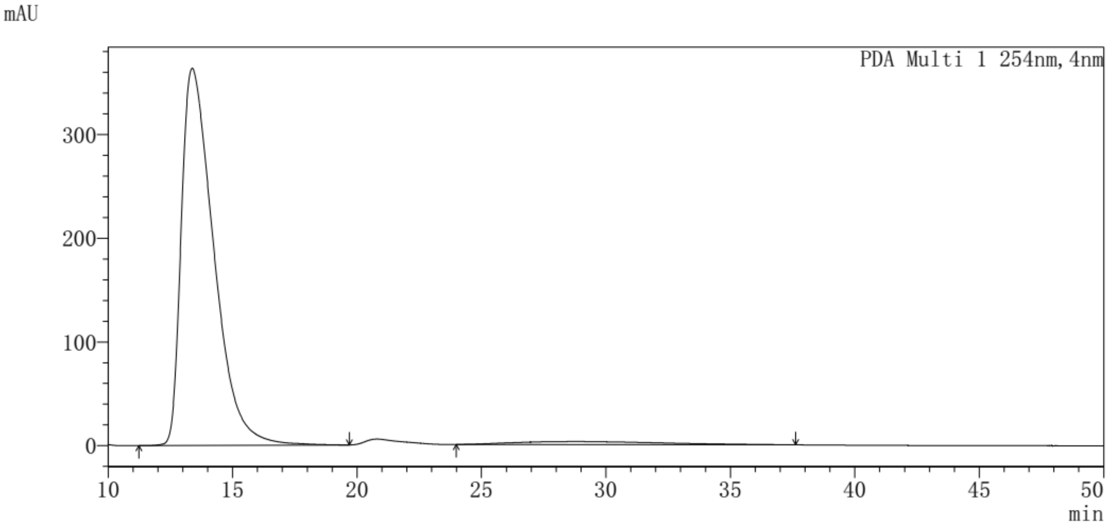

〈Peak Results〉

PDA Ch1 254nm

Index	Time/min	Height/mAU	Quantity/Area	Area %/%
1	13.378	363706	32931600	96.380
2	28.830	2986	1236955	3.620

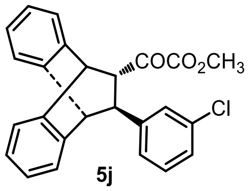

5j

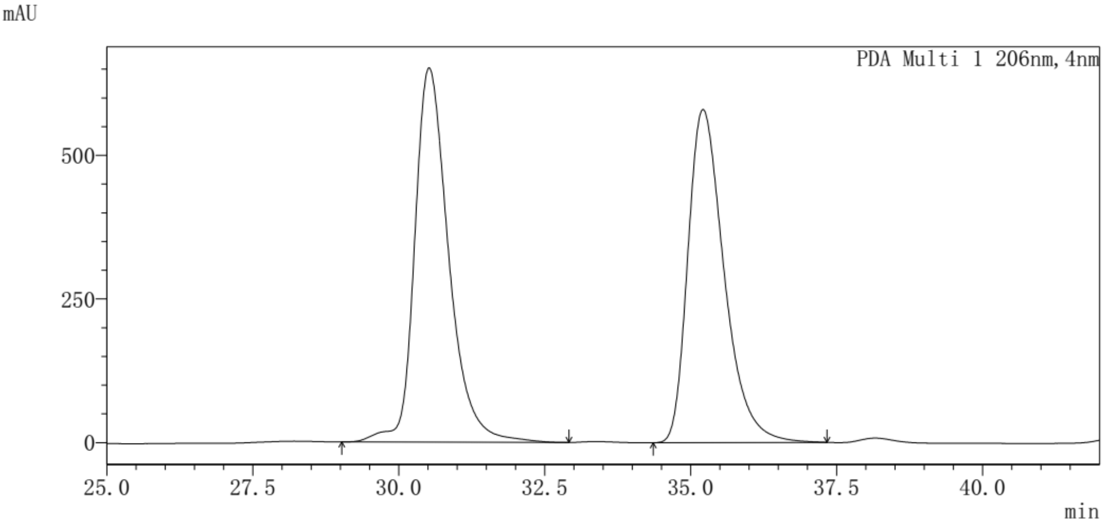

<Peak Results>

PDA Ch1 206nm

Index	Time/min	Height/mAU	Quantity/Area	Area %/%
1	30.519	651739	26535831	51.426
2	35.210	580219	25064688	48.574

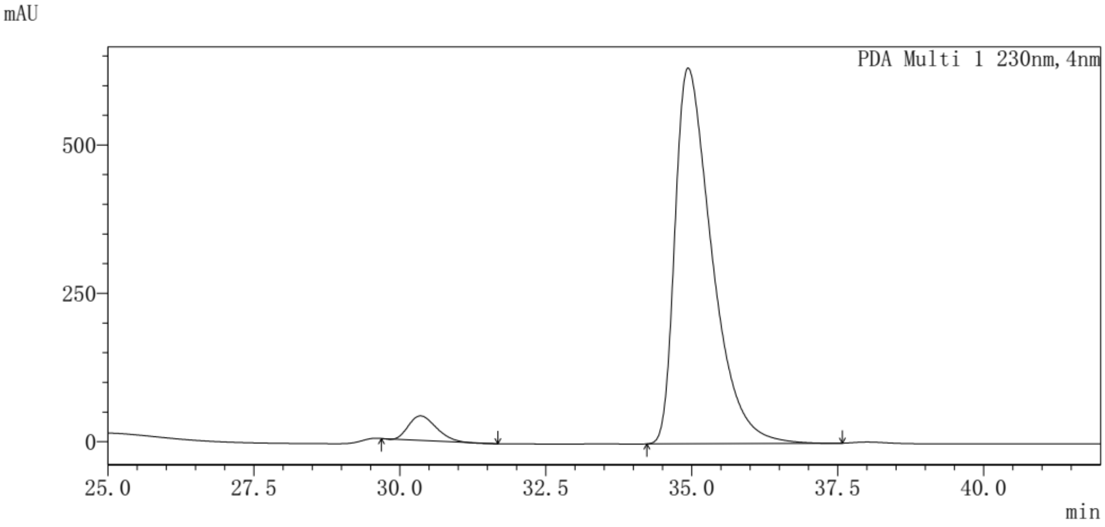

<Peak Results>

PDA Ch1 230nm

Index	Time/min	Height/mAU	Quantity/Area	Area %/%
1	30.353	41177	1330066	4.568
2	34.937	633255	27790162	95.432

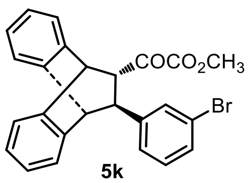

5k

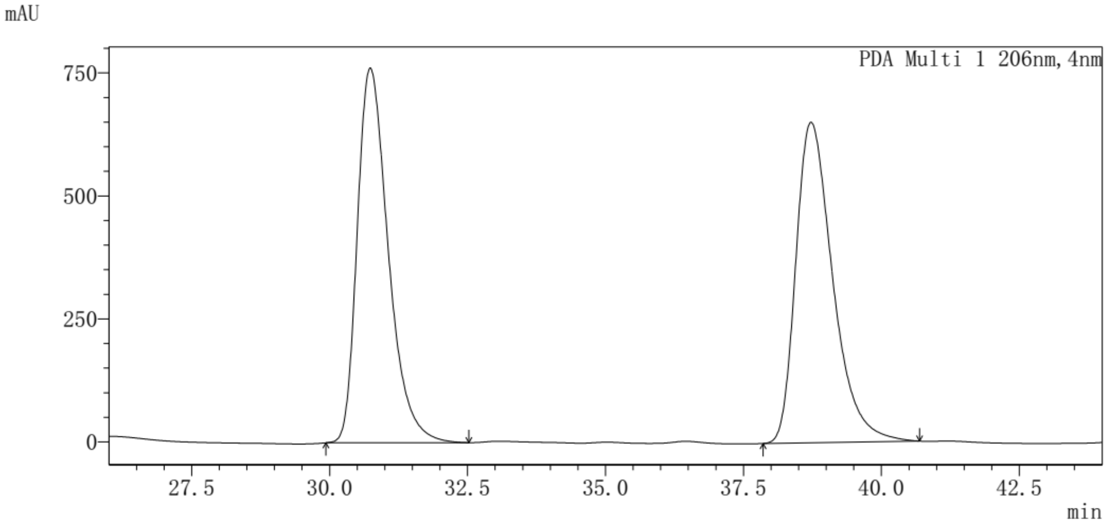

<Peak Results>

PDA Ch1 206nm

Index	Time/min	Height/mAU	Quantity/Area	Area %/%
1	30.735	761910	29784586	49.535
2	38.724	651779	30343271	50.465

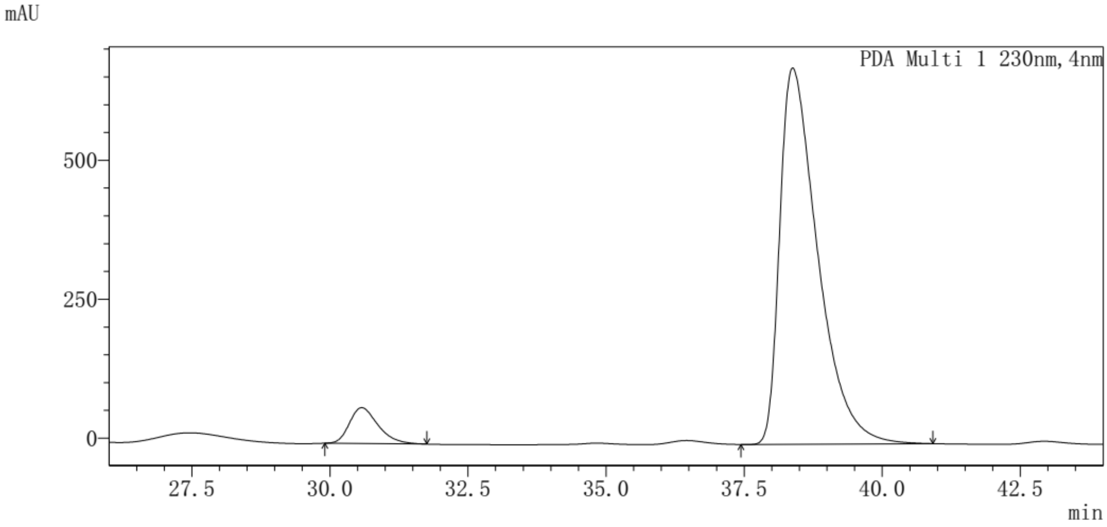

<Peak Results>

PDA Ch1 230nm

Index	Time/min	Height/mAU	Quantity/Area	Area %/%
1	30.574	64523	2329933	6.714
2	38.379	677163	32372519	93.286

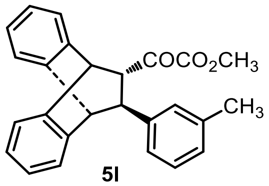

5l

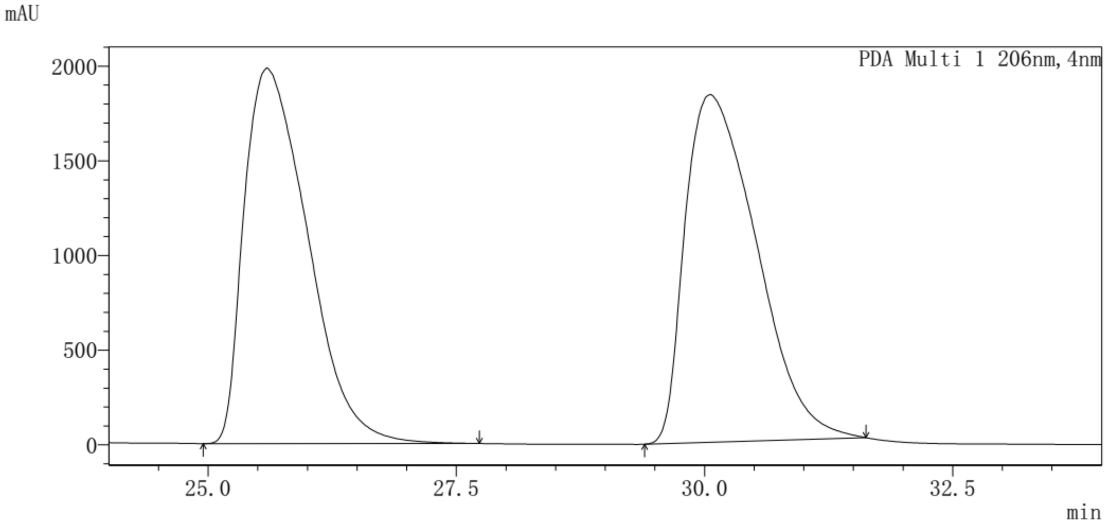

<Peak Results>

PDA Ch1 206nm

Index	Time/min	Height/mAU	Quantity/Area	Area %/%
1	25.593	1983723	88419646	48.983
2	30.061	1836493	92092492	51.017

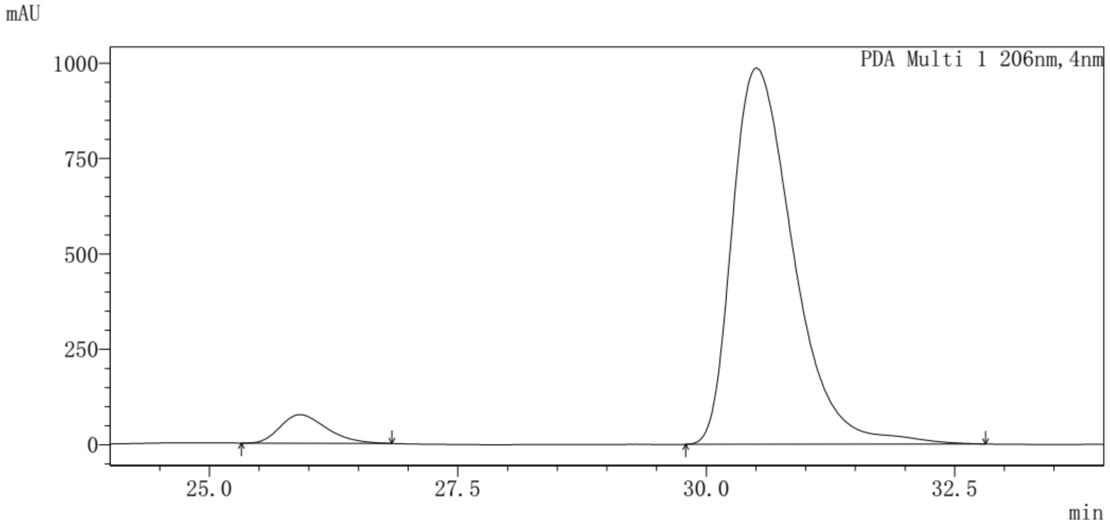

<Peak Results>

PDA Ch1 206nm

Index	Time/min	Height/mAU	Quantity/Area	Area %/%
1	25.912	74891	2439703	5.471
2	30.505	985695	42156800	94.529

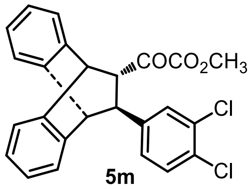

5m

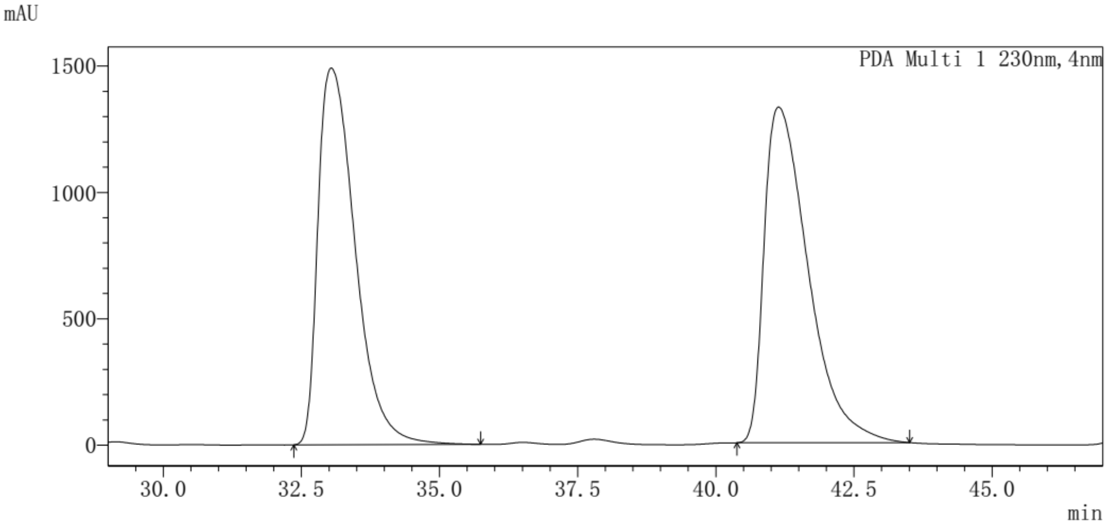

<Peak Results>

PDA Ch1 230nm

Index	Time/min	Height/mAU	Quantity/Area	Area %/%
1	33.039	1490359	69674211	48.611
2	41.135	1328321	73655228	51.389

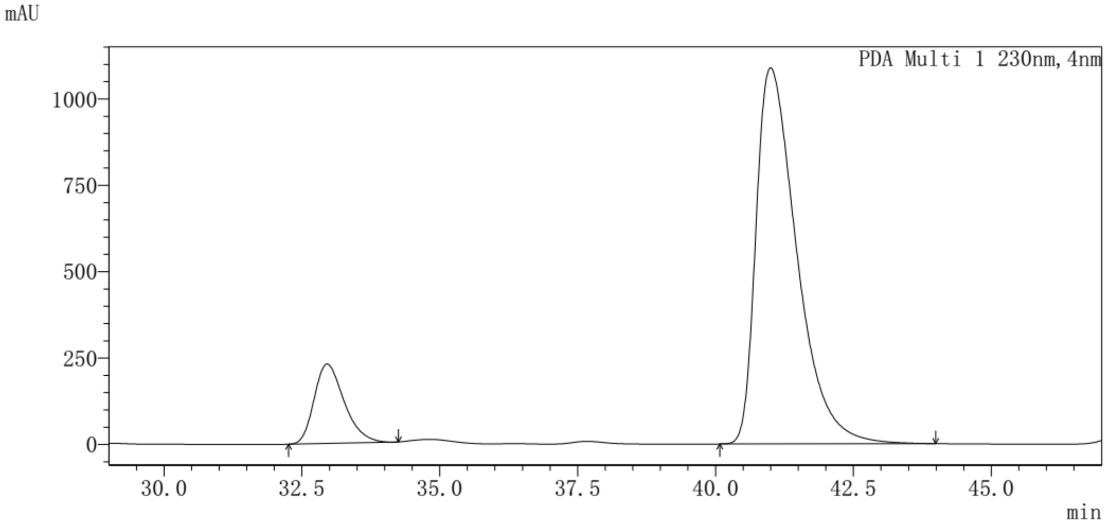

<Peak Results>

PDA Ch1 230nm

Index	Time/min	Height/mAU	Quantity/Area	Area %/%
1	32.956	229901	8694359	13.447
2	40.997	1088312	55961394	86.553

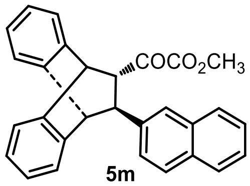

5m

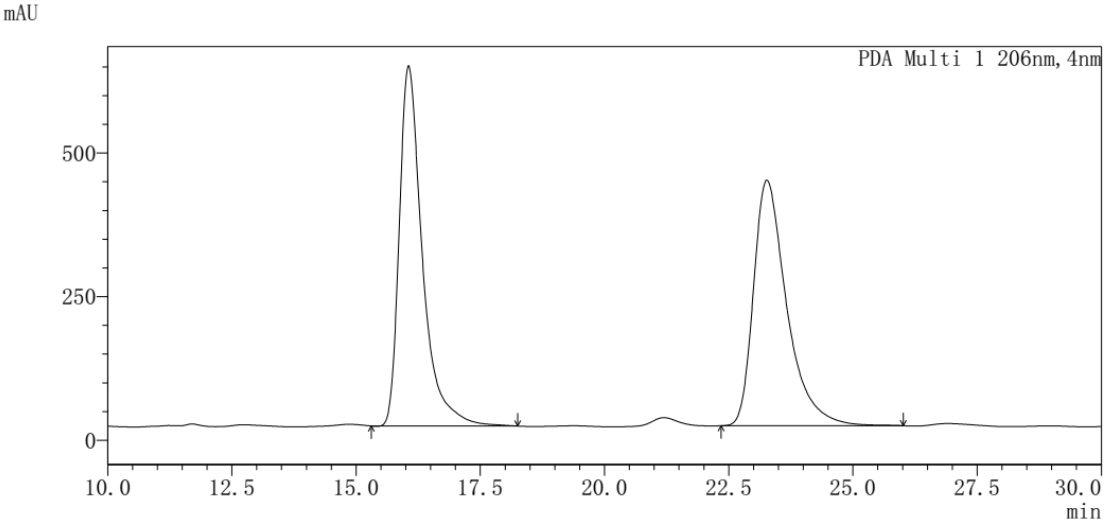

〈Peak Results〉

PDA Ch1 206nm

Index	Time/min	Height/mAU	Quantity/Area	Area %/%
1	16.056	627084	20266235	50.445
2	23.266	427529	19908796	49.555

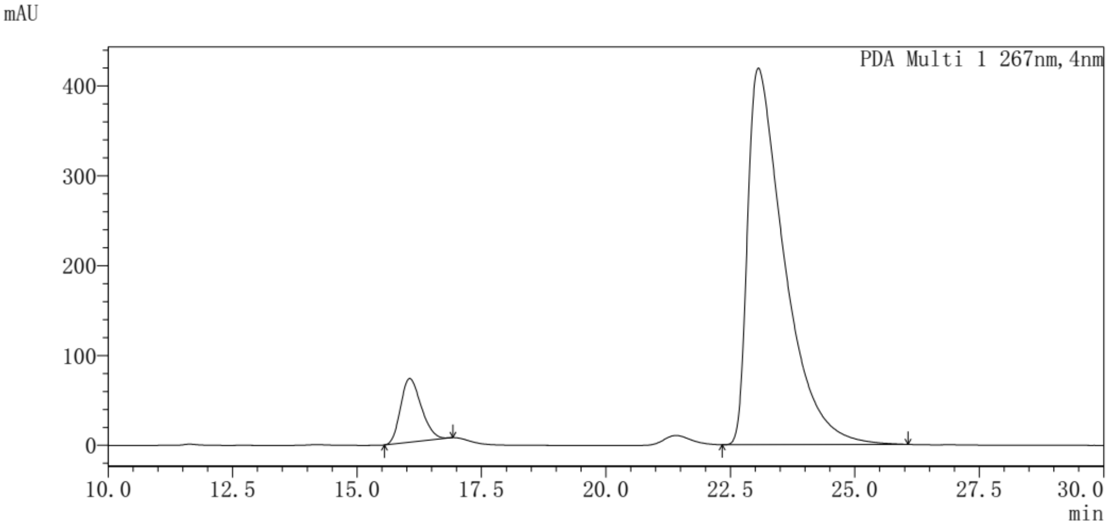

〈Peak Results〉

PDA Ch1 267nm

Index	Time/min	Height/mAU	Quantity/Area	Area %/%
1	16.059	71061	2051838	8.700
2	23.062	419346	21533171	91.300

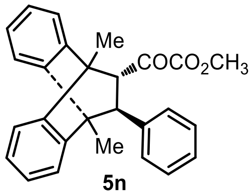

5n

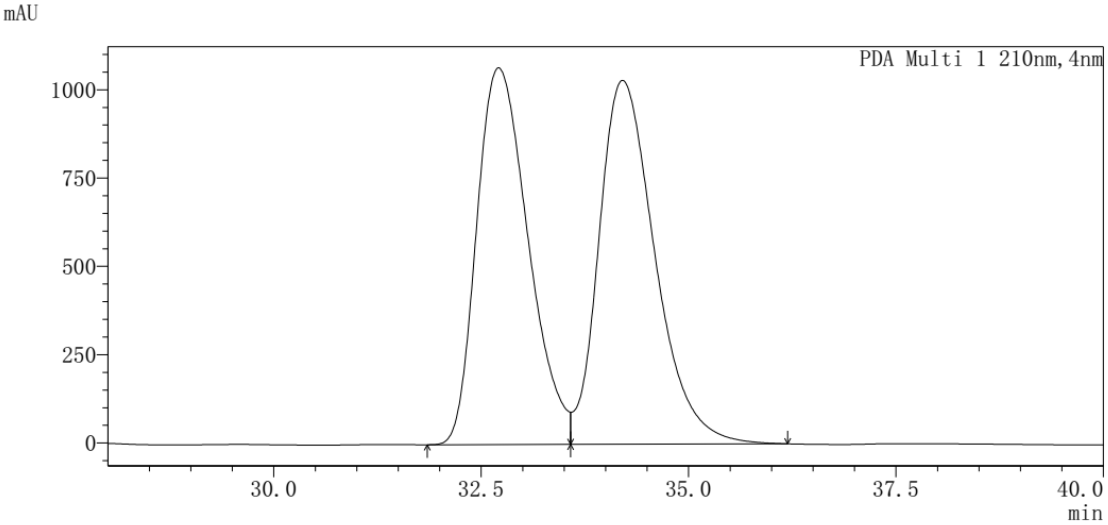

⟨Peak Results⟩

PDA Ch1 210nm

Index	Time/min	Height/mAU	Quantity/Area	Area %/%
1	32.709	1066540	46063802	48.780
2	34.203	1030215	48367155	51.220

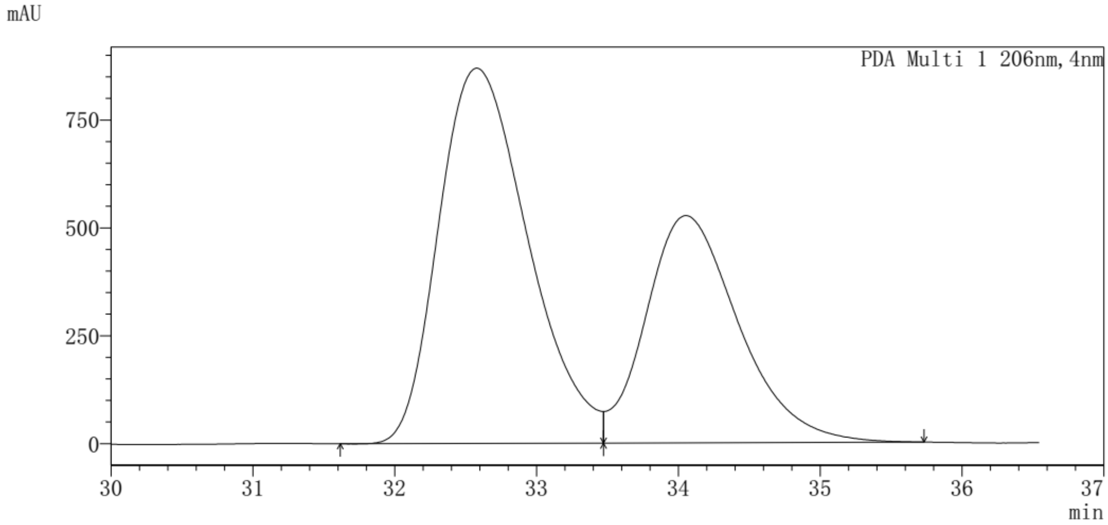

⟨Peak Results⟩

PDA Ch1 206nm

Index	Time/min	Height/mAU	Quantity/Area	Area %/%
1	32.577	869874	38127719	61.407
2	34.053	526539	23962041	38.593

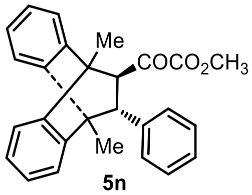

5n

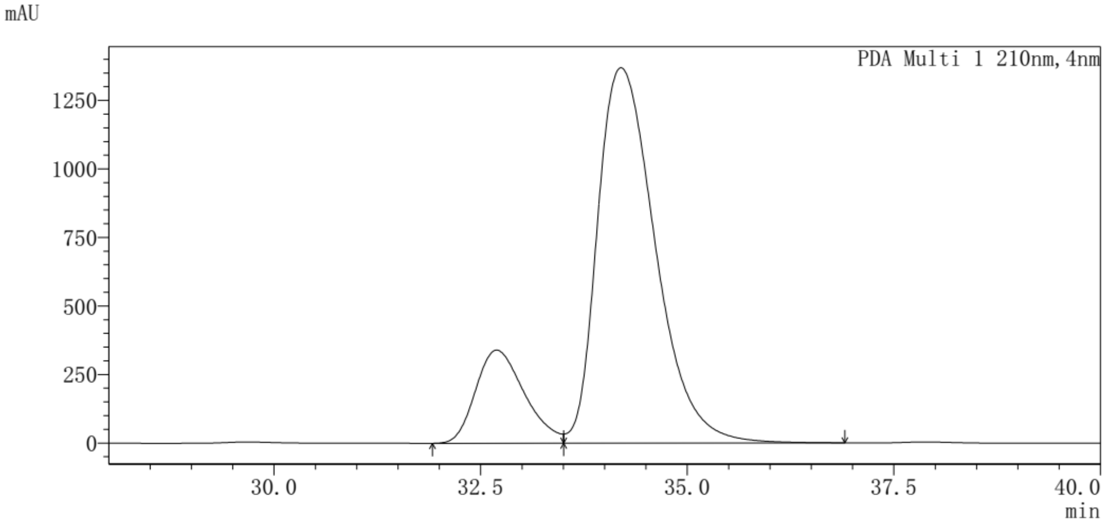

<Peak Results>

PDA Ch1 210nm

Index	Time/min	Height/mAU	Quantity/Area	Area %/%
1	32.693	339816	13941973	17.288
2	34.197	1369764	66703674	82.712

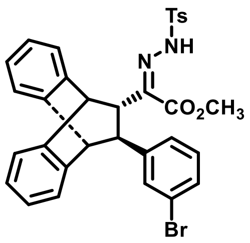

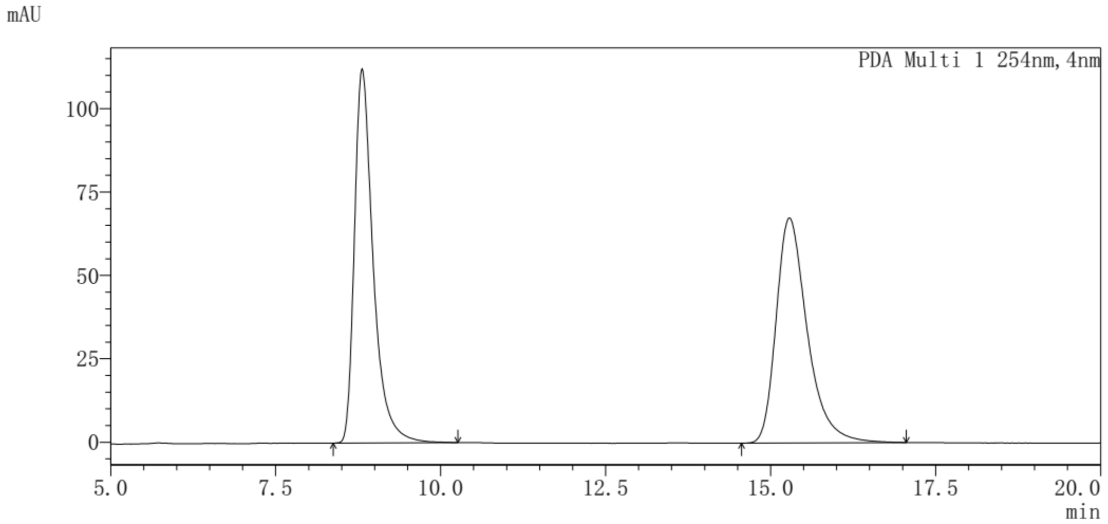

<Peak Results>

PDA Ch1 254nm

Index	Time/min	Height/mAU	Quantity/Area	Area %/%
1	8.809	112158	2218414	50.019
2	15.284	67531	2216685	49.981

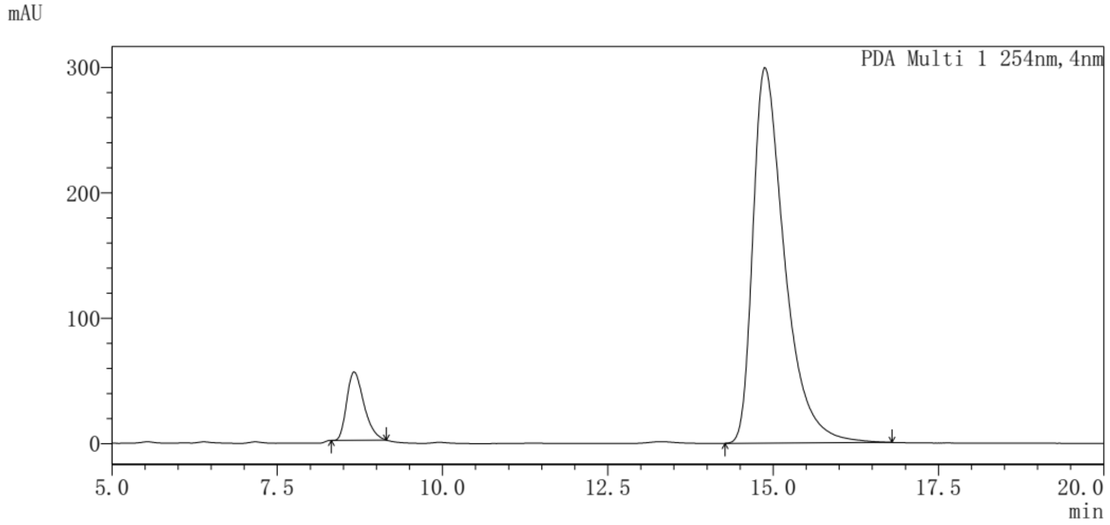

<Peak Results>

PDA Ch1 254nm

Index	Time/min	Height/mAU	Quantity/Area	Area %/%
1	8.661	54495	988047	9.017
2	14.874	299383	9969296	90.983
